# Towards the “Eldorado” of pKa Determination: A Reliable and Rapid DFT Model

**DOI:** 10.3390/molecules29061255

**Published:** 2024-03-12

**Authors:** Silvia Pezzola, Mariano Venanzi, Pierluca Galloni, Valeria Conte, Federica Sabuzi

**Affiliations:** Department of Chemical Science and Technologies, University of Rome Tor Vergata, Via della Ricerca Scientifica, 00133 Roma, Italy; silvia.pezzola@uniroma2.it (S.P.); venanzi@uniroma2.it (M.V.); galloni@scienze.uniroma2.it (P.G.)

**Keywords:** pKa, computational pKa, carboxylic acid, Jacob’s ladder, TPSSTPSS, PEBE1PBE, B3PW91, PBEPBE, WB97XD, CAM-B3LYP, direct approach, DFT, solvation model based on density (SMD)

## Abstract

The selection of a “perfect tool” for the theoretical determination of acid-base dissociation constants (Ka) is still puzzling. Recently, we developed a user-friendly model exploiting CAM-B3LYP for determining pKa with impressive reliability. Herein, a new challenge is faced, examining a panel of functionals belonging to different rungs of the “Jacob’s ladder” organization, which classifies functionals according to their level of theory. Specifically, meta-generalized gradient approximations (GGAs), hybrid-GGAs, and the more complex range-separated hybrid (RSH)-GGAs were investigated in predicting the pKa of differently substituted carboxylic acids. Therefore, CAM-B3LYP, WB97XD, B3PW91, PBE1PBE, PBEPBE and TPSSTPSS were used, with 6-311G+(d,p) as the basis set and the solvation model based on density (SMD). CAM-B3LYP showed the lowest mean absolute error value (MAE = 0.23) with relatively high processing time. PBE1PBE and B3PW91 provided satisfactory predictions (MAE = 0.34 and 0.38, respectively) with moderate computational time cost, while PBEPBE, TPSSTPSS and WB97XD led to unreliable results (MAE > 1). These findings validate the reliability of our model in predicting carboxylic acids pKa, with MAE well below 0.5 units, using a simplistic theoretical level and a low-cost computational approach.

## 1. Introduction

Several efforts have been pursued in computational chemistry to determine the acid dissociation constant (Ka) of organic compounds in aqueous solution [1]. The advantages of the *in silico* pKa determination are connected with the development of environment-friendly and rapid approaches that avoid experimental and costly procedures, in particular when dealing with structurally complex molecules [1,2]. Achieving high reliability in pKa calculation is still challenging, due to the recurrent discrepancies between experimental and computational results, which sensitively depend on the chosen level of theory, the density functional and the adopted solvation model [2,3]. The computational determination of pKa is usually performed through several methodologies, varying from the pioneering “machine learning” approach, to the more traditional physicochemical-inspired models. The former is focused on the quantitative structure–activity relationship (thereafter QSAR), but although it generally requires huge computational costs, it has not yet shown good reliability [4]. The latter relies on solid computational methods [5,6] that can be classified into two categories: (i) the “indirect” approach, which exploits a thermodynamic cycle that takes into consideration the energy at equilibrium of the deprotonated species in the gas and in the solution phase; (ii) the “direct” approach, in which the ionogenic equation in water is explicitly considered [5,6]. In computing carboxylic acids pKa, the direct approach is widely used. Indeed, according to the literature, a variable amount of water molecules, ranging from one to eight, can be merged into the reaction center to faithfully mimic the first solvation shell [2]. In a recent example [7], authors calculated the pKa of a series of differently substituted carboxylic acids using a linear correlation fit that includes the H-bond length between water molecules and the acid and/or the conjugated base involved at the reaction center. Among the others, B3LYP resulted in the most appropriate functional, coupled with a 6-31G+(d,p) basis set, in the aqueous continuum model. Other approaches have delved into mixed levels of theory, such as DFT, Hartree–Fock (HF) and/or Møller–Plesset (MP), to calculate *in silico* carboxylic acids pKa. Indeed, B3LYP was directly compared with M05-2X or HF [5]. The authors inserted four water molecules at the reaction center and a radii correction factor to pursue more reliable outputs. Starting from these assumptions, additional works investigated the effects of re-shaping the atoms’ radii and the relative position of the involved species, keeping the solvation model constant. Thus, it has been demonstrated that the geometry of the acid moiety played a fundamental role in obtaining consistent calculated pKa values, while the selected continuum model barely affects method reliability [5,6,7]. Nevertheless, such procedures, independently from the applied level of theory, still require mathematical fittings, the use of internal correction coefficients, and/or H^+^ experimental energy values [1,8].

**Figure 1:**
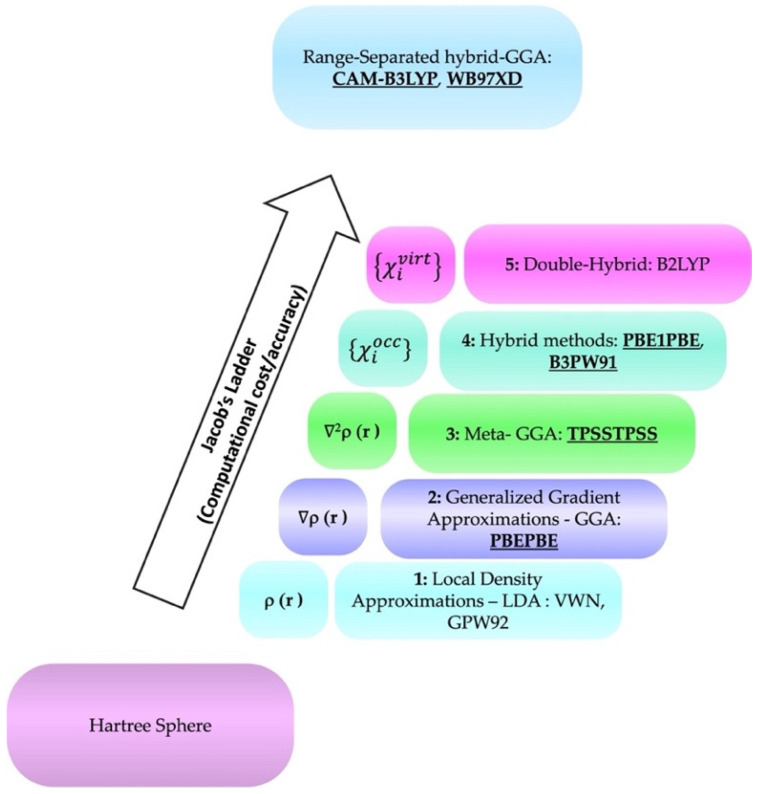
Jacob’s ladder organization of the functionals in increasing theoretical complexity. Hartree and range-separated-GGA functionals are not included in this classification approach (In bold: functionals used in this work) [10].

Recently, we developed an “easy-to-use” method that allowed us to directly predict the pKa of a series of substituted phenols and carboxylic acids with reliable results [1,8]. Briefly, our methodology requires the introduction of two explicit water molecules at the reaction center, CAM-B3LYP as functional, 6-311G+(d,p) as the basis set and SMD. Belonging to the RSH-GGA class of functionals [9], CAM-B3LYP exploits different parameters to describe long- or short-range interactions. In the long-range interaction, a Hartree–Fock contribution equal to 19% is utilized, while in the short one, it amounts to 65%. In both cases, the counterpart of the total is fulfilled with Becke 1988 (B88) exchange interaction [9]. Hence, CAM-B3LYP requires a higher computational cost with respect to hybrid-GGA or meta-GGA functionals, belonging to the third and fourth rungs of “Jacob’s ladder” (Figure 1) [10], which classifies density functionals according to exchange–correlation energy as a function of the electron density.

For instance, PBEPBE belongs to the second rung, the GGAs, where the non-local correlation and exchange are improved with respect to the spin electron density functionals family (first rung, LDSA) [10,11]. In the third rung, which includes meta-GGA functionals, as TPSSTPSS, the accuracy in treating chemical bonds and/or weak interactions is improved. In the fourth and fifth rungs, hybrid and meta-hybrid functionals exploit 20% of the HF exchange term [12]. In particular, the hybrids-GGAs are constructed on the Kohn–Sham orbitals, and they exploit the Laplacian of the spin density, avoiding non-locality. Nevertheless, this system is not more computationally expensive than the GGA ones [10]. Thus, PBE1PBE and B3PW91 are a revised version of the Kohn–Sham determinant, where angles, system-average exchange and orbitals nodality are partially or completely fixed [13]. CAM-B3LYP and WB97XD, belonging to the RSH-GGA family, are not included in “Jacob’s ladder” [14]. They combine an exact exchange term and an exact “partial correlation” term, maintaining a certain degree of approximation [10,14,15], thus boding good accuracy in a wide panel of applications.

Herein, the reliability of our “easy-to-use method” [1,8] for pKa determination was verified, by screening a panel of functionals belonging to different rungs of Jacob’s ladder. The main goal was to propose other functionals that show similar accuracy and reliability to CAM-B3LYP, with lower computational cost, eventually. Hence, PBEPBE, TPSSTPSS, PBE1PBE, B3PW91 and the “outcasts” CAM-B3LYP and WB97XD were exploited for the *in silico* pKa determination of a series of benzoic acids, functionalized with different substituents. No correction factors, mathematical fittings or experimental energy values were introduced. The 6-311G+(d,p) basis set and SMD were kept constant for each analysis. In addition, taking into consideration the pivotal role of the final geometry of the reaction center highlighted in the literature, a thorough analysis of the solvation cavities, bond lengths and dihedral angles was carried out. Computational time cost was also estimated for each molecule, to better clarify the performance of the exploited functionals.

## 2. Results and Discussion

To calculate the pKa of the selected benzoic acid derivatives, our previously optimized method was herein adopted [1,8]. In particular, the acid dissociation equilibrium of a generic carboxylic acid (RCO_2_H) and the corresponding reaction free energy (ΔG_dep_) can be defined according to Equations (1) and (2), respectively [8].

RCO_2_H(H_2_O)_2(sol)_ + OH^−^(H_2_O)_2(sol)_ ⇌ RCO_2_^−^(H_2_O)_2(sol)_ + H_2_O(H_2_O)_2(sol)_ (1)

ΔG_dep_ = G_RCO2_^−^ + G_H2O_ − G_OH_^−^ − G_RCO2H_ (2)

where G_RCO2_^−^, G_H2O_, G_OH_^−^ and G_RCO2H_ are the Gibbs free energies of each species in the presence of two explicit water molecules, in an aqueous solvent. Afterwards, the pKa equation, at 298.15 K, can be calculated according to Equation (3) [16]:

pK_a_ = ΔG_dep_/2.302RT + 15.74 (3)

where R is the gas constant, T is the absolute temperature, and 15.74 is the pKa of water at 298.15 K [17].

To calculate pKa values, PBEPBE was selected as functional due to its ability to predict non-covalent and weak interactions. It belongs to the second rung of “Jacob’s ladder”, showing a moderate computational cost and providing a fine consistency on a wide range of interactions [18]. TPSSTPSS well reproduces the geometry of small organic compounds bearing oxygen and/or hydrogen in functional groups [19]. Further, belonging to the third rung of Jacob’s ladder, it still requires reduced processing time. In the fourth rung, PBE1PBE and B3PW91 were selected to investigate how the HF contribution to exchange interactions affects the system description accuracy. For instance, PBE1PBE is based on PBEPBE, but it includes an HF contribution of 3:1 [20,21]. It was selected because of its reliability in describing the geometry of small compounds, as well as bond lengths, van der Waals interactions and hydrogen-bonded complexes, especially when a 6-311G+(d,p) basis set is exploited [21]. B3PW91 shows a non-local correlation function equal to 20%, producing stunning results in determining the H-bond length between oxygen belonging to medium/small organic compounds and water molecules [22]. CAM-B3LYP has already proven its accountability and reliability in the pKa determination of small organic molecules [1,8], while WB97XD is an RSH-GGA functional, widely used for *in silico* prediction of organic molecules properties. It usually ensures even greater reliability with respect to CAM-B3LYP, comprising 22% of HF exchange for short-range interactions and 100% HF for long-range interactions. Further, the two functionals differ in the intermediate region connecting short- and long-range interactions: while in CAM-B3LYP the standard error function, describing the range separation (ω), is approximately 0.33 [23], in WB97XD it is significantly smaller (ω = 0.2) [24].

The basis set, namely 6-311 G+(d,p), and the solvation model based on density, i.e., SMD, were kept constant in all the investigations [1,8].

Selected compounds are the following: Benzoic acid as the leading compound, 2,6-Dimethylbenzoic acid, where the pKa is affected by the “ortho-effect” of the alkyl substituents [8], 4-Cyanobenzoic acid, bearing a difficult-to-model electron-withdrawing group (EWG) as -CN, 2-Bromobenzoic and 4-Bromobenzoic acid, 2-, 3- and 4-Chlorobenzoic acids and 2-, 3- and 4-Methoxybenzoic acids, bearing a halogen atom or a methoxy group in different positions of the ring, thus implying different inductive and/or conjugative effects.

**Figure 2:**
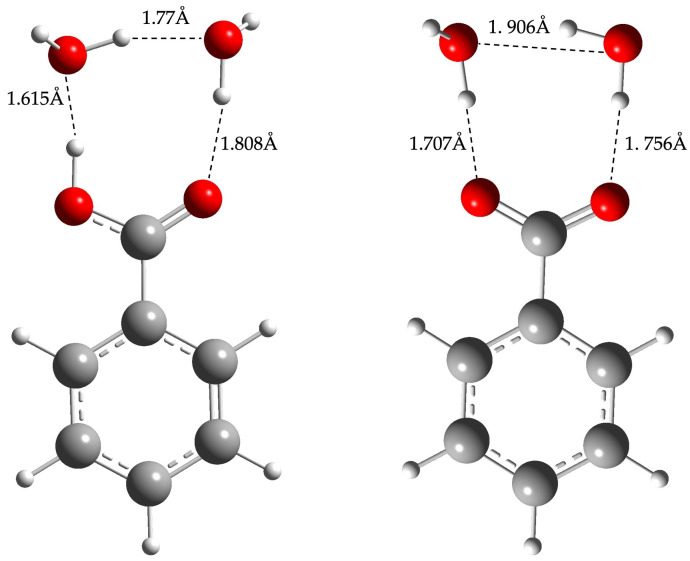
Optimized geometry of benzoic acid (**left**) and benzoate (**right**) in the presence of 2 explicit water molecules. Geometry optimization performed with a CAM-B3LYP functional, 6-311G+(d,p) basis set and SMD in water.

The initial geometry of benzoic acid and benzoate in the presence of 2 explicit water molecules was drawn as close as possible to the final ones, according to the literature [1,3,17]. Geometry optimization of the other target molecules was then performed, starting from the leading compounds and adding the appropriate substituent(s) on the ring. Hence, following our model, for each set of compounds (i.e., the acid and the conjugated base), two water molecules were placed at the reaction center [1,8], as shown in Figure 2.

After geometry optimization, the pKa values of the abovementioned molecules were calculated according to Equations (2) and (3). The results are listed in Table 1.

CAM-B3LYP confirmed its reliability in determining the pKa of carboxylic acids, with an MAE well below 1 unit (MAE = 0.23), which is the threshold limit for reliable methods [1,8]. WB97XD, despite belonging to the same class of functionals, showed an MAE value higher than 1 (MAE = 1.30). Due to the differences between the two functionals, namely the HF contribution to the long-range interactions and the ω value, this finding suggests that those parameters affect the WB97XD capability to correctly describe ionogenic reactions. Remarkably, B3PW91 and PBE1PBE show an MAE value slightly higher than the one obtained with CAM-B3LYP, even with a simpler level of theory (MAE = 0.38 for the former and MAE = 0.34 for the latter). The worst results were obtained with PBEPBE (MAE = 1.88), while TPSSTPSS showed a slightly lower MAE value (1.42).

Correlation plot analysis confirmed the overall reliability of CAM-B3LYP with a slope close to the theoretical one (slope = 1.05 ± 0.02), a Pearson’s r value (R) and R-Square (COD) of 0.99, (Figure S1, Supplementary Information). B3PW91 showed a COD of 0.97 and a slope value nearly coincident with the unit, while PBE1PBE was less accurate (slope = 0.94), even with a lower MAE. *En masse*, the results suggested that these two functionals marginally underestimated pKas, in a systemic manner (R > 0.99 and COD > 0.98 in both cases). Functionals belonging to rungs 2 and 3 (Figure 1, namely PBEPBE and TPSSTPSS) pointed out overestimated pKa values, leading to slopes higher than 1 unit (slope_PBEPBE_ = 1.51 and slope_TPSSTPSS_ = 1.14, respectively). Further, while PBEPBE R and COD values suggested a systematic internal error (R > 0.99 and COD > 0.99), TPSSTPSS showed less accuracy in predicting pKas (R > 0.93 and COD > 0.87).

A more detailed analysis identified that CAM-B3LYP well predicted the pKa values of bromo- and chloro-substituted benzoic acids, with negligible ΔpKa, while a slightly lower precision in predicting the pKa of compounds affected by the “ortho” effect was obtained. Indeed, the major discrepancy in ΔpKa was in the case of 2,6-Dimethylbenzoic acid (Table S1, Supplementary Information). Conversely, B3PW91 well depicted such an effect, showing the lowest ΔpKa among the screened functionals. It also well predicted the pKa of 3- and 4-Chlorobenzoic acids, as well as methoxy-substituted benzoic acids. Conversely, unsatisfactory results were obtained when -Br and -CN were considered, partially in agreement with what was reported in the literature [1]. To note, PBE1PBE proved to be remarkable in predicting the same compounds, with a trivial discrepancy.

**Figure 3:**
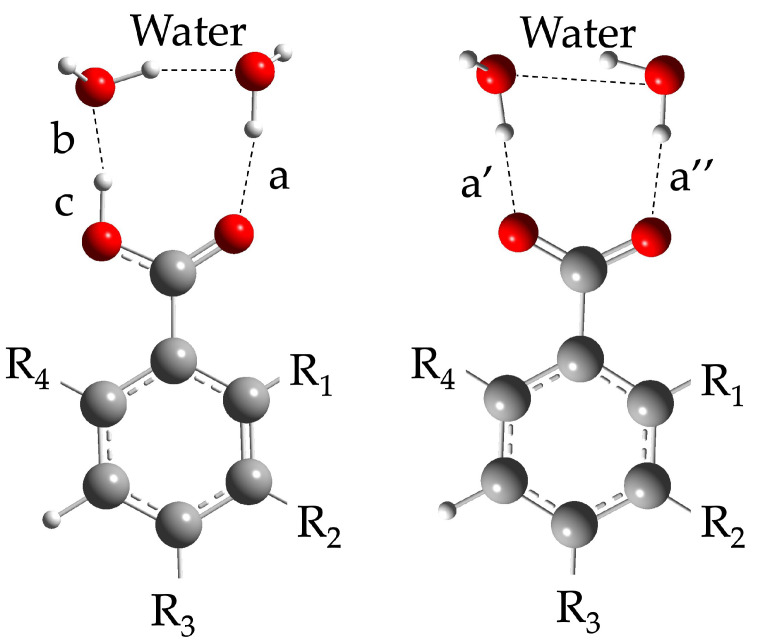
Schematic representation of parameters considered in the bond length analysis for the acid-conjugated base pair, calculated for each functional. R_1_: bromo for 2-Bromobenzoic acid and 2-Bromobenzoate; chloro for 2-Chlorobenzoic acid and 2-Chlorobenzoate; methoxy for 2-Methoxybenzoic acid and 2-Methoxybenzoate. R_1_ and R_4_: methyl for 2,6-Dimethylbenzoic acid and its conjugated base. R_2_: chloro in 3-Chlorobenzoic acid and its conjugated base; methoxy in 3-Methoxybenzoic acid and its conjugated base; R_3_: bromo in 4-Bromobenzoic acid and its conjugated base; cyano for 4-Cyanobenzoic acid and its conjugated base; chloro in 4-Chlorobenzoic acid and its conjugated base; methoxy in 4-Methoxybenzoic acid and its conjugated base. When not specified, substituents must be considered as hydrogen. Geometry optimization, with all functionals, was performed with a 6-311G+(d,p) basis set and SMD as the solvation model. **a** distance between carbonyl O and H of a water molecule, **b** distance between **O**H_2_ and **H** of the acidic group, **c** distance H-O in acid group, **a’** and **a”** distances of the hydrogens of the 2 water molecules and oxygens of the carboxylate group.

Realistic modeling of the shape of the solvation cavity is a major goal in obtaining consistent computed pKa values [8]. Indeed, it was previously demonstrated that coarse models of the first solvation shell could cause huge inaccuracy in determining pKa [5]. Further, several attempts were made in re-shaping the “reaction centre” with encouraging results [1,2,3,4,5,8]. Therefore, to better understand if the difference in pKa values obtained with different functionals could be related with a significant modification of the solvation cavity, an extensive analysis was performed. However, no remarkable discrepancy among CAM-B3LYP and the other functionals was observed in the reaction center geometry, nor in the shape of the overall solvation cavity around the molecules (Figure S2, Supplementary Information). Thus, a punctual estimation of each species involved in the reaction center was performed, through the bond length determination and dihedral evaluation. Due to the trustworthy MAE value [1,8], the bond lengths returned by CAM-B3LYP were considered as the standard ones, assuming its ability to mimic the solvation cage geometry similar to the “real” one. Thus, the differences were referred to its bond values (thereafter Δ_length_). For each acid species, the distance among the moieties describing the reaction center was considered, as reported in Figure 3.

**Figure 4:**
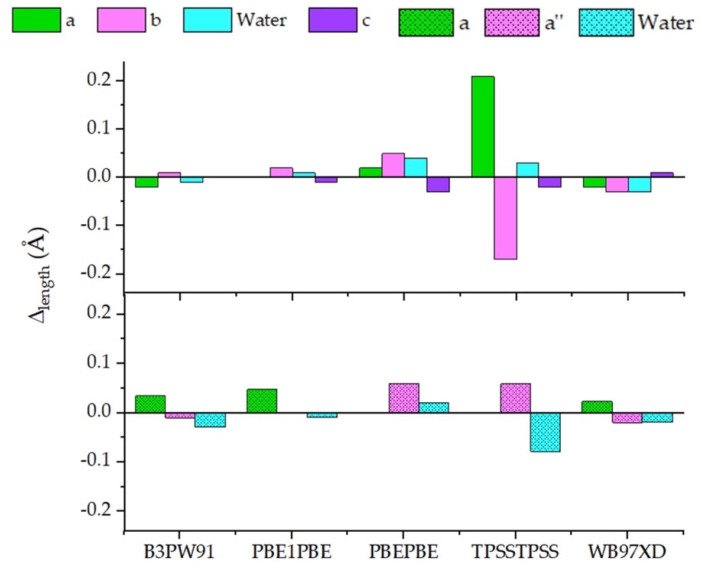
Benzoic acid bond length differences (Δ_length_) calculated among CAM-B3LYP, taken as reference, and the different functionals. Δ_length_ computed for the acid species (**top**) and the conjugated base (**bottom**).

As shown in Figure 4 (and Figure S3, Supplementary Information), Δ_length_ values with respect to the value provided by CAM-B3LYP kept as reference were always below 0.25 Å. Indeed, for each molecule, bond length analysis led to consistent values with all the functionals (Figure S3, Supplementary Information). The major differences have been detected for benzoic acid with TPSSTPSS (Figure 4), which actually led to ΔpKa > 1 (Table 2).

Overall, in our computational settings, B3PW91 reported more similar bond lengths compared to the CAM-B3LYP ones. Remarkably, this functional showed the highest discrepancy (0.07 Å) in predicting the *c* length of 4-Cyanobenzoic acid, although returning an accurate pKa value. This behavior was shared with the other functionals, where the same reliability in pKa determination was not pursued. Similarly, no significant changes with respect to CAM-B3LYP emerged in the bond length analysis with PBE1PBE. Further, in the conjugated bases, the bond length discrepancy was always below 0.02 Å, even when the 2-Chlorobenzoic acid was considered. PBEPBE, together with TPSSTPSS, and WB97XD showed the most scattered values, and the larger gap was approximately 0.06 Å by PBEPBE in describing 2-Chlorobenzoate and 2-Methoxybenzoate. Unlike what was observed for other systems, where even minimal variations in bond length were significative [22,24], in our conditions not a clear correlation between Δ_length_ and calculated pKa emerged.

Thus, dihedral angles in the acid and conjugated bases were also analyzed (Table S2, Supplementary Information). The results showed that the twist angle formed by the carboxylic/carboxylate moiety and the aromatic ring marginally affected the reliability in pKa determination. For instance, in benzoic acid, the dihedral angle was in CAM-B3LYP, PBE1PBE and PBEPBE, despite the ΔpKa varying up to 1.87 units. In 2-Bromobenzoic acid, a change of only two degrees was related with a pKa variation up to 1.5, as shown in PBEPBE. Similar outcomes were obtained comparing 4-Metoxybenzoic acid: PBE1PBE and WB97XD returned similar dihedral values, although substantial differences in pKas were observed. Even for the conjugated bases, the differences in the dihedral angles were blurred. For instance, 3-Chlorobenzoate, 4-Chlorobenzoate and 2-Methoxybenzoate showed similar dihedral values in contempt of massive ΔpKa variations, with PBEPBE and TPSSTPSS. Since functionals include self-correction factors that might shield meaningful dihedral differences between the acid and the conjugated base, the average twist occurring between the carboxylic moiety and the aromatic one was considered. Table 2 summarizes the average value in the angles formed by the carboxylic group with respect to the aromatic ring, in the acids and conjugated bases.

Overall, such data suggested no strict correlation between the geometry organization of the solvation cage and the reliability in returning pKa values. Therefore, a large contribution of the self-correction error of the functional itself, coupled with the adopted solvation model, can be anticipated.

For each functional, the computational cost (CC) was defined as the average time, in minutes, for computing the acid and the conjugated base of all compounds. Specifically, the number of shared processors and the memory (CPU) limit were kept constant for each functional. Thus, in the present work, values obtained with CAM-B3LYP belong to a new set of optimizations, confirming previous massive results [7].

It should be noted that WB97XD and TPSSTPSS showed a considerable computational cost (49 and 51 min, respectively) (Figure S4, Supplementary Information). PBEPBE, PBE1PBE and B3PW91 implied a machine working time in the range of half an hour, while CAM-B3LYP required the highest computational cost, equal to 52 min, which is compensated for by its high accuracy in predicting pKas. Interestingly, B3PW91 and PBE1PBE pointed out very interesting results in terms of the machine-costs/reliability ratio, since they led to slightly less accurate results than CAM-B3LYP, but in shorter processing time.

## 3. Materials and Methods

### Computational Method and Data Analysis

DFT calculations have been carried out using Gaussian 16 rev. A. 03 [26]. Geometry and frequency optimizations have been performed in vacuum and in continuum solvent for all compounds. The true minima (no imaginary frequencies) were selected for each species. Computations were carried out applying CAM-B3LYP, B3PW91, PBE1PBE, PBEPBE, TPSSTPSS and WB97XD as functionals, with a 6-311+G(d,p) basis set. The water environment was simulated by exploiting the SMD as a continuum solvation model. For each functional, Gibbs free energy has been collected in the presence of two explicit water molecules. The two water molecules were oriented at the reaction center [2], foreseeing the putative low-energy structure in the transition state, and drawn using GaussView 6.0 software. pKa calculations were carried out at 298.15 K for both gaseous and aqueous solution phases. Bond lengths were calculated through GaussView 6.0 as the distance from the center of one atomic bead (representing hydrogen, oxygen, carbon) to the other one. The dihedral angle was determined as the rotation of the carboxyl moiety of the different compounds and the planar aromatic ring. Processing time was considered as the time, reported by the Gaussian result table, expressed in minutes. The average time was considered as the sum of the time needed to compute the acid/base conjugated pair. Calculations were performed with an Intel Xeon CPU E5-2660 and 32 GB RAM, using 20 processors and 20 GB RAM. pKa_ref_ values were obtained from the NIH (National Library of Medicine) through PubChem [25].

## 4. Conclusions

In this work, we have compared different functionals for the direct pKa determination of a series of carboxylic acids using a user-friendly protocol previously optimized [8]. Several DFT functionals, characterized by different levels of HF exchange, fixed parameters and modeling of short- and long-range interactions, were considered. Among all, CAM-B3LYP confirmed its reliability, producing the best results in pKa computational determination, with a very low MAE value (0.23), and an average computational cost in terms of processing time of 52 min. The hybrid-GGA functional B3PW91 well predicted pKas, with an MAE slightly below 0.4 and a computational cost equal to 35 min. The MAE value of the analog PBE1PBE was similar to the former one (MAE = 0.34). Conversely, the worst results were obtained with WB97XD (MAE = 1.30, CC = 51 min), TPSSTPSS (MAE = 1.42, CC = 49 min) and PBEPBE (MAE = 1.88, CC = 31 min). To investigate the causes of these output differences, solvation cavity, bond lengths and dihedral angles analyses were performed. Unlike what is reported in the literature, no remarkable differences in the structural features of the solvation cavities and in bond lengths were highlighted by the analysis of the geometry of the reaction center with the different functionals. The only meaningful change, above 0.2 Å, was obtained with benzoic acid.

Noteworthy, the data suggested that the geometry of the reaction center had only a marginal role in predicting pKas. Therefore, a major role is likely played by the internal correction of the functionals and the adopted solvation model.

In conclusion, the present study pointed out that, next to CAM-B3LYP, other functionals can be exploited for calculating pKas, through a “user-friendly” methodology, with appreciable reliability. Specifically, B3PW91 and PBE1PBE showed lower accuracy than CAM-B3LYP, although their MAE was lower than 0.4, but a significant advantage in terms of computational time cost.

## Figures and Tables

**Table 1:** Calculated pKa values with different functionals for carboxylic acid derivatives. Geometry was optimized with a 6-311G+(d,p) basis set and SMD as the solvation model in water. pKa_ref_ values were obtained from the NIH (National Library of Medicine) through PubChem [25].

COMPOUND	pKa_ref_	CAM-B3LYP	B3PW91	PBE1PBE	PBEPBE	TPSSTPSS	WB97XD
Benzoic Acid	**4.20**	4.35	3.83	3.54	6.07	5.22	2.94
4-Cyanobenzoic Acid	**3.55**	3.32	2.96	2.68	4.93	4.22	1.92
2,6-Dimethylbenzoic Acid	**3.24**	3.89	3.39	3.57	0.35.22	4.81	2.04
4-Bromobenzoic Acid	**3.96**	4.00	3.27	3.65	5.94	4.77	2.74
2-Bromobenzoic Acid	**2.96**	3.02	3.86	2.33	4.87	4.18	1.62
2-Chlorobenzoic Acid	**2.96**	3.24	2.36	2.36	3.96	3.23	0.78
3-Chlorobenzoic Acid	**3.83**	3.91	3.62	3.67	5.91	5.03	2.59
4-Chlorobenzoic Acid	**3.99**	3.98	3.77	3.91	6.20	5.30	2.70
2-Methoxybenzoic Acid	**4.09**	4.46	4.20	4.28	6.17	5.93	2.83
3-Methoxybenzoic Acid	**4.10**	4.54	3.99	4.00	6.21	5.37	2.93
4-Methoxybenzoic Acid	**4.50**	4.74	4.76	4.74	7.02	0.20	3.62
MAE		0.23	0.35	0.34	1.88	1.42	1.30

**Table 2:** Average dihedral changing for aromatic carboxylic acid derivatives pairs (acid and conjugated base) with a 6-311G+(d,p) basis set and SMD as the solvation model.

COMPOUND	CAM-B3LYP	B3PW91	PBE1PBE	PBEPBE	TPSSTPSS	WB97XD
Benzoic Acid	0.32	0.52	0.36	0.44	0.39	0.72
4-Cyanobenzoic Acid	0.67	0.52	0.83	1.12	0.90	0.99
2,6-Dimethylbenzoic Acid	67.03	68.90	66.86	69.07	63.76	69.80
4-Bromobenzoic Acid	0.41	0.56	0.95	0.70	1.15	0.67
2-Bromobenzoic Acid	68.30	69.38	67.55	69.80	67.36	70.81
2-Chlorobenzoic Acid	68.97	67.085	66.09	66.215	63.82	70.30
3-Chlorobenzoic Acid	0.49	0.42	0.31	0.36	0.28	0.50
4-Chlorobenzoic Acid	0.47	0.63	0.57	0.73	0.70	0.65
2-Methoxybenzoic Acid	35.76	37.56	33.64	36.30	31.91	43.16
3-Methoxybenzoic Acid	0.29	0.02	0.23	0.20	0.06	0.48
4-Methoxybenzoic Acid	0.28	0.30	0.23	0.40	0.20	0.78

## Data Availability

Data are contained within the article and Supplementary Materials.

## Supplementary Materials

The following supporting information can be downloaded at: https://www.mdpi.com/article/10.3390/molecules29061255/s1.
