# Supplementary material for: Towards the “Eldorado” of pKa Determination: A Reliable and Rapid DFT Model
Source: Molecules. 2024 Mar 12;29(6):1255. doi: 10.3390/molecules29061255 (PMC10974196; doi:10.3390/molecules29061255)
Supplement: Supplementary file 1 [file molecules-29-01255-s001.zip › molecules-2873960-supplementary.pdf]

Supporting Information

Contents

Figure 1	S1
Table 1	S1
Figure 2	S2
Figure 3	S3
Table 2	S3
Figure 4	S4
Optimized cartesian coordinates (in Angstroms)	S4

Equation	y = a + b*x					
Plot	pKacal	pKacal	pKacalc	pKacal	pKacal	pKacal
Intercept	0 ± --					
Slope	1.04894 ± 0.01942	0.96513 ± 0.03681	0.94224 ± 0.03336	1.51102 ± 0.02365	1.14258 ± 0.1367 3	0.65877 ± 0.03744
Pearson's r	0.99829	0.9928	0.99379	0.99878	0.93527	0.98423
R-Square (COD)	0.99659	0.98566	0.98762	0.99756	0.87474	0.96871
Adj. R-Square	0.99624	0.98423	0.98638	0.99731	0.86221	0.96558

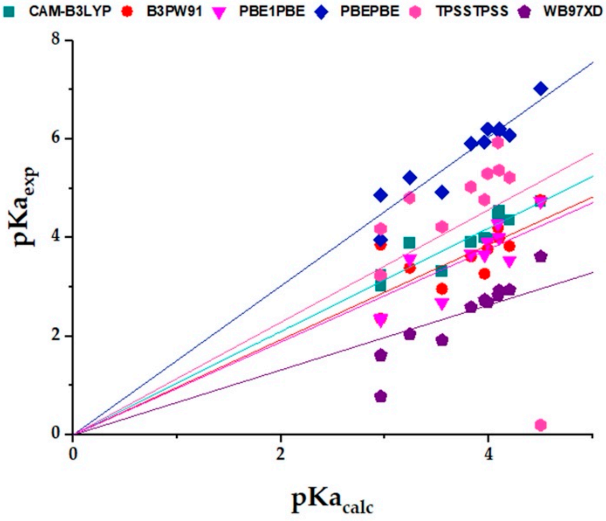

Figure S1. Calculated pKa vs. experimental pKa plot. pKa_{calc} obtained with different functionals: blue square: CAM-B3LYP, red circle: B3PW91, green triangle: PBE1PBE, magenta triangle: PBEPBE, blue diamond: TPSSTPSS, purple triangle: WB97XD, with 6-311G+(d,p) and SMD as continuum model.

Table S1. Calculated ΔpKa with different functionals for carboxylic acid derivatives. Geometry was optimized with 6-311G+(d,p) basis set and SMD as solvation models, in water.

COMPOUND	pKa_{ref}	ΔpKa CAM-B3LYP	ΔpKa B3PW91	ΔpKa PBE1PBE	ΔpKa PBEPBE	ΔpKa TPSSTPSS	ΔpKa WB97XD
Benzoic Acid	**4.20**	0.15	0.38	0.66	1.87	1.02	1.26
4-Cyanobenzoic Acid	**3.55**	-0.23	-0.59	-0.87	1.38	0.67	-1.63
2,6-Dimethylbenzoic Acid	**3.24**	0.65	0.15	0.33	1.98	1.57	-1.20
4-Bromobenzoic Acid	**3.96**	0.04	-0.69	-0.31	1.98	0.81	-1.22
2-Bromobenzoic Acid	**2.96**	0.06	0.90	0.14	1.50	1.38	-0.94
2-Chlorobenzoic Acid	**2.96**	0.28	-0.60	-0.60	1.00	0.27	-2.18
3-Chlorobenzoic Acid	**3.83**	0.08	-0.21	-0.16	2.08	1.20	-1.24
4-Chlorobenzoic Acid	**3.99**	-0.01	-0.22	-0.08	2.21	1.31	-1.29
2-Metoxybenzoic Acid	**4.09**	0.37	0.11	0.19	2.08	1.84	-1.26
3-Metoxybenzoic Acid	**4.1**	0.44	-0.11	-0.10	2.11	1.27	-1.17
4-Metoxybenzoic Acid	**4.5**	0.24	0.26	0.24	2.52	-4.30	-0.88
MAE		0.23	0.35	0.34	1.88	1.42	1.3

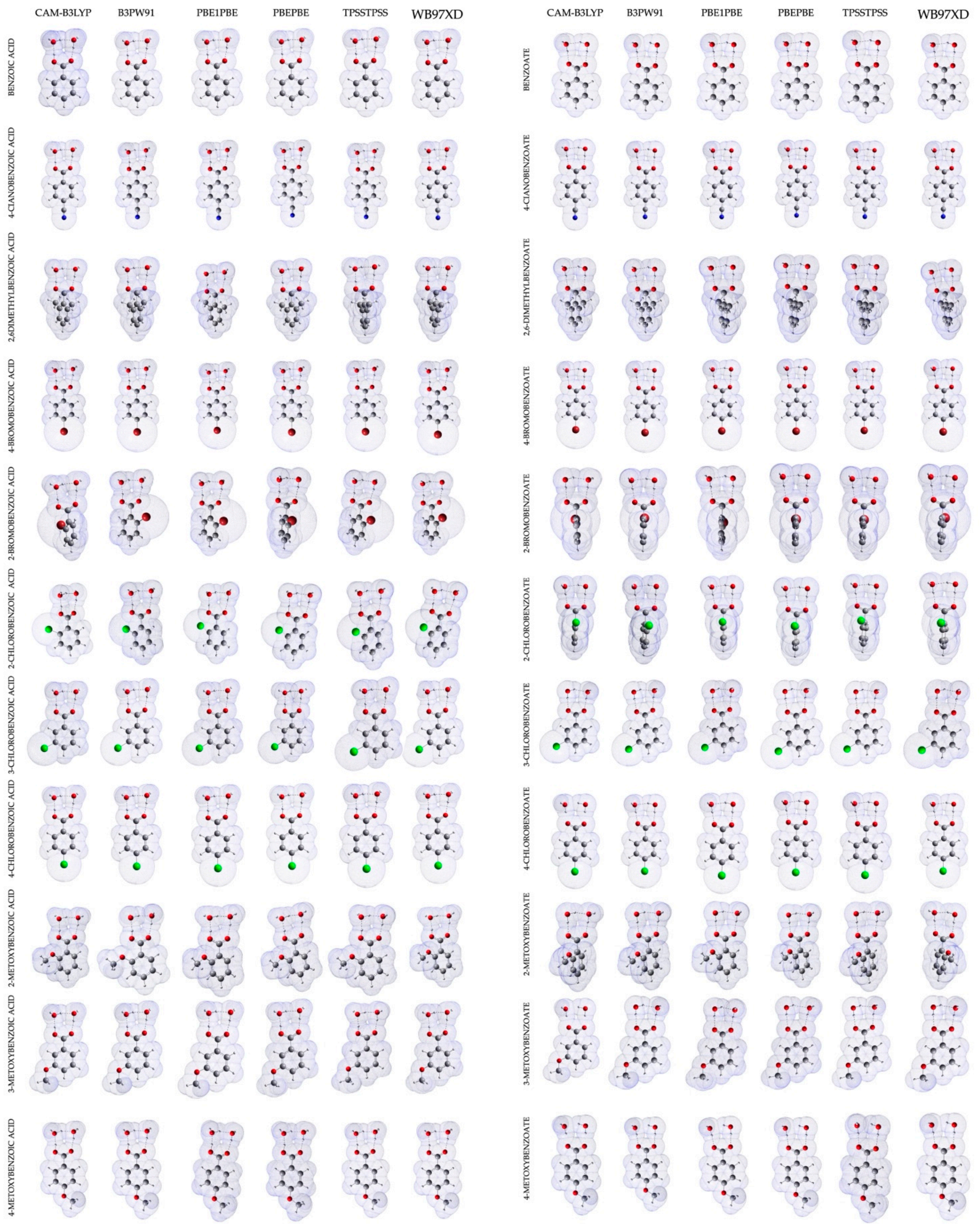

Figure S2. Solvation cavities with two explicit water molecules. Column listed the different functional exploited while rows list the referring compounds. Geometry optimization performed with 6-311+G(d,p) as basis set, SMD as solvation model.

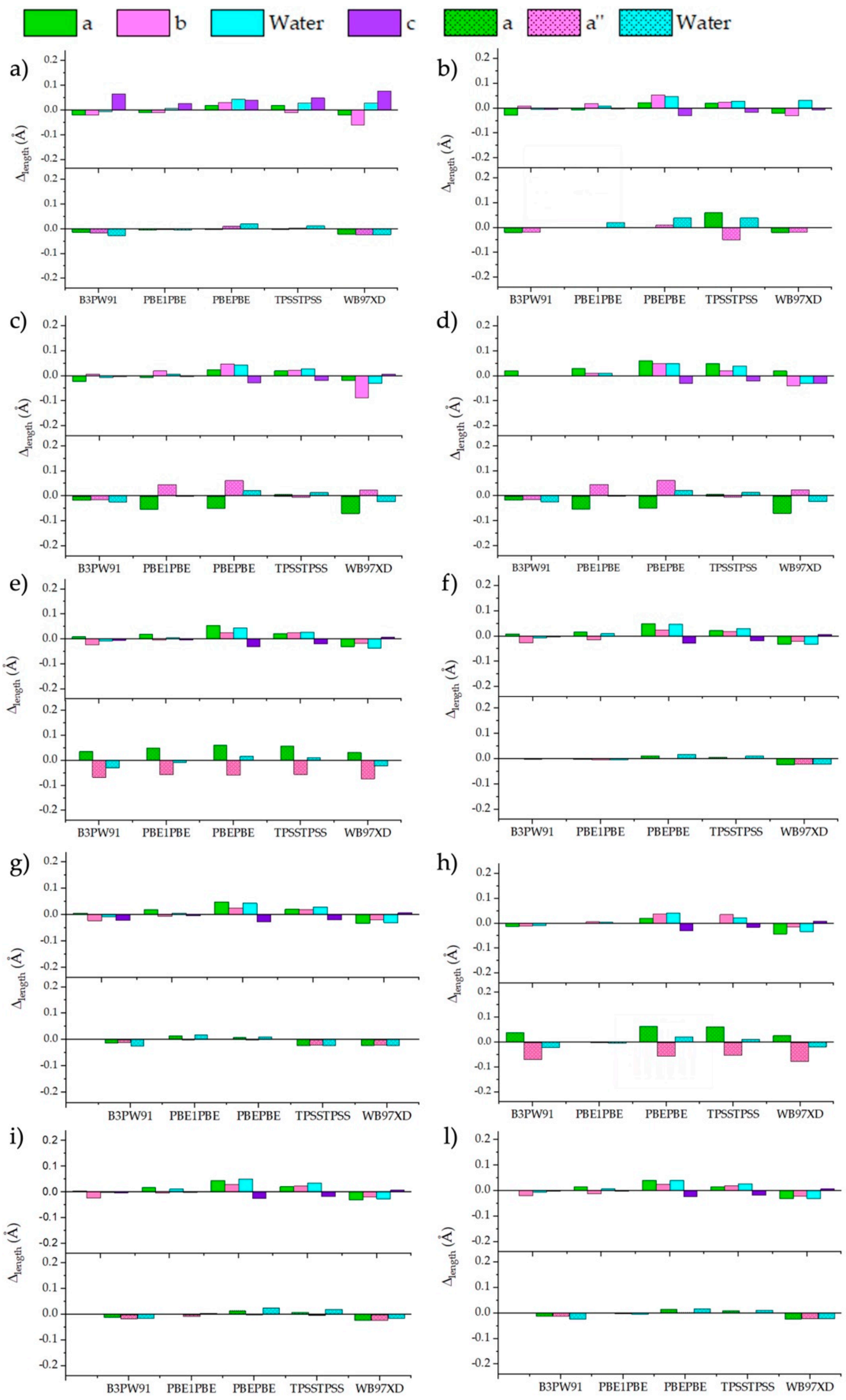

Figure S3. Bond length differences (Δ_{length}) calculated among CAM-B3LYP, taken as reference, and the different functionals. **a:** 4-Cyanobenzoic Acid; **b:** 2,6 Dimethylbenzoic Acid, **c:** 4-Bromobenzoic Acid, **d:** 2-Bromobenzoic Acid, **e:** 2-Chlorobenzoic Acid, **f:** 3-Chlorobenzoic Acid, **g:** 4-Chlorobenzoic Acid, **h:** 2-Metoxybenzoic Acid, **i:** 3-Metoxybenzoic Acid, **l:** 4-Metoxybenzoic Acid. Δ_{length} computed for the acid species (top) and the conjugated base (bottom), taking the bond length of CAM-B3LYP as refence.

Table S2. Calculated dihedral for aromatic carboxylic acid derivatives with 6-311G+(d,p) basis set and SMD, as solvation model.

COMPOUND	CAM-B3LYP	B3PW91	PBE1PBE	PBEPBE	TPSSTPSS	WB97XD
Benzoic Acid	0.545	0.604	0.528	0.563	0.458	0.785
Benzoate	0.089	0.4283	0.199	0.323	0.326	0.654
4-Cyanobenzoic Acid	1.084	1.194	1.106	1.317	1.004	1.493
4-Cyanobenzoate	0.249	0.844	0.563	0.915	0.777	0.486
2,6-Dimethylbenzoic Acid	60.255	62.000	59.938	61.531	56.833	63.606
2,6-Dimethylbenzoate	73.823	75.781	73.774	76.612	71.198	75.999
4-Bromobenzoic Acid	0.757	0.8155	1.787	0.8523	1.787	0.911
4-Bromobenzoate	0.065	0.300	0.107	0.556	0.506	0.485
2-Bromobenzoic Acid	46.484	47.628	44.263	47.975	42.92	52.661
2-Bromobenzoate	90.115	91.138	90.840	91.633	91.799	88.957
2-Chlorobenzoic Acid	45.25	43.37	42.58	40.80	39.92	49.88
2-Chlorobenzoate	92.69	90.80	89.6	91.63	87.72	90.73
3-Chlorobenzoic Acid	0.11	0.01	0.02	0.26	0.01	0.36
3-Chlorobenzoate	0.86	0.83	0.6	0.46	0.54	0.63
4-Chlorobenzoic Acid	0.79	0.80	0.82	0.81	0.76	1.00
4-Chlorobenzoate	0.16	0.46	0.33	0.66	0.64	0.30
2-Metoxybenzoic Acid	23.12	26.70	22.86	22.94	18.79	34.40
2-Metoxybenzoate	48.40	48.43	44.43	49.66	45.04	51.93
3-Metoxybenzoic Acid	0.56	0.018	0.00	0.26	0.10	0.18
3-Metoxybenzoate	0.012	0.018	0.453	0.13	0.013	0.78
4-Metoxybenzoic Acid	0.50	0.54	0.45	0.51	0.37	0.45
4-Metoxybenzoate	0.07	0.06	0.013	0.28	0.034	1.11

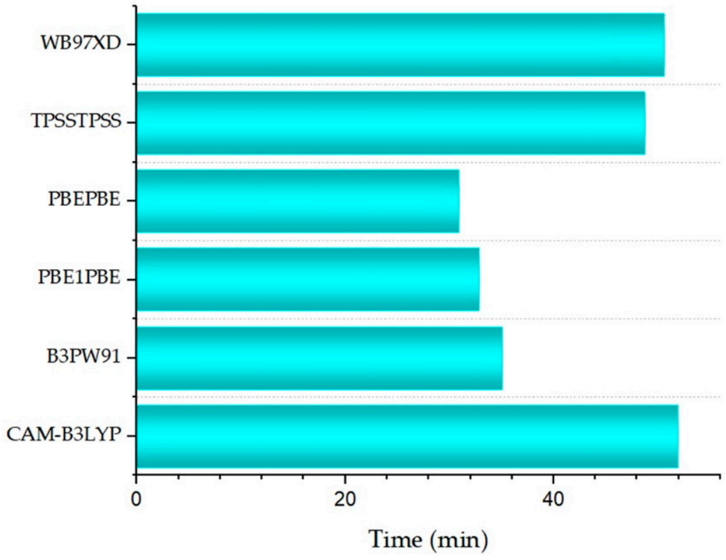

Figure S4. Average time computational cost (CC) for the pKa determination of the pair acid-conjugated base, calculated for each functional. Geometry optimization, with all functionals, was performed 6-311G+dp basis set and SMD as solvation model.

Optimized cartesian coordinates (in Angstroms)

- **CAM-B3LYP 6-311G+(d,p) SMD**

H_2O

Electronic Energy -229.305236
Free Energy -229.305236
Stoichiometry H6O3
Framework group C1[X(H6O3)]
Deg. of freedom 21
Full point group C1 NOp 1
Largest Abelian subgroup C1 NOp 1
Largest concise Abelian subgroup C1 NOp 1
Standard orientation:

Center Number	Atomic Number	Atomic Type	Coordinates (Angstroms) X	Y	Z
1	8	0	0.000021	0.947227	-0.000152
2	1	0	0.000688	1.552306	-0.753130
3	1	0	-0.001525	1.516347	0.780298
4	8	0	2.416395	-0.484898	-0.003039
5	1	0	2.197483	-1.422680	0.015751
6	1	0	1.557614	-0.021044	-0.003576
7	8	0	-2.416336	-0.484958	-0.002152
8	1	0	-2.197316	-1.422847	0.007630
9	1	0	-1.557581	-0.021059	-0.004228

Rotational constants (GHZ): 15.8495915 2.4945704 2.177363s

OH^-

Electronic Energy -228.866593
Free Energy -228.840867
Stoichiometry H5O3(1-)
Framework group C1[X(H5O3)]
Deg. of freedom 18
Full point group C1 NOp 1
Largest Abelian subgroup C1 NOp 1
Largest concise Abelian subgroup C1 NOp 1
Standard orientation:

Center Number	Atomic Number	Atomic Type	Coordinates (Angstroms) X	Y	Z
1	8	0	-0.000225	0.657416	0.000044
2	1	0	-0.000769	1.616660	-0.000144
3	8	0	-2.351148	-0.340430	0.086619
4	1	0	-2.355030	-0.801681	-0.754038
5	1	0	-1.410034	0.086466	0.103715
6	8	0	2.351414	-0.340291	-0.086624
7	1	0	1.410559	0.086532	-0.103892
8	1	0	2.354945	-0.801536	0.754044

Rotational constants (GHZ): 31.7538037 2.6124257 2.4454439

Benzoic Acid

Electronic Energy -573.662609
Free Energy -573.537770
Stoichiometry C7H10O4
Framework group C1[X(C7H10O4)]
Deg. of freedom 57
Full point group C1 NOp 1
Largest Abelian subgroup C1 NOp 1
Largest concise Abelian subgroup C1 NOp 1
Standard orientation:

Center Number	Atomic Number	Atomic Type	Coordinates (Angstroms) X	Y	Z
1	6	0	3.743423	0.080612	0.014324

2	6	0	3.086559	-1.144061	0.019978
3	6	0	1.700545	-1.189328	0.008933
4	6	0	0.966360	-0.003997	-0.006712
5	6	0	1.628592	1.222658	-0.011891
6	6	0	3.013771	1.263863	-0.001815
7	1	0	4.826474	0.113291	0.022530
8	1	0	3.655307	-2.065709	0.032718
9	1	0	1.189035	-2.142282	0.012988
10	1	0	1.053321	2.139277	-0.024092
11	1	0	3.525600	2.218349	-0.006536
12	6	0	-0.518294	-0.015319	-0.017699
13	8	0	-1.182698	1.013502	-0.021309
14	8	0	-1.053520	-1.219724	-0.023770
15	1	0	-2.061300	-1.197844	-0.036859
16	8	0	-3.669474	-1.328631	-0.104872
17	1	0	-3.965547	-0.396571	-0.006885
18	1	0	-4.029571	-1.804967	0.652831
19	8	0	-3.932899	1.367767	0.138277
20	1	0	-4.251421	1.843445	-0.637575
21	1	0	-2.958905	1.343127	0.053568

Rotational constants (GHZ): 2.5491722 0.4627353 0.3924514

Benzoate

Electronic Energy -573.208713
Free Energy -573.098096
Stoichiometry C7H9O4(1-)
Framework group C1[X(C7H9O4)]
Deg. of freedom 54
Full point group C1 NOp 1
Largest Abelian subgroup C1 NOp 1
Largest concise Abelian subgroup C1 NOp 1
Standard orientation:

Center Number	Atomic Number	Atomic Type	Coordinates (Angstroms) X	Y	Z
1	6	0	-3.719496	0.076534	-0.008068
2	6	0	-2.979884	1.253168	-0.007470
3	6	0	-1.592709	1.202557	-0.000598
4	6	0	-0.929651	-0.023323	0.005698
5	6	0	-1.678813	-1.198615	0.005212
6	6	0	-3.066000	-1.150033	-0.001701
7	1	0	-4.802518	0.115391	-0.013660
8	1	0	-3.484760	2.211947	-0.012584
9	1	0	-1.016862	2.118720	-0.000525
10	1	0	-1.169032	-2.153156	0.009928
11	1	0	-3.638227	-2.070211	-0.002241
12	6	0	0.580768	-0.078705	0.011424
13	8	0	1.124819	-1.213600	0.014927
14	8	0	1.203430	1.017865	0.011841
15	8	0	3.854563	-1.438046	-0.005909
16	1	0	2.871850	-1.390776	0.004522
17	1	0	4.109377	-0.498917	-0.021213
18	8	0	3.871280	1.390853	-0.102142
19	1	0	2.894013	1.249426	-0.036448
20	1	0	4.118130	1.871496	0.695522

Rotational constants (GHZ): 2.5270193 0.4646701 0.3928940

4-Cyanobenzoic Acid

Electronic Energy -665.889952
Free Energy -665.769309
Stoichiometry C8H9NO4
Framework group C1[X(C8H9NO4)]
Deg. of freedom 60
Full point group C1 NOp 1
Largest Abelian subgroup C1 NOp 1
Largest concise Abelian subgroup C1 NOp 1
Standard orientation:

Center Number	Atomic Number	Atomic Type	X	Y	Z
1	6	0	3.000007	0.030087	0.004560
2	6	0	2.335742	-1.195973	0.016469
3	6	0	0.952543	-1.218312	0.007986
4	6	0	0.236034	-0.023954	-0.010778
5	6	0	0.906889	1.196310	-0.021812
6	6	0	2.288809	1.229939	-0.014794
7	1	0	2.900966	-2.118458	0.031672
8	1	0	0.431352	-2.165236	0.016646
9	1	0	0.342204	2.118652	-0.036210
10	1	0	2.818247	2.173493	-0.023889
11	6	0	-1.254966	-0.018336	-0.018548
12	8	0	-1.898366	1.021101	-0.014598
13	8	0	-1.797163	-1.215088	-0.030457
14	1	0	-2.808682	-1.189643	-0.039874
15	8	0	-4.398960	-1.302670	-0.102311
16	1	0	-4.692022	-0.369444	-0.002874
17	1	0	-4.761038	-1.779888	0.654001
18	8	0	-4.656343	1.391795	0.145503
19	1	0	-4.979713	1.871809	-0.625707
20	1	0	-3.683375	1.371472	0.056983
21	6	0	4.430329	0.058780	0.012426
22	7	0	5.580913	0.082129	0.018727

Coordinates (Angstroms)

Rotational constants (GHZ): 2.5398822 0.2772829 0.2503261

4-Cyanobenzoate
Electronic Energy -665,438211
Free Energy -665.331897
Stoichiometry C8H8NO4(1-)
Framework group C1[X(C8H8NO4)]
Deg. of freedom 57
Full point group C1 NOp 1
Largest Abelian subgroup C1 NOp 1
Largest concise Abelian subgroup C1 NOp 1
Standard orientation:

Center Number	Atomic Number	Atomic Type	X	Y	Z
1	6	0	3.000511	-0.003611	-0.000659
2	6	0	2.283426	-1.209869	-0.066660
3	6	0	0.894422	-1.187699	-0.066143
4	6	0	0.196052	0.022380	-0.001106
5	6	0	0.916764	1.219330	0.062927
6	6	0	2.305726	1.215868	0.064207
7	1	0	2.822907	-2.148863	-0.117649
8	1	0	0.315836	-2.101994	-0.116518
9	1	0	0.354204	2.143883	0.110850
10	1	0	2.862337	2.144821	0.114345
11	6	0	-1.342486	0.042071	-0.000024
12	8	0	-1.875392	1.170279	0.050653
13	8	0	-1.893079	-1.090661	-0.049038
14	8	0	-4.739548	1.481420	-0.127760
15	1	0	-3.764675	1.490368	-0.064241
16	1	0	-4.926961	0.530093	-0.159539
17	8	0	-4.568925	-1.478385	0.042441
18	1	0	-3.590819	-1.325879	-0.052199
19	1	0	-4.677427	-1.678920	0.976111
20	6	0	4.429985	-0.017268	0.000842
21	7	0	5.587680	-0.028850	0.002596

Coordinates (Angstroms)

Rotational constants (GHZ): 2.4272317 0.2736266 0.2462903

2,6-Dimethylbenzoic Acid
Electronic Energy -652,259017
Free Energy -652.082846
Stoichiometry C9H14O4
Framework group C1[X(C9H14O4)]

Deg. of freedom 75
Full point group C1 NOp 1
Largest Abelian subgroup C1 NOp 1
Largest concise Abelian subgroup C1 NOp 1
Standard orientation:

Center Number	Atomic Number	Atomic Type	Coordinates (Angstroms) X	Y	Z
1	6	0	-3.601314	0.109154	0.047875
2	6	0	-2.952020	-1.097216	0.246430
3	6	0	-1.563559	-1.180047	0.208471
4	6	0	-0.832393	-0.002136	-0.012359
5	6	0	-1.476561	1.231992	-0.195461
6	6	0	-2.867034	1.261359	-0.176059
7	1	0	-4.683960	0.152000	0.070420
8	1	0	-3.527857	-1.996182	0.433285
9	1	0	-3.376242	2.204812	-0.334710
10	6	0	0.660407	-0.034545	-0.030237
11	8	0	1.340743	0.770892	0.582588
12	8	0	1.168255	-0.996973	-0.772151
13	1	0	2.167425	-1.007240	-0.744684
14	8	0	3.816042	-1.091702	-0.714449
15	1	0	4.136794	-0.342385	-0.163994
16	1	0	4.334446	-1.101575	-1.522481
17	8	0	4.044399	1.085682	0.801592
18	1	0	4.348478	1.203630	1.704210
19	1	0	3.065537	1.105340	0.819445
20	6	0	-0.912054	-2.521346	0.431335
21	1	0	-0.509944	-2.923478	-0.499160
22	1	0	-0.081343	-2.462128	1.136056
23	1	0	-1.641323	-3.228394	0.827483
24	6	0	-0.723139	2.516270	-0.427848
25	1	0	-0.222740	2.847318	0.482611
26	1	0	0.050628	2.405480	-1.189330
27	1	0	-1.409408	3.298698	-0.752673

Rotational constants (GHZ): 1.2645324 0.4352665 0.3465483

2,6-Dimethylbenzoate
Electronic Energy -651.806437
Free Energy -651.644211
Stoichiometry C9H13O4(1-)
Framework group C1[X(C9H13O4)]
Deg. of freedom 72
Full point group C1 NOp 1
Largest Abelian subgroup C1 NOp 1
Largest concise Abelian subgroup C1 NOp 1
Standard orientation:

Center Number	Atomic Number	Atomic Type	Coordinates (Angstroms) X	Y	Z
1	6	0	3.589988	-0.015340	0.027595
2	6	0	2.885682	-1.188575	-0.196226
3	6	0	1.492922	-1.195847	-0.209812
4	6	0	0.802254	0.006149	-0.013974
5	6	0	1.505403	1.197542	0.200838
6	6	0	2.897650	1.168935	0.229438
7	1	0	4.674714	-0.023741	0.043694
8	1	0	3.421265	-2.118775	-0.358835
9	1	0	3.442481	2.091134	0.406337
10	6	0	-0.722943	0.019924	-0.039801
11	8	0	-1.259157	0.720968	-0.917483
12	8	0	-1.287830	-0.683117	0.836528
13	8	0	-4.083411	0.777626	-1.243635
14	1	0	-3.107616	0.822589	-1.223633
15	1	0	-4.272274	0.195198	-0.492734
16	8	0	-3.921998	-0.821060	1.203958
17	1	0	-2.941330	-0.804685	1.024660
18	1	0	-4.032583	-0.213224	1.937879
19	6	0	0.775459	2.499936	0.401334

20	1	0	0.186432	2.747570	-0.482369
21	1	0	0.069487	2.431596	1.232851
22	1	0	1.478363	3.309322	0.611644
23	6	0	0.749309	-2.485618	-0.438899
24	1	0	0.132727	-2.729957	0.426776
25	1	0	0.067641	-2.400779	-1.288807
26	1	0	1.445514	-3.304213	-0.635363

Rotational constants (GHZ): 1.2552577 0.4228183 0.3564447

4-Bromobenzoic Acid
Electronic Energy -3147,306805
Free Energy -3147.195350
Stoichiometry C7H9BrO4
Framework group C1[X(C7H9BrO4)]
Deg. of freedom 57
Full point group C1 NOp 1
Largest Abelian subgroup C1 NOp 1
Largest concise Abelian subgroup C1 NOp 1
Standard orientation:

Center Number	Atomic Number	Atomic Type	Coordinates (Angstroms) X	Y	Z
1	6	0	-2.108117	-0.008899	-0.000098
2	6	0	-1.432943	1.205762	0.007909
3	6	0	-0.046379	1.230199	0.000865
4	6	0	0.669953	0.033974	-0.013119
5	6	0	-0.010619	-1.182588	-0.020667
6	6	0	-1.396284	-1.202976	-0.014588
7	1	0	-1.987812	2.135849	0.019364
8	1	0	0.479379	2.175358	0.006752
9	1	0	0.550846	-2.107745	-0.031563
10	1	0	-1.922382	-2.149661	-0.021143
11	6	0	2.154635	0.022988	-0.019835
12	8	0	2.803512	-1.015700	-0.021925
13	8	0	2.707914	1.219216	-0.023910
14	1	0	3.715285	1.182198	-0.034111
15	8	0	5.325432	1.288826	-0.097452
16	1	0	5.607182	0.352389	0.001032
17	1	0	5.690463	1.759442	0.661464
18	8	0	5.547608	-1.411308	0.145430
19	1	0	5.861179	-1.891458	-0.629683
20	1	0	4.574342	-1.372005	0.057932
21	35	0	-4.017877	-0.037811	0.008858

Rotational constants (GHZ): 2.5444086 0.1834738 0.1712898

4-Bromobenzoate
Electronic Energy -3146. 853887
Free Energy -3146.756469
Stoichiometry C7H8BrO4(1-)
Framework group C1[X(C7H8BrO4)]
Deg. of freedom 54
Full point group C1 NOp 1
Largest Abelian subgroup C1 NOp 1
Largest concise Abelian subgroup C1 NOp 1
Standard orientation:

Center Number	Atomic Number	Atomic Type	Coordinates (Angstroms) X	Y	Z
1	6	0	2.077163	-0.001164	0.000921
2	6	0	1.356323	-1.189392	0.000479
3	6	0	-0.031491	-1.160807	0.004843
4	6	0	-0.713936	0.054393	0.009644
5	6	0	0.016473	1.241430	0.010216
6	6	0	1.404267	1.214875	0.005811
7	1	0	1.876364	-2.140036	-0.003491
8	1	0	-0.592720	-2.085997	0.004105
9	1	0	-0.508404	2.187759	0.013780
10	1	0	1.961814	2.144021	0.006074

11	6	0	-2.225052	0.085791	0.012639
12	8	0	-2.787056	1.211907	0.014884
13	8	0	-2.830227	-1.020526	0.012206
14	8	0	-5.519977	1.392984	-0.010918
15	1	0	-4.536658	1.361324	0.001293
16	1	0	-5.759822	0.449925	-0.026449
17	8	0	-5.491610	-1.435844	-0.106484
18	1	0	-4.516831	-1.278906	-0.039067
19	1	0	-5.732236	-1.920172	0.690853
20	35	0	3.986771	-0.039339	-0.005484

Rotational constants (GHZ): 2.5233094 0.1841744 0.1717237

2-Bromobenzoic Acid
Electronic Energy -3147.296719
Free Energy -3147.186293
Stoichiometry C7H9BrO4
Framework group C1[X(C7H9BrO4)]
Deg. of freedom 57
Full point group C1 NOp 1
Largest Abelian subgroup C1 NOp 1
Largest concise Abelian subgroup C1 NOp 1
Standard orientation:

Center Number	Atomic Number	Atomic Type	Coordinates (Angstroms) X	Y	Z
1	6	0	3.031772	1.877169	0.145856
2	6	0	2.803397	0.514782	0.043710
3	6	0	1.505289	0.020593	0.012774
4	6	0	0.416984	0.894475	0.075760
5	6	0	0.672377	2.265406	0.148916
6	6	0	1.962411	2.759646	0.194753
7	1	0	4.049978	2.245667	0.176736
8	1	0	3.633366	-0.175770	-0.018697
9	1	0	-0.177993	2.934097	0.171107
10	1	0	2.132435	3.826565	0.264412
11	6	0	-1.029485	0.512067	0.073074
12	8	0	-1.868232	1.262135	-0.397668
13	8	0	-1.300905	-0.630556	0.648093
14	1	0	-2.279847	-0.845935	0.624114
15	8	0	-3.861131	-1.236501	0.594336
16	1	0	-4.350298	-0.493146	0.174939
17	1	0	-4.331392	-1.483176	1.394206
18	8	0	-4.591471	1.049677	-0.558531
19	1	0	-4.946343	1.224304	-1.433067
20	1	0	-3.640148	1.277776	-0.580390
21	35	0	1.334505	-1.860950	-0.206636

Rotational constants (GHZ): 0.8179317 0.4068218 0.2804956

2-Bromobenzoate
Electronic Energy -3146.846614
Free Energy -3146.749552
Stoichiometry C7H8BrO4(1-)
Framework group C1[X(C7H8BrO4)]
Deg. of freedom 54
Full point group C1 NOp 1
Largest Abelian subgroup C1 NOp 1
Largest concise Abelian subgroup C1 NOp 1
Standard orientation:

Center Number	Atomic Number	Atomic Type	Coordinates (Angstroms) X	Y	Z
1	6	0	3.083087	1.773724	0.229790
2	6	0	2.037877	2.687010	0.287107
3	6	0	0.731620	2.238651	0.193851
4	6	0	0.419242	0.882874	0.071729
5	6	0	1.484923	-0.009309	0.010363
6	6	0	2.804542	0.425183	0.078133
7	1	0	4.113200	2.106097	0.292796

8	1	0	2.242024	3.746388	0.395135
9	1	0	-0.100602	2.931089	0.201501
10	1	0	3.606641	-0.298311	0.011024
11	6	0	-1.068562	0.508453	0.045131
12	8	0	-1.401153	-0.516373	0.655920
13	8	0	-1.794696	1.328088	-0.570352
14	8	0	-4.160308	-1.084965	1.129059
15	1	0	-3.193147	-0.993191	1.035703
16	1	0	-4.486413	-0.372012	0.559875
17	8	0	-4.431189	1.043038	-0.867760
18	1	0	-3.456535	1.176124	-0.714638
19	1	0	-4.470368	0.564695	-1.698570
20	35	0	1.231074	-1.887391	-0.238697

Rotational constants (GHZ): 0.8009784 0.4026192 0.2849281

2-Chlorobenzoic Acid

Electronic Energy -1033.279442
Free Energy -1033.167636
Stoichiometry C7H9ClO4
Framework group C1[X(C7H9ClO4)]
Deg. of freedom 57
Full point group C1 NOp 1
Largest Abelian subgroup C1 NOp 1
Largest concise Abelian subgroup C1 NOp 1
Standard orientation:

Center Number	Atomic Number	Atomic Type	Coordinates (Angstroms) X	Y	Z
1	6	0	-3.505240	-0.910756	0.141919
2	6	0	-3.020906	0.371280	-0.069580
3	6	0	-1.652989	0.593258	-0.100741
4	6	0	-0.752171	-0.458175	0.065903
5	6	0	-1.260936	-1.743885	0.249549
6	6	0	-2.625242	-1.972354	0.299750
7	1	0	-4.575039	-1.076257	0.173057
8	1	0	-3.702141	1.198911	-0.217039
9	1	0	-0.564479	-2.564256	0.367760
10	1	0	-2.999344	-2.975770	0.458166
11	6	0	0.733839	-0.319815	0.059097
12	8	0	1.438634	-1.123261	-0.534299
13	8	0	1.194721	0.680882	0.767809
14	1	0	2.205070	0.753644	0.742222
15	8	0	3.775044	1.014321	0.760119
16	1	0	4.132704	0.245012	0.263041
17	1	0	4.142743	0.952420	1.649999
18	8	0	4.220231	-1.238361	-0.692042
19	1	0	4.520846	-1.123774	-1.600938
20	1	0	3.244784	-1.271717	-0.736220
21	17	0	-1.113666	2.225637	-0.434596

Rotational constants (GHZ): 1.2223449 0.4288751 0.3372817

2-Chlorobenzoate

Electronic Energy -1032.828919
Free Energy -1032.730415
Stoichiometry C7H8ClO4(1-)
Framework group C1[X(C7H8ClO4)]
Deg. of freedom 54
Full point group C1 NOp 1
Largest Abelian subgroup C1 NOp 1
Largest concise Abelian subgroup C1 NOp 1
Standard orientation:

Center Number	Atomic Number	Atomic Type	Coordinates (Angstroms) X	Y	Z
1	6	0	3.552376	-0.689565	0.104386
2	6	0	2.767658	-1.835607	0.127458
3	6	0	1.385667	-1.731511	0.072792
4	6	0	0.767135	-0.485737	-0.003550
5	6	0	1.572521	0.644335	-0.027138

6	6	0	2.954876	0.560020	0.025611
7	1	0	4.632035	-0.762946	0.146923
8	1	0	3.231957	-2.812207	0.188239
9	1	0	0.771228	-2.624155	0.090550
10	1	0	3.554689	1.460709	0.005661
11	6	0	-0.742377	-0.398769	-0.054085
12	8	0	-1.278005	-0.466358	-1.185598
13	8	0	-1.337483	-0.283709	1.046379
14	8	0	-4.033525	-0.419624	-1.386739
15	1	0	-3.052729	-0.443496	-1.359800
16	1	0	-4.265439	-0.264842	-0.453863
17	8	0	-4.017922	-0.040897	1.415964
18	1	0	-3.042020	-0.100493	1.281564
19	1	0	-4.180939	0.854345	1.732903
20	17	0	0.831739	2.235222	-0.130887

Rotational constants (GHZ): 1.2422535 0.4132900 0.3588427

3-Chlorobenzoic Acid
Electronic Energy -1033.287857
Free Energy -1033.174381
Stoichiometry C7H9ClO4
Framework group C1[X(C7H9ClO4)]
Deg. of freedom 57
Full point group C1 NOp 1
Largest Abelian subgroup C1 NOp 1
Largest concise Abelian subgroup C1 NOp 1
Standard orientation:

Center Number	Atomic Number	Atomic Type	Coordinates (Angstroms) X	Y	Z
1	6	0	2.924424	1.163945	0.011212
2	6	0	2.522901	-0.162384	0.001551
3	6	0	1.187636	-0.519560	-0.006937
4	6	0	0.225296	0.487844	-0.005389
5	6	0	0.610212	1.825463	0.004260
6	6	0	1.955336	2.158003	0.012485
7	1	0	3.976439	1.417957	0.017702
8	1	0	0.896484	-1.560277	-0.014676
9	1	0	-0.145286	2.599725	0.005323
10	1	0	2.257867	3.197408	0.020058
11	6	0	-1.224833	0.155328	-0.013479
12	8	0	-2.090053	1.020264	-0.012029
13	8	0	-1.483656	-1.134564	-0.022958
14	1	0	-2.473351	-1.341893	-0.028541
15	8	0	-3.998053	-1.825490	-0.079580
16	1	0	-4.495013	-0.980167	-0.009858
17	1	0	-4.246391	-2.345569	0.694353
18	8	0	-4.853942	0.749406	0.107000
19	1	0	-5.272794	1.133628	-0.671683
20	1	0	-3.901059	0.952592	0.025418
21	17	0	3.741347	-1.423657	0.000013

Rotational constants (GHZ): 1.5691885 0.3154463 0.2629838

3-Chlorobenzoate
Electronic Energy -1032.835369
Free Energy -1032.735699
Stoichiometry C7H8ClO4(1-)
Framework group C1[X(C7H8ClO4)]
Deg. of freedom 54
Full point group C1 NOp 1
Largest Abelian subgroup C1 NOp 1
Largest concise Abelian subgroup C1 NOp 1
Standard orientation:

Center Number	Atomic Number	Atomic Type	Coordinates (Angstroms) X	Y	Z
1	6	0	-2.892033	1.167049	-0.015413
2	6	0	-1.911120	2.148498	-0.059670
3	6	0	-0.567967	1.800882	-0.069480

4	6	0	-0.187570	0.462311	-0.036661
5	6	0	-1.165316	-0.528211	0.006634
6	6	0	-2.498320	-0.160822	0.017127
7	1	0	-3.941688	1.430616	-0.006676
8	1	0	-2.202693	3.191227	-0.086085
9	1	0	0.192148	2.569597	-0.103306
10	1	0	-0.879044	-1.570526	0.032101
11	6	0	1.275240	0.073216	-0.045713
12	8	0	1.550483	-1.152968	-0.029787
13	8	0	2.120530	1.006583	-0.068082
14	8	0	4.172904	-1.954675	-0.139709
15	1	0	3.222506	-1.704363	-0.109672
16	1	0	4.618833	-1.096190	-0.031055
17	8	0	4.804972	0.788159	0.133952
18	1	0	3.820204	0.861739	0.083116
19	1	0	5.040870	1.087136	1.019001
20	17	0	-3.728219	-1.414328	0.073568

Rotational constants (GHZ): 1.5568445 0.3161640 0.2633674

4-Chlorobenzoic Acid
Electronic Energy -1033.288630
Free Energy -1033.175525
Stoichiometry C7H9ClO4
Framework group C1[X(C7H9ClO4)]
Deg. of freedom 57
Full point group C1 NOp 1
Largest Abelian subgroup C1 NOp 1
Largest concise Abelian subgroup C1 NOp 1
Standard orientation:

Center Number	Atomic Number	Atomic Type	Coordinates (Angstroms) X	Y	Z
1	6	0	2.841072	0.026948	0.004223
2	6	0	2.190205	-1.196515	0.014387
3	6	0	0.805076	-1.217076	0.005009
4	6	0	0.084063	-0.025153	-0.012962
5	6	0	0.761203	1.191933	-0.022515
6	6	0	2.145111	1.225851	-0.014601
7	1	0	2.754071	-2.119631	0.029051
8	1	0	0.287524	-2.166396	0.012481
9	1	0	0.201268	2.117585	-0.036494
10	1	0	2.673671	2.169725	-0.022735
11	6	0	-1.401080	-0.018826	-0.021376
12	8	0	-2.049705	1.019218	-0.022807
13	8	0	-1.947534	-1.216532	-0.027688
14	1	0	-2.956532	-1.186832	-0.038097
15	8	0	-4.558878	-1.302047	-0.101331
16	1	0	-4.846649	-0.367491	-0.000717
17	1	0	-4.920584	-1.777055	0.656469
18	8	0	-4.799305	1.394374	0.149628
19	1	0	-5.119386	1.876074	-0.621864
20	1	0	-3.826062	1.365894	0.060301
21	17	0	4.590715	0.059120	0.015658

Rotational constants (GHZ): 2.5425254 0.2624028 0.2381627

4-Chlorobenzoate
Electronic Energy -1032.835597
Free Energy -1032.736691
Stoichiometry C7H8ClO4(1-)
Framework group C1[X(C7H8ClO4)]
Deg. of freedom 54
Full point group C1 NOp 1
Largest Abelian subgroup C1 NOp 1
Largest concise Abelian subgroup C1 NOp 1
Standard orientation:

Center Atomic Atomic Coordinates (Angstroms)
Number Number Type X Y Z

1	6	0	2.819057	-0.020043	0.006465
2	6	0	2.112812	-1.216300	-0.037533
3	6	0	0.723574	-1.172561	-0.050322
4	6	0	0.040964	0.045926	-0.020604
5	6	0	0.779947	1.230665	0.022573
6	6	0	2.169586	1.208397	0.036839
7	1	0	2.636330	-2.164954	-0.061290
8	1	0	0.163202	-2.099573	-0.083972
9	1	0	0.263115	2.182985	0.045703
10	1	0	2.737171	2.131018	0.070718
11	6	0	-1.470933	0.082939	-0.033810
12	8	0	-2.024536	1.215491	-0.013576
13	8	0	-2.075531	-1.025699	-0.064367
14	8	0	-4.773061	1.413938	-0.025136
15	1	0	-3.789816	1.376225	-0.023505
16	1	0	-5.010023	0.470040	-0.004397
17	8	0	-4.754501	-1.445282	0.005411
18	1	0	-3.779179	-1.278985	0.002194
19	1	0	-4.943138	-1.798031	0.881328
20	17	0	4.570196	-0.061203	0.024290

Rotational constants (GHZ): 2.4948660 0.2620053 0.2373215

2-Metoxybenzoic Acid

Electronic Energy -688.170734
Free Energy -688.017003
Stoichiometry C8H12O5
Framework group C1[X(C8H12O5)]
Deg. of freedom 69
Full point group C1 NOp 1
Largest Abelian subgroup C1 NOp 1
Largest concise Abelian subgroup C1 NOp 1
Standard orientation:

Center Number	Atomic Number	Atomic Type	Coordinates (Angstroms) X	Y	Z
1	6	0	3.298824	-1.276166	-0.135322
2	6	0	2.956147	0.060917	-0.004012
3	6	0	1.616210	0.444581	0.043790
4	6	0	0.613859	-0.541869	-0.037875
5	6	0	0.990364	-1.880467	-0.149719
6	6	0	2.319978	-2.257360	-0.208751
7	1	0	4.346977	-1.548001	-0.175820
8	1	0	3.737430	0.803531	0.065289
9	1	0	0.210419	-2.628881	-0.203913
10	1	0	2.588519	-3.300931	-0.310079
11	6	0	-0.845499	-0.267229	-0.002556
12	8	0	-1.640428	-1.103977	0.412086
13	8	0	-1.223877	0.900456	-0.475755
14	1	0	-2.221523	1.031913	-0.423380
15	8	0	-3.793570	1.429225	-0.381437
16	1	0	-4.218995	0.574247	-0.149635
17	1	0	-4.128731	1.666527	-1.254422
18	8	0	-4.411105	-1.121842	0.331869
19	1	0	-4.778835	-1.256111	1.212910
20	1	0	-3.440639	-1.193931	0.432418
21	8	0	1.235096	1.734189	0.200468
22	6	0	2.241185	2.738716	0.330157
23	1	0	1.702950	3.675581	0.452731
24	1	0	2.864479	2.556482	1.207824
25	1	0	2.862622	2.788429	-0.566031

Rotational constants (GHZ): 1.1793548 0.4201408 0.3171942

2-Metoxybenzoate

Electronic Energy -687.717205
Free Energy -687.577139
Stoichiometry C8H11O5(1-)
Framework group C1[X(C8H11O5)]
Deg. of freedom 66
Full point group C1 NOp 1
Largest Abelian subgroup C1 NOp 1

Largest concise Abelian subgroup C1 NOp 1

Standard orientation:

Center Number	Atomic Number	Atomic Type	Coordinates (Angstroms) X	Y	Z
1	6	0	3.322062	-1.166098	-0.136845
2	6	0	2.385440	-2.175424	-0.294385
3	6	0	1.034743	-1.854430	-0.258276
4	6	0	0.603682	-0.543193	-0.092748
5	6	0	1.562846	0.467969	0.073051
6	6	0	2.918406	0.149960	0.054482
7	1	0	4.381097	-1.395050	-0.154647
8	1	0	2.698726	-3.201789	-0.439303
9	1	0	0.290049	-2.633241	-0.370648
10	1	0	3.664539	0.919365	0.191537
11	6	0	-0.880177	-0.265146	-0.111587
12	8	0	-1.302792	0.666244	-0.841800
13	8	0	-1.605206	-1.023445	0.588038
14	8	0	-3.991186	1.224476	-0.919986
15	1	0	-3.023053	1.052245	-0.912227
16	1	0	-4.332348	0.536095	-0.322423
17	8	0	-4.293322	-0.981123	0.832006
18	1	0	-3.307360	-0.979853	0.754126
19	1	0	-4.479386	-0.770184	1.753556
20	8	0	1.103160	1.734124	0.282174
21	6	0	2.052407	2.776939	0.490021
22	1	0	2.699786	2.898739	-0.381162
23	1	0	1.467958	3.682458	0.636943
24	1	0	2.658314	2.585549	1.378520

Rotational constants (GHZ): 1.1675696 0.4147293 0.3280496

3-Metoxybenzoic Acid

Electronic Energy -688.176119

Free Energy -688.021973

Stoichiometry C8H12O5

Framework group C1[X(C8H12O5)]

Deg. of freedom 69

Full point group C1 NOp 1

Largest Abelian subgroup C1 NOp 1

Largest concise Abelian subgroup C1 NOp 1

Standard orientation:

Center Number	Atomic Number	Atomic Type	Coordinates (Angstroms) X	Y	Z
1	6	0	2.866096	1.041831	0.012478
2	6	0	2.452737	-0.285989	0.000460
3	6	0	1.095173	-0.594316	-0.010355
4	6	0	0.154104	0.425815	-0.009077
5	6	0	0.560668	1.760914	0.002843
6	6	0	1.911473	2.055007	0.013583
7	1	0	3.915829	1.299018	0.021118
8	1	0	0.792756	-1.632626	-0.019629
9	1	0	-0.178241	2.550579	0.003808
10	1	0	2.239578	3.087157	0.023156
11	6	0	-1.300643	0.119998	-0.019564
12	8	0	-2.152486	0.999534	-0.021852
13	8	0	-1.588182	-1.165219	-0.026965
14	1	0	-2.580684	-1.347771	-0.032691
15	8	0	-4.124803	-1.804859	-0.079357
16	1	0	-4.602747	-0.949403	-0.004263
17	1	0	-4.379113	-2.321232	0.695044
18	8	0	-4.913454	0.791679	0.118788
19	1	0	-5.327732	1.192456	-0.653922
20	1	0	-3.954961	0.965612	0.028964
21	8	0	3.304131	-1.352701	-0.001717
22	6	0	4.707043	-1.095743	0.008620
23	1	0	5.187282	-2.071493	0.004451
24	1	0	5.008633	-0.535938	-0.879552
25	1	0	4.997853	-0.548916	0.908410

Rotational constants (GHZ): 1.7374381 0.3044498 0.2598210

3-Metoxybenzoate
Electronic Energy -687.722538
Free Energy -687.581920
Stoichiometry C8H11O5(1-)
Framework group C1[X(C8H11O5)]
Deg. of freedom 66
Full point group C1 NOp 1
Largest Abelian subgroup C1 NOp 1
Largest concise Abelian subgroup C1 NOp 1
Standard orientation:

Center Number	Atomic Number	Atomic Type	Coordinates (Angstroms) X	Y	Z
1	6	0	2.867266	0.986220	0.017316
2	6	0	2.394641	-0.320822	-0.002963
3	6	0	1.024509	-0.569878	-0.011665
4	6	0	0.119235	0.481077	0.001605
5	6	0	0.589555	1.795498	0.022085
6	6	0	1.952513	2.035884	0.028847
7	1	0	3.927145	1.197902	0.024641
8	1	0	0.676574	-1.594301	-0.028431
9	1	0	-0.113903	2.616914	0.031767
10	6	0	-1.367926	0.201139	-0.004816
11	8	0	-2.144564	1.187137	0.066161
12	8	0	-1.735294	-1.002937	-0.080017
13	8	0	-4.860524	0.820875	-0.092755
14	1	0	-3.894526	0.999303	-0.047362
15	1	0	-4.895053	-0.150821	-0.041672
16	8	0	-4.261651	-1.937514	-0.000486
17	1	0	-3.335266	-1.589136	-0.001459
18	1	0	-4.371035	-2.382581	0.846910
19	1	0	2.321792	3.054527	0.043808
20	8	0	3.202340	-1.425619	-0.015306
21	6	0	4.613621	-1.225564	-0.016604
22	1	0	4.935561	-0.694946	0.882529
23	1	0	5.055216	-2.219548	-0.029485
24	1	0	4.930548	-0.674175	-0.904852

Rotational constants (GHZ): 1.6481476 0.3098852 0.2615602

4-Metoxybenzoic Acid
Electronic Energy -688.178959
Free Energy -688.025393
Stoichiometry C8H12O5
Framework group C1[X(C8H12O5)]
Deg. of freedom 69
Full point group C1 NOp 1
Largest Abelian subgroup C1 NOp 1
Largest concise Abelian subgroup C1 NOp 1
Standard orientation:

Center Number	Atomic Number	Atomic Type	Coordinates (Angstroms) X	Y	Z
1	6	0	-2.844676	0.234220	0.006450
2	6	0	-2.097906	1.415343	0.013691
3	6	0	-0.721183	1.360674	0.002968
4	6	0	-0.060793	0.127361	-0.014491
5	6	0	-0.815162	-1.042177	-0.021632
6	6	0	-2.200041	-1.001432	-0.011770
7	1	0	-2.618558	2.364721	0.027815
8	1	0	-0.151042	2.279805	0.008816
9	1	0	-0.312538	-2.000660	-0.035205
10	1	0	-2.761676	-1.924432	-0.018011
11	6	0	1.412320	0.035860	-0.023839
12	8	0	2.010830	-1.035062	-0.030481
13	8	0	2.031214	1.203008	-0.024914
14	1	0	3.033351	1.113010	-0.035161
15	8	0	4.656759	1.143694	-0.095385

16	1	0	4.887988	0.194376	0.009614
17	1	0	5.041434	1.599051	0.663007
18	8	0	4.721489	-1.564115	0.147815
19	1	0	5.015812	-2.061602	-0.623843
20	1	0	3.752139	-1.469822	0.050606
21	8	0	-4.192212	0.384373	0.018078
22	6	0	-5.008934	-0.787403	0.013937
23	1	0	-6.036111	-0.431107	0.025979
24	1	0	-4.836208	-1.378239	-0.887713
25	1	0	-4.820988	-1.394957	0.901310

Rotational constants (GHZ): 2.3158494 0.2713487 0.2435860

4-Metoxybenzoate
Electronic Energy -687.724018
Free Energy -687.584904
Stoichiometry C8H11O5(1-)
Framework group C1[X(C8H11O5)]
Deg. of freedom 66
Full point group C1 NOp 1
Largest Abelian subgroup C1 NOp 1
Largest concise Abelian subgroup C1 NOp 1
Standard orientation:

Center Number	Atomic Number	Atomic Type	Coordinates (Angstroms) X	Y	Z
1	6	0	-2.816106	0.278995	-0.001231
2	6	0	-2.018336	1.429146	-0.025918
3	6	0	-0.638983	1.314710	-0.031474
4	6	0	-0.016480	0.059203	-0.013083
5	6	0	-0.827401	-1.076326	0.010892
6	6	0	-2.215788	-0.981883	0.017124
7	1	0	-2.497751	2.402396	-0.040325
8	1	0	-0.030622	2.211888	-0.050453
9	1	0	-0.366465	-2.057670	0.025104
10	1	0	-2.812838	-1.885105	0.035976
11	6	0	1.483973	-0.062851	-0.018607
12	8	0	1.979748	-1.225031	-0.001978
13	8	0	2.157555	1.008658	-0.039964
14	8	0	4.699552	-1.584831	-0.023789
15	1	0	3.719690	-1.482122	-0.017000
16	1	0	4.998152	-0.658703	-0.013997
17	8	0	4.851283	1.271601	-0.023611
18	1	0	3.867274	1.159666	-0.008085
19	1	0	5.073434	1.632343	0.841091
20	8	0	-4.162197	0.485342	0.003095
21	6	0	-5.017813	-0.656673	0.030139
22	1	0	-6.034145	-0.264728	0.030454
23	1	0	-4.867950	-1.281241	-0.855354
24	1	0	-4.854684	-1.248575	0.935512

Rotational constants (GHZ): 2.3203832 0.2702577 0.2426244

- **B3PW91 6-311G+(d,p) SMD**

H_2O
Electronic Energy -229.332309
Free Energy -229.293786
Stoichiometry H6O3
Framework group C1[X(H6O3)]
Deg. of freedom 21
Full point group C1 NOp 1
Largest Abelian subgroup C1 NOp 1
Largest concise Abelian subgroup C1 NOp 1
Standard orientation:

Center Number	Atomic Number	Atomic Type	Coordinates (Angstroms) X	Y	Z
1	8	0	0.000010	0.892376	0.000148
2	1	0	0.000196	1.480963	0.764933

3	1	0	-0.000277	1.483600	-0.762617
4	8	0	-2.496266	-0.455010	-0.000467
5	1	0	-2.292654	-1.395867	0.002233
6	1	0	-1.624795	-0.015841	-0.000130
7	8	0	2.496269	-0.455014	-0.000432
8	1	0	2.292610	-1.395862	0.001891
9	1	0	1.624819	-0.015802	-0.000302

Rotational constants (GHZ): 17.4917501 2.3350517 2.0799858

OH^-

Electronic Energy -228.849931
Free Energy -228.824687
Stoichiometry H5O3(1-)
Framework group C1[X(H5O3)]
Deg. of freedom 18
Full point group C1 NOp 1
Largest Abelian subgroup C1 NOp 1
Largest concise Abelian subgroup C1 NOp 1
Standard orientation:

Center Number	Atomic Number	Atomic Type	Coordinates (Angstroms) X	Y	Z
1	8	0	-0.000225	0.657416	0.000044
2	1	0	-0.000769	1.616660	-0.000144
3	8	0	-2.351148	-0.340430	0.086619
4	1	0	-2.355030	-0.801681	-0.754038
5	1	0	-1.410034	0.086466	0.103715
6	8	0	2.351414	-0.340291	-0.086624
7	1	0	1.410559	0.086532	-0.103892
8	1	0	2.354945	-0.801536	0.754044

Rotational constants (GHZ): 31.7538037 2.6124257 2.4454439

Benzoic Acid

Electronic Energy -573.684789
Free Energy -573.561104
Stoichiometry C7H10O4
Framework group C1[X(C7H10O4)]
Deg. of freedom 57
Full point group C1 NOp 1
Largest Abelian subgroup C1 NOp 1
Largest concise Abelian subgroup C1 NOp 1
Standard orientation:

Center Number	Atomic Number	Atomic Type	Coordinates (Angstroms) X	Y	Z
1	6	0	3.735768	0.106891	0.002680
2	6	0	3.097058	-1.126215	-0.025610
3	6	0	1.712108	-1.190953	-0.027032
4	6	0	0.963430	-0.016885	-0.000726
5	6	0	1.605855	1.217996	0.027132
6	6	0	2.989871	1.279025	0.029233
7	1	0	4.818361	0.154828	0.004047
8	1	0	3.679744	-2.039066	-0.046699
9	1	0	1.202615	-2.144733	-0.049253
10	1	0	1.004391	2.117522	0.047711
11	1	0	3.488943	2.240104	0.051620
12	6	0	-0.523535	-0.045995	-0.002089
13	8	0	-1.192883	0.974044	0.004883
14	8	0	-1.033631	-1.259598	-0.012255
15	1	0	-2.033438	-1.251274	-0.015869
16	8	0	-3.675401	-1.288071	-0.039580
17	1	0	-3.988039	-0.355181	-0.031632
18	1	0	-4.174075	-1.766065	0.627238
19	8	0	-3.893652	1.363338	0.043162
20	1	0	-4.202595	1.992395	-0.612469
21	1	0	-2.914707	1.390592	0.034105

Rotational constants (GHZ): 2.5684597 0.4655694 0.3946595

Benzoate
Electronic Energy -573.226511
Free Energy -573.117861
Stoichiometry C7H9O4(1-)
Framework group C1[X(C7H9O4)]
Deg. of freedom 54
Full point group C1 NOp 1
Largest Abelian subgroup C1 NOp 1
Largest concise Abelian subgroup C1 NOp 1
Standard orientation:

Center Number	Atomic Number	Atomic Type	Coordinates (Angstroms) X	Y	Z
1	6	0	-3.728847	0.075164	0.016945
2	6	0	-2.987947	1.254897	-0.015117
3	6	0	-1.597447	1.205018	-0.027238
4	6	0	-0.929667	-0.023231	-0.007944
5	6	0	-1.681758	-1.201525	0.023930
6	6	0	-3.072246	-1.153779	0.036603
7	1	0	-4.813562	0.113388	0.026505
8	1	0	-3.494486	2.214685	-0.030996
9	1	0	-1.021665	2.123231	-0.052120
10	1	0	-1.170525	-2.157442	0.038976
11	1	0	-3.644617	-2.075611	0.061674
12	6	0	0.582048	-0.077788	-0.021074
13	8	0	1.125276	-1.216414	-0.011139
14	8	0	1.202610	1.023136	-0.041479
15	8	0	3.867084	-1.447655	-0.020541
16	1	0	2.884000	-1.395929	-0.017729
17	1	0	4.117459	-0.507113	-0.016331
18	8	0	3.884618	1.413169	-0.034678
19	1	0	2.907430	1.256800	-0.015491
20	1	0	4.094438	1.777563	0.831580

Rotational constants (GHZ): 2.5026876 0.4622187 0.3906360

4-Cyanobenzoic Acid
Electronic Energy -665.910056
Free Energy -665.790683
Stoichiometry C8H9NO4
Framework group C1[X(C8H9NO4)]
Deg. of freedom 60
Full point group C1 NOp 1
Largest Abelian subgroup C1 NOp 1
Largest concise Abelian subgroup C1 NOp 1
Standard orientation:

Center Number	Atomic Number	Atomic Type	Coordinates (Angstroms) X	Y	Z
1	6	0	3.000493	0.037899	0.000660
2	6	0	2.342699	-1.191710	-0.025493
3	6	0	0.959623	-1.229468	-0.025870
4	6	0	0.230936	-0.043774	-0.001014
5	6	0	0.889881	1.181565	0.024506
6	6	0	2.271556	1.227502	0.025871
7	1	0	2.918243	-2.107919	-0.045351
8	1	0	0.436518	-2.175488	-0.046170
9	1	0	0.302906	2.090231	0.043876
10	1	0	2.792226	2.175946	0.046338
11	6	0	-1.261291	-0.049941	-0.001330
12	8	0	-1.906496	0.983294	0.002598
13	8	0	-1.783347	-1.254597	-0.007073
14	1	0	-2.787218	-1.239316	-0.011243
15	8	0	-4.403093	-1.251510	-0.043120
16	1	0	-4.718237	-0.319178	-0.031168
17	1	0	-4.920201	-1.744273	0.598546
18	8	0	-4.620755	1.395754	0.046924
19	1	0	-4.945074	2.035091	-0.591284
20	1	0	-3.644247	1.436877	0.032740
21	6	0	4.433440	0.079717	0.001548
22	7	0	5.581514	0.113394	0.002261

Rotational constants (GHZ): 2.5600692 0.2780027 0.2509715

4-Cyanobenzoate

Electronic Energy -665.4540270
Free Energy -665.349332
Stoichiometry C8H8NO4(1-)
Framework group C1[X(C8H8NO4)]
Deg. of freedom 57
Full point group C1 NOp 1
Largest Abelian subgroup C1 NOp 1
Largest concise Abelian subgroup C1 NOp 1
Standard orientation:

Center Number	Atomic Number	Atomic Type	Coordinates (Angstroms) X	Y	Z
1	6	0	3.000511	-0.003611	-0.000659
2	6	0	2.283426	-1.209869	-0.066660
3	6	0	0.894422	-1.187699	-0.066143
4	6	0	0.196052	0.022380	-0.001106
5	6	0	0.916764	1.219330	0.062927
6	6	0	2.305726	1.215868	0.064207
7	1	0	2.822907	-2.148863	-0.117649
8	1	0	0.315836	-2.101994	-0.116518
9	1	0	0.354204	2.143883	0.110850
10	1	0	2.862337	2.144821	0.114345
11	6	0	-1.342486	0.042071	-0.000024
12	8	0	-1.875392	1.170279	0.050653
13	8	0	-1.893079	-1.090661	-0.049038
14	8	0	-4.739548	1.481420	-0.127760
15	1	0	-3.764675	1.490368	-0.064241
16	1	0	-4.926961	0.530093	-0.159539
17	8	0	-4.568925	-1.478385	0.042441
18	1	0	-3.590819	-1.325879	-0.052199
19	1	0	-4.677427	-1.678920	0.976111
20	6	0	4.429985	-0.017268	0.000842
21	7	0	5.587680	-0.028850	0.002596

Rotational constants (GHZ): 2.4272317 0.2736266 0.2462903

2,6-Dimethylbenzoic Acid

Electronic Energy -652.302480
Free Energy -652.128035
Stoichiometry C9H14O4
Framework group C1[X(C9H14O4)]
Deg. of freedom 75
Full point group C1 NOp 1
Largest Abelian subgroup C1 NOp 1
Largest concise Abelian subgroup C1 NOp 1
Standard orientation:

Center Number	Atomic Number	Atomic Type	Coordinates (Angstroms) X	Y	Z
1	6	0	-3.601314	0.109154	0.047875
2	6	0	-2.952020	-1.097216	0.246430
3	6	0	-1.563559	-1.180047	0.208471
4	6	0	-0.832393	-0.002136	-0.012359
5	6	0	-1.476561	1.231992	-0.195461
6	6	0	-2.867034	1.261359	-0.176059
7	1	0	-4.683960	0.152000	0.070420
8	1	0	-3.527857	-1.996182	0.433285
9	1	0	-3.376242	2.204812	-0.334710
10	6	0	0.660407	-0.034545	-0.030237
11	8	0	1.340743	0.770892	0.582588
12	8	0	1.168255	-0.996973	-0.772151
13	1	0	2.167425	-1.007240	-0.744684
14	8	0	3.816042	-1.091702	-0.714449
15	1	0	4.136794	-0.342385	-0.163994
16	1	0	4.334446	-1.101575	-1.522481
17	8	0	4.044399	1.085682	0.801592

18	1	0	4.348478	1.203630	1.704210
19	1	0	3.065537	1.105340	0.819445
20	6	0	-0.912054	-2.521346	0.431335
21	1	0	-0.509944	-2.923478	-0.499160
22	1	0	-0.081343	-2.462128	1.136056
23	1	0	-1.641323	-3.228394	0.827483
24	6	0	-0.723139	2.516270	-0.427848
25	1	0	-0.222740	2.847318	0.482611
26	1	0	0.050628	2.405480	-1.189330
27	1	0	-1.409408	3.298698	-0.752673

Rotational constants (GHZ): 1.2645324 0.4352665 0.3465483

2,6-Dimethylbenzoate
Electronic Energy -651.845845
Free Energy -651.685748
Stoichiometry C9H13O4(1-)
Framework group C1[X(C9H13O4)]
Deg. of freedom 72
Full point group C1 NOp 1
Largest Abelian subgroup C1 NOp 1
Largest concise Abelian subgroup C1 NOp 1
Standard orientation:

Center Number	Atomic Number	Atomic Type	Coordinates (Angstroms) X	Y	Z
1	6	0	3.589988	-0.015340	0.027595
2	6	0	2.885682	-1.188575	-0.196226
3	6	0	1.492922	-1.195847	-0.209812
4	6	0	0.802254	0.006149	-0.013974
5	6	0	1.505403	1.197542	0.200838
6	6	0	2.897650	1.168935	0.229438
7	1	0	4.674714	-0.023741	0.043694
8	1	0	3.421265	-2.118775	-0.358835
9	1	0	3.442481	2.091134	0.406337
10	6	0	-0.722943	0.019924	-0.039801
11	8	0	-1.259157	0.720968	-0.917483
12	8	0	-1.287830	-0.683117	0.836528
13	8	0	-4.083411	0.777626	-1.243635
14	1	0	-3.107616	0.822589	-1.223633
15	1	0	-4.272274	0.195198	-0.492734
16	8	0	-3.921998	-0.821060	1.203958
17	1	0	-2.941330	-0.804685	1.024660
18	1	0	-4.032583	-0.213224	1.937879
19	6	0	0.775459	2.499936	0.401334
20	1	0	0.186432	2.747570	-0.482369
21	1	0	0.069487	2.431596	1.232851
22	1	0	1.478363	3.309322	0.611644
23	6	0	0.749309	-2.485618	-0.438899
24	1	0	0.132727	-2.729957	0.426776
25	1	0	0.067641	-2.400779	-1.288807
26	1	0	1.445514	-3.304213	-0.635363

Rotational constants (GHZ): 1.2552577 0.4228183 0.3564447

4-Bromobenzoic Acid
Electronic Energy -3147.212556
Free Energy -3147.102261
Stoichiometry C7H9BrO4
Framework group C1[X(C7H9BrO4)]
Deg. of freedom 57
Full point group C1 NOp 1
Largest Abelian subgroup C1 NOp 1
Largest concise Abelian subgroup C1 NOp 1
Standard orientation:

Center Number	Atomic Number	Atomic Type	Coordinates (Angstroms) X	Y	Z
1	6	0	-2.114012	-0.009203	-0.000135
2	6	0	-1.436369	1.208247	0.007660

3	6	0	-0.046613	1.232707	0.000310
4	6	0	0.673332	0.033261	-0.013550
5	6	0	-0.011270	-1.186274	-0.020785
6	6	0	-1.400065	-1.206270	-0.014616
7	1	0	-1.991744	2.140022	0.019169
8	1	0	0.479082	2.179877	0.006063
9	1	0	0.550579	-2.113264	-0.031624
10	1	0	-1.926923	-2.154485	-0.021076
11	6	0	2.158226	0.019624	-0.020228
12	8	0	2.805407	-1.024475	-0.020921
13	8	0	2.711074	1.218910	-0.025982
14	1	0	3.721865	1.178924	-0.036727
15	8	0	5.325194	1.297862	-0.097116
16	1	0	5.603903	0.359675	0.003040
17	1	0	5.660100	1.750468	0.685552
18	8	0	5.572935	-1.411442	0.146778
19	1	0	5.879465	-1.844245	-0.657810
20	1	0	4.599625	-1.374902	0.061347
21	35	0	-4.023777	-0.037756	0.009087

Rotational constants (GHZ): 2.5322608 0.1828346 0.1706863

4-Bromobenzoate

Electronic Energy -3146.755194
Free Energy -3146.660243
Stoichiometry C7H8BrO4(1-)
Framework group C1[X(C7H8BrO4)]
Deg. of freedom 54
Full point group C1 NOp 1
Largest Abelian subgroup C1 NOp 1
Largest concise Abelian subgroup C1 NOp 1
Standard orientation:

Center Number	Atomic Number	Atomic Type	Coordinates (Angstroms) X	Y	Z
1	6	0	2.070445	-0.001051	0.000332
2	6	0	1.367896	-1.200233	-0.029558
3	6	0	-0.022062	-1.161774	-0.035773
4	6	0	-0.708990	0.054192	-0.012956
5	6	0	0.025448	1.241916	0.016894
6	6	0	1.415790	1.225115	0.023986
7	1	0	1.889319	-2.149869	-0.047506
8	1	0	-0.578787	-2.091369	-0.058932
9	1	0	-0.494627	2.192662	0.034808
10	1	0	1.974433	2.153190	0.047223
11	6	0	-2.221493	0.085174	-0.020444
12	8	0	-2.779076	1.215702	-0.005050
13	8	0	-2.821222	-1.026128	-0.041701
14	8	0	-5.529129	1.402328	-0.018544
15	1	0	-4.545794	1.368256	-0.014111
16	1	0	-5.762700	0.457281	-0.012126
17	8	0	-5.500659	-1.456692	-0.027212
18	1	0	-4.526276	-1.286632	-0.009664
19	1	0	-5.703852	-1.824197	0.839331
20	35	0	3.977907	-0.038887	0.008747

Rotational constants (GHZ): 2.4945812 0.1844157 0.1718140

2-Bromobenzoic Acid

Electronic Energy -3147.202752
Free Energy -3147.093360
Stoichiometry C7H9BrO4
Framework group C1[X(C7H9BrO4)]
Deg. of freedom 57
Full point group C1 NOp 1
Largest Abelian subgroup C1 NOp 1
Largest concise Abelian subgroup C1 NOp 1
Standard orientation:

Center Atomic Atomic Coordinates (Angstroms)

Number	Number	Type	X	Y	Z
1	6	0	-3.443176	1.224295	0.019838
2	6	0	-2.623090	2.350745	0.015895
3	6	0	-1.240640	2.206668	0.003694
4	6	0	-0.671278	0.928795	-0.003258
5	6	0	-1.498521	-0.198878	0.001285
6	6	0	-2.879481	-0.050495	0.012268
7	1	0	-3.061513	3.342978	0.022025
8	1	0	-0.604116	3.083222	0.000319
9	1	0	-3.517289	-0.927950	0.014946
10	6	0	0.801000	0.735387	-0.014811
11	8	0	1.316999	-0.379379	-0.009560
12	8	0	1.494952	1.858821	-0.031742
13	1	0	2.493410	1.696643	-0.045567
14	8	0	4.099072	1.620096	-0.112258
15	1	0	4.262568	0.655819	-0.005747
16	1	0	4.489653	2.034522	0.665740
17	8	0	4.018007	-1.097414	0.151857
18	1	0	4.266442	-1.570023	-0.650366
19	1	0	3.055925	-0.943900	0.069410
20	1	0	-4.507078	1.338018	0.028718
21	35	0	-0.713689	-1.940155	-0.008438

Rotational constants (GHZ): 0.8308145 0.4470956 0.2911682

2-Bromobenzoate
Electronic Energy -3146.748254
Free Energy -3146.650051
Stoichiometry C7H8BrO4(1-)
Framework group C1[X(C7H8BrO4)]
Deg. of freedom 54
Full point group C1 NOp 1
Largest Abelian subgroup C1 NOp 1
Largest concise Abelian subgroup C1 NOp 1
Standard orientation:

Center Number	Atomic Number	Atomic Type	Coordinates (Angstroms) X	Y	Z
1	6	0	3.326054	-1.400706	0.068535
2	6	0	2.428090	-2.465342	0.058099
3	6	0	1.060365	-2.219130	0.019241
4	6	0	0.555328	-0.913797	-0.008564
5	6	0	1.477071	0.130882	0.002902
6	6	0	2.850282	-0.093606	0.040323
7	1	0	2.792598	-3.487485	0.079893
8	1	0	0.361887	-3.051013	0.010444
9	1	0	3.542043	0.741244	0.047670
10	6	0	-0.951611	-0.706598	-0.051550
11	8	0	-1.474464	-0.692520	-1.186407
12	8	0	-1.518688	-0.609816	1.065116
13	8	0	-4.236463	-0.315096	-1.435730
14	1	0	-3.265790	-0.451242	-1.411268
15	1	0	-4.442811	-0.220116	-0.491480
16	8	0	-4.141787	-0.186287	1.431102
17	1	0	-3.168483	-0.339070	1.297243
18	1	0	-4.203087	0.650690	1.901067
19	1	0	4.395927	-1.580181	0.098020
20	35	0	0.871013	1.947906	-0.037090

Rotational constants (GHZ): 0.8113043 0.4005286 0.3051321

2-Chlorobenzoic Acid
Electronic Energy -1033.245591
Free Energy -1033.133888
Stoichiometry C7H9ClO4
Framework group C1[X(C7H9ClO4)]
Deg. of freedom 57
Full point group C1 NOp 1
Largest Abelian subgroup C1 NOp 1
Largest concise Abelian subgroup C1 NOp 1

Standard orientation:

Center Number	Atomic Number	Atomic Type	Coordinates (Angstroms) X	Y	Z
1	6	0	-3.505240	-0.910756	0.141919
2	6	0	-3.020906	0.371280	-0.069580
3	6	0	-1.652989	0.593258	-0.100741
4	6	0	-0.752171	-0.458175	0.065903
5	6	0	-1.260936	-1.743885	0.249549
6	6	0	-2.625242	-1.972354	0.299750
7	1	0	-4.575039	-1.076257	0.173057
8	1	0	-3.702141	1.198911	-0.217039
9	1	0	-0.564479	-2.564256	0.367760
10	1	0	-2.999344	-2.975770	0.458166
11	6	0	0.733839	-0.319815	0.059097
12	8	0	1.438634	-1.123261	-0.534299
13	8	0	1.194721	0.680882	0.767809
14	1	0	2.205070	0.753644	0.742222
15	8	0	3.775044	1.014321	0.760119
16	1	0	4.132704	0.245012	0.263041
17	1	0	4.142743	0.952420	1.649999
18	8	0	4.220231	-1.238361	-0.692042
19	1	0	4.520846	-1.123774	-1.600938
20	1	0	3.244784	-1.271717	-0.736220
21	17	0	-1.113666	2.225637	-0.434596

Rotational constants (GHZ): 1.2223449 0.4288751 0.3372817

2-Chlorobenzoate
Electronic Energy -1032.791059
Free Energy -1032.693850
Stoichiometry C7H8ClO4(1-)
Framework group C1[X(C7H8ClO4)]
Deg. of freedom 54
Full point group C1 NOp 1
Largest Abelian subgroup C1 NOp 1
Largest concise Abelian subgroup C1 NOp 1

Standard orientation:

Center Number	Atomic Number	Atomic Type	Coordinates (Angstroms) X	Y	Z
1	6	0	3.552376	-0.689565	0.104386
2	6	0	2.767658	-1.835607	0.127458
3	6	0	1.385667	-1.731511	0.072792
4	6	0	0.767135	-0.485737	-0.003550
5	6	0	1.572521	0.644335	-0.027138
6	6	0	2.954876	0.560020	0.025611
7	1	0	4.632035	-0.762946	0.146923
8	1	0	3.231957	-2.812207	0.188239
9	1	0	0.771228	-2.624155	0.090550
10	1	0	3.554689	1.460709	0.005661
11	6	0	-0.742377	-0.398769	-0.054085
12	8	0	-1.278005	-0.466358	-1.185598
13	8	0	-1.337483	-0.283709	1.046379
14	8	0	-4.033525	-0.419624	-1.386739
15	1	0	-3.052729	-0.443496	-1.359800
16	1	0	-4.265439	-0.264842	-0.453863
17	8	0	-4.017922	-0.040897	1.415964
18	1	0	-3.042020	-0.100493	1.281564
19	1	0	-4.180939	0.854345	1.732903
20	17	0	0.831739	2.235222	-0.130887

Rotational constants (GHZ): 1.2422535 0.4132900 0.3588427

3-Chlorobenzoic Acid
Electronic Energy -1033.253900
Free Energy -1033.141967
Stoichiometry C7H9ClO4
Framework group C1[X(C7H9ClO4)]
Deg. of freedom 57
Full point group C1 NOp 1

Largest Abelian subgroup C1 NOp 1
Largest concise Abelian subgroup C1 NOp 1
Standard orientation:

Center Number	Atomic Number	Atomic Type	Coordinates (Angstroms) X	Y	Z
1	6	0	2.924424	1.163945	0.011212
2	6	0	2.522901	-0.162384	0.001551
3	6	0	1.187636	-0.519560	-0.006937
4	6	0	0.225296	0.487844	-0.005389
5	6	0	0.610212	1.825463	0.004260
6	6	0	1.955336	2.158003	0.012485
7	1	0	3.976439	1.417957	0.017702
8	1	0	0.896484	-1.560277	-0.014676
9	1	0	-0.145286	2.599725	0.005323
10	1	0	2.257867	3.197408	0.020058
11	6	0	-1.224833	0.155328	-0.013479
12	8	0	-2.090053	1.020264	-0.012029
13	8	0	-1.483656	-1.134564	-0.022958
14	1	0	-2.473351	-1.341893	-0.028541
15	8	0	-3.998053	-1.825490	-0.079580
16	1	0	-4.495013	-0.980167	-0.009858
17	1	0	-4.246391	-2.345569	0.694353
18	8	0	-4.853942	0.749406	0.107000
19	1	0	-5.272794	1.133628	-0.671683
20	1	0	-3.901059	0.952592	0.025418
21	17	0	3.741347	-1.423657	0.000013

Rotational constants (GHZ): 1.5691885 0.3154463 0. 2629838

3-Chlorobenzoate
Electronic Energy -1032.797043
Free Energy -1032.699179
Stoichiometry C7H8ClO4(1-)
Framework group C1[X(C7H8ClO4)]
Deg. of freedom 54
Full point group C1 NOp 1
Largest Abelian subgroup C1 NOp 1
Largest concise Abelian subgroup C1 NOp 1
Standard orientation:

Center Number	Atomic Number	Atomic Type	Coordinates (Angstroms) X	Y	Z
1	6	0	-2.892145	1.167033	-0.016137
2	6	0	-1.911225	2.148487	-0.059948
3	6	0	-0.568057	1.800877	-0.068936
4	6	0	-0.187672	0.462323	-0.035767
5	6	0	-1.165424	-0.528227	0.007065
6	6	0	-2.498421	-0.160829	0.016779
7	1	0	-3.941809	1.430578	-0.008025
8	1	0	-2.202799	3.191207	-0.086661
9	1	0	0.192067	2.569597	-0.102384
10	1	0	-0.879161	-1.570535	0.032786
11	6	0	1.275164	0.073232	-0.043932
12	8	0	1.550410	-1.152931	-0.028125
13	8	0	2.120446	1.006643	-0.065399
14	8	0	4.173292	-1.954565	-0.139797
15	1	0	3.222843	-1.704747	-0.109050
16	1	0	4.618763	-1.095675	-0.032098
17	8	0	4.805293	0.787870	0.130645
18	1	0	3.820429	0.861656	0.081797
19	1	0	5.043160	1.088165	1.014728
20	17	0	-3.728373	-1.414339	0.072681

Rotational constants (GHZ): 1.5570764 0.3161357 0.2633442

4-Chlorobenzoic Acid
Electronic Energy -1033.254806
Free Energy -1033.142797
Stoichiometry C7H9ClO4
Framework group C1[X(C7H9ClO4)]

Deg. of freedom 57
Full point group C1 NOp 1
Largest Abelian subgroup C1 NOp 1
Largest concise Abelian subgroup C1 NOp 1
Standard orientation:

Center Number	Atomic Number	Atomic Type	Coordinates (Angstroms) X	Y	Z
1	6	0	2.841072	0.026948	0.004223
2	6	0	2.190205	-1.196515	0.014387
3	6	0	0.805076	-1.217076	0.005009
4	6	0	0.084063	-0.025153	-0.012962
5	6	0	0.761203	1.191933	-0.022515
6	6	0	2.145111	1.225851	-0.014601
7	1	0	2.754071	-2.119631	0.029051
8	1	0	0.287524	-2.166396	0.012481
9	1	0	0.201268	2.117585	-0.036494
10	1	0	2.673671	2.169725	-0.022735
11	6	0	-1.401080	-0.018826	-0.021376
12	8	0	-2.049705	1.019218	-0.022807
13	8	0	-1.947534	-1.216532	-0.027688
14	1	0	-2.956532	-1.186832	-0.038097
15	8	0	-4.558878	-1.302047	-0.101331
16	1	0	-4.846649	-0.367491	-0.000717
17	1	0	-4.920584	-1.777055	0.656469
18	8	0	-4.799305	1.394374	0.149628
19	1	0	-5.119386	1.876074	-0.621864
20	1	0	-3.826062	1.365894	0.060301
21	17	0	4.590715	0.059120	0.015658

Rotational constants (GHZ): 2.5425254 0.2624028 0.2381627

4-Chlorobenzoate
Electronic Energy -1032.797319
Free Energy -1032.699682
Stoichiometry C7H8ClO4(1-)
Framework group C1[X(C7H8ClO4)]
Deg. of freedom 54
Full point group C1 NOp 1
Largest Abelian subgroup C1 NOp 1
Largest concise Abelian subgroup C1 NOp 1
Standard orientation:

Center Number	Atomic Number	Atomic Type	Coordinates (Angstroms) X	Y	Z
1	6	0	2.810550	-0.020753	-0.001734
2	6	0	2.106204	-1.213631	-0.001527
3	6	0	0.720092	-1.170186	0.003746
4	6	0	0.042751	0.046488	0.008707
5	6	0	0.778393	1.228832	0.008541
6	6	0	2.164897	1.204884	0.003322
7	1	0	2.628731	-2.161286	-0.005603
8	1	0	0.158968	-2.095005	0.003580
9	1	0	0.262185	2.179492	0.012176
10	1	0	2.733140	2.125827	0.002986
11	6	0	-1.468317	0.084420	0.012463
12	8	0	-2.022280	1.213448	0.013300
13	8	0	-2.075010	-1.020038	0.013964
14	8	0	-4.758419	1.405075	-0.007489
15	1	0	-3.775605	1.371047	0.003192
16	1	0	-5.000805	0.462617	-0.024689
17	8	0	-4.740769	-1.423288	-0.106855
18	1	0	-3.765653	-1.271772	-0.038340
19	1	0	-4.985175	-1.905189	0.690827
20	17	0	4.564036	-0.062802	-0.008741

Rotational constants (GHZ): 2.5195961 0.2631042 0.2383770

2-Metoxybenzoic Acid
Electronic Energy -688.192447
Free Energy -688.040140
Stoichiometry C8H12O5
Framework group C1[X(C8H12O5)]
Deg. of freedom 69
Full point group C1 NOp 1
Largest Abelian subgroup C1 NOp 1
Largest concise Abelian subgroup C1 NOp 1
Standard orientation:

Center Number	Atomic Number	Atomic Type	Coordinates (Angstroms) X	Y	Z
1	6	0	3.298824	-1.276166	-0.135322
2	6	0	2.956147	0.060917	-0.004012
3	6	0	1.616210	0.444581	0.043790
4	6	0	0.613859	-0.541869	-0.037875
5	6	0	0.990364	-1.880467	-0.149719
6	6	0	2.319978	-2.257360	-0.208751
7	1	0	4.346977	-1.548001	-0.175820
8	1	0	3.737430	0.803531	0.065289
9	1	0	0.210419	-2.628881	-0.203913
10	1	0	2.588519	-3.300931	-0.310079
11	6	0	-0.845499	-0.267229	-0.002556
12	8	0	-1.640428	-1.103977	0.412086
13	8	0	-1.223877	0.900456	-0.475755
14	1	0	-2.221523	1.031913	-0.423380
15	8	0	-3.793570	1.429225	-0.381437
16	1	0	-4.218995	0.574247	-0.149635
17	1	0	-4.128731	1.666527	-1.254422
18	8	0	-4.411105	-1.121842	0.331869
19	1	0	-4.778835	-1.256111	1.212910
20	1	0	-3.440639	-1.193931	0.432418
21	8	0	1.235096	1.734189	0.200468
22	6	0	2.241185	2.738716	0.330157
23	1	0	1.702950	3.675581	0.452731
24	1	0	2.864479	2.556482	1.207824
25	1	0	2.862622	2.788429	-0.566031

Rotational constants (GHZ): 1.1793548 0.4201408 0.3171942

2-Metoxybenzoate
Electronic Energy -687.734604
Free Energy
Stoichiometry C8H11O5(1-)
Framework group C1[X(C8H11O5)]
Deg. of freedom 66
Full point group C1 NOp 1
Largest Abelian subgroup C1 NOp 1
Largest concise Abelian subgroup C1 NOp 1
Standard orientation:

Center Number	Atomic Number	Atomic Type	Coordinates (Angstroms) X	Y	Z
1	6	0	3.356857	-1.122322	-0.051547
2	6	0	2.428862	-2.161680	0.021419
3	6	0	1.064728	-1.906222	0.019681
4	6	0	0.606053	-0.585647	-0.057075
5	6	0	1.526045	0.456188	-0.130916
6	6	0	2.897604	0.192914	-0.128512
7	1	0	4.416071	-1.347122	-0.048242
8	1	0	2.788382	-3.184085	0.080533
9	1	0	0.352177	-2.720185	0.076776
10	6	0	-0.877511	-0.284781	-0.059418
11	8	0	-1.230365	0.923243	-0.138469
12	8	0	-1.666575	-1.268807	0.018808
13	8	0	-3.905414	1.573734	-0.226678
14	1	0	-2.941953	1.373741	-0.204120
15	1	0	-4.295770	0.694001	-0.079736
16	8	0	-4.371464	-1.212788	0.179447

17	1	0	-3.382642	-1.217854	0.137916
18	1	0	-4.581672	-1.426397	1.094590
19	8	0	1.076295	1.811282	-0.210566
20	6	0	2.018650	2.666143	0.442182
21	1	0	2.823924	2.886169	-0.227163
22	1	0	1.535064	3.576285	0.729732
23	1	0	2.402082	2.175625	1.312396
24	1	0	3.536798	1.048953	-0.187906

Rotational constants (GHZ): 1.1907918 0.4308258 0.3199025

3-Metoxybenzoic Acid
Electronic Energy -688.197886
Free Energy -688.045444
Stoichiometry C8H12O5
Framework group C1[X(C8H12O5)]
Deg. of freedom 69
Full point group C1 NOp 1
Largest Abelian subgroup C1 NOp 1
Largest concise Abelian subgroup C1 NOp 1
Standard orientation:

Center Number	Atomic Number	Atomic Type	Coordinates (Angstroms) X	Y	Z
1	6	0	2.866096	1.041831	0.012478
2	6	0	2.452737	-0.285989	0.000460
3	6	0	1.095173	-0.594316	-0.010355
4	6	0	0.154104	0.425815	-0.009077
5	6	0	0.560668	1.760914	0.002843
6	6	0	1.911473	2.055007	0.013583
7	1	0	3.915829	1.299018	0.021118
8	1	0	0.792756	-1.632626	-0.019629
9	1	0	-0.178241	2.550579	0.003808
10	1	0	2.239578	3.087157	0.023156
11	6	0	-1.300643	0.119998	-0.019564
12	8	0	-2.152486	0.999534	-0.021852
13	8	0	-1.588182	-1.165219	-0.026965
14	1	0	-2.580684	-1.347771	-0.032691
15	8	0	-4.124803	-1.804859	-0.079357
16	1	0	-4.602747	-0.949403	-0.004263
17	1	0	-4.379113	-2.321232	0.695044
18	8	0	-4.913454	0.791679	0.118788
19	1	0	-5.327732	1.192456	-0.653922
20	1	0	-3.954961	0.965612	0.028964
21	8	0	3.304131	-1.352701	-0.001717
22	6	0	4.707043	-1.095743	0.008620
23	1	0	5.187282	-2.071493	0.004451
24	1	0	5.008633	-0.535938	-0.879552
25	1	0	4.997853	-0.548916	0.908410

Rotational constants (GHZ): 1.7374381 0.3044498 0.2598210

3-Metoxybenzoate
Electronic Energy -687.739805
Free Energy -687.601866
Stoichiometry C8H11O5(1-)
Framework group C1[X(C8H11O5)]
Deg. of freedom 66
Full point group C1 NOp 1
Largest Abelian subgroup C1 NOp 1
Largest concise Abelian subgroup C1 NOp 1
Standard orientation:

Center Number	Atomic Number	Atomic Type	Coordinates (Angstroms) X	Y	Z
1	6	0	-2.833574	1.042319	-0.015976
2	6	0	-1.866749	2.043160	-0.063083
3	6	0	-0.517837	1.733534	-0.073039
4	6	0	-0.115280	0.397311	-0.036606
5	6	0	-1.072407	-0.605631	0.009269
6	6	0	-2.427993	-0.286904	0.019828

7	1	0	-3.880972	1.309078	-0.008466
8	1	0	-2.183790	3.078930	-0.091827
9	1	0	0.225776	2.517751	-0.109425
10	1	0	-0.775314	-1.645832	0.037338
11	6	0	1.354057	0.037150	-0.045506
12	8	0	1.658866	-1.182965	-0.025666
13	8	0	2.183132	0.986541	-0.071596
14	8	0	4.296716	-1.926003	-0.123474
15	1	0	3.340590	-1.696350	-0.097356
16	1	0	4.722473	-1.056039	-0.024445
17	8	0	4.867528	0.830131	0.127625
18	1	0	3.880626	0.881570	0.078127
19	1	0	5.098226	1.134246	1.012298
20	8	0	-3.289981	-1.348480	0.067082
21	6	0	-4.689331	-1.078319	0.070850
22	1	0	-4.989495	-0.551438	-0.837983
23	1	0	-5.179934	-2.048426	0.107132
24	1	0	-4.973581	-0.492994	0.948411

Rotational constants (GHZ): 1.7267938 0.3052022 0.2603155

4-Metoxybenzoic Acid
Electronic Energy -688.200879
Free Energy -688.048720
Stoichiometry C8H12O5
Framework group C1[X(C8H12O5)]
Deg. of freedom 69
Full point group C1 NOp 1
Largest Abelian subgroup C1 NOp 1
Largest concise Abelian subgroup C1 NOp 1
Standard orientation:

Center Number	Atomic Number	Atomic Type	Coordinates (Angstroms) X	Y	Z
1	6	0	-2.844676	0.234220	0.006450
2	6	0	-2.097906	1.415343	0.013691
3	6	0	-0.721183	1.360674	0.002968
4	6	0	-0.060793	0.127361	-0.014491
5	6	0	-0.815162	-1.042177	-0.021632
6	6	0	-2.200041	-1.001432	-0.011770
7	1	0	-2.618558	2.364721	0.027815
8	1	0	-0.151042	2.279805	0.008816
9	1	0	-0.312538	-2.000660	-0.035205
10	1	0	-2.761676	-1.924432	-0.018011
11	6	0	1.412320	0.035860	-0.023839
12	8	0	2.010830	-1.035062	-0.030481
13	8	0	2.031214	1.203008	-0.024914
14	1	0	3.033351	1.113010	-0.035161
15	8	0	4.656759	1.143694	-0.095385
16	1	0	4.887988	0.194376	0.009614
17	1	0	5.041434	1.599051	0.663007
18	8	0	4.721489	-1.564115	0.147815
19	1	0	5.015812	-2.061602	-0.623843
20	1	0	3.752139	-1.469822	0.050606
21	8	0	-4.192212	0.384373	0.018078
22	6	0	-5.008934	-0.787403	0.013937
23	1	0	-6.036111	-0.431107	0.025979
24	1	0	-4.836208	-1.378239	-0.887713
25	1	0	-4.820988	-1.394957	0.901310

Rotational constants (GHZ): 2.3158494 0.2713487 0.2435860

4-Metoxybenzoate
Electronic Energy -687.741383
Free Energy -687.603456
Stoichiometry C8H11O5(1-)
Framework group C1[X(C8H11O5)]
Deg. of freedom 66
Full point group C1 NOp 1
Largest Abelian subgroup C1 NOp 1
Largest concise Abelian subgroup C1 NOp 1
Standard orientation:

Center Number	Atomic Number	Atomic Type	X	Y	Z
1	6	0	-2.806789	0.279718	-0.000731
2	6	0	-2.011464	1.426517	-0.014764
3	6	0	-0.635644	1.311678	-0.016856
4	6	0	-0.018477	0.058012	-0.005136
5	6	0	-0.825838	-1.073895	0.008728
6	6	0	-2.211746	-0.978230	0.011056
7	1	0	-2.490831	2.397966	-0.023974
8	1	0	-0.026371	2.205981	-0.027909
9	1	0	-0.366125	-2.053646	0.017748
10	1	0	-2.808660	-1.879232	0.021809
11	6	0	1.481249	-0.065393	-0.007916
12	8	0	1.977103	-1.224023	0.003304
13	8	0	2.156599	1.001311	-0.021809
14	8	0	4.685677	-1.574652	-0.012338
15	1	0	3.705945	-1.476155	-0.006809
16	1	0	4.989388	-0.650386	-0.008683
17	8	0	4.838896	1.254309	-0.056321
18	1	0	3.854565	1.154893	-0.018953
19	1	0	5.084575	1.712583	0.754734
20	8	0	-4.153894	0.485991	0.000383
21	6	0	-5.011718	-0.653701	0.014407
22	1	0	-6.026267	-0.261928	0.013133
23	1	0	-4.856809	-1.270066	-0.873751
24	1	0	-4.851908	-1.251733	0.914157

Rotational constants (GHZ): 2.3408247 0.2714260 0.2437506

- **PBE1PBE 6-311G+(d,p) SMD**

H_2O
Electronic Energy -229.167204
Free Energy -229.128233
Stoichiometry H6O3
Framework group C1[X(H6O3)]
Deg. of freedom 21
Full point group C1 NOp 1
Largest Abelian subgroup C1 NOp 1
Largest concise Abelian subgroup C1 NOp 1
Standard orientation:

Center Number	Atomic Number	Atomic Type	X	Y	Z
1	8	0	-0.000003	0.922277	0.000702
2	1	0	0.000028	1.507483	0.766526
3	1	0	-0.000011	1.514752	-0.759559
4	8	0	-2.444359	-0.471786	-0.001733
5	1	0	-2.225815	-1.407838	0.008210
6	1	0	-1.581806	-0.018098	-0.000592
7	8	0	2.444358	-0.471782	-0.001728
8	1	0	2.225845	-1.407840	0.008306
9	1	0	1.581790	-0.018123	-0.000816

Rotational constants (GHZ): 16.5713145 2.4374528 2.1460873

OH^-
Electronic Energy -228.685636
Free Energy -228.660024
Stoichiometry H5O3(1-)
Framework group C1[X(H5O3)]
Deg. of freedom 18
Full point group C1 NOp 1
Largest Abelian subgroup C1 NOp 1
Largest concise Abelian subgroup C1 NOp 1
Standard orientation:

Center Atomic Atomic Coordinates (Angstroms)

Number	Number	Type	X	Y	Z
1	8	0	-0.000225	0.657416	0.000044
2	1	0	-0.000769	1.616660	-0.000144
3	8	0	-2.351148	-0.340430	0.086619
4	1	0	-2.355030	-0.801681	-0.754038
5	1	0	-1.410034	0.086466	0.103715
6	8	0	2.351414	-0.340291	-0.086624
7	1	0	1.410559	0.086532	-0.103892
8	1	0	2.354945	-0.801536	0.754044

Rotational constants (GHZ): 31.7538037 2.6124257 2.4454439

Benzoic Acid
Electronic Energy -573.261605
Free Energy -573.136881
Stoichiometry C7H10O4
Framework group C1[X(C7H10O4)]
Deg. of freedom 57
Full point group C1 NOp 1
Largest Abelian subgroup C1 NOp 1
Largest concise Abelian subgroup C1 NOp 1
Standard orientation:

Center Number	Atomic Number	Atomic Type	Coordinates (Angstroms) X	Y	Z
1	6	0	3.743038	0.080081	0.015083
2	6	0	3.084503	-1.146375	0.019221
3	6	0	1.696503	-1.191224	0.007455
4	6	0	0.961023	-0.003451	-0.007234
5	6	0	1.625454	1.225065	-0.010863
6	6	0	3.012577	1.265642	-0.000199
7	1	0	4.827837	0.112348	0.023827
8	1	0	3.653963	-2.069670	0.031356
9	1	0	1.183090	-2.145459	0.010422
10	1	0	1.048319	2.142919	-0.022390
11	1	0	3.525804	2.221385	-0.003771
12	6	0	-0.522466	-0.012711	-0.018447
13	8	0	-1.182524	1.021001	-0.021203
14	8	0	-1.054561	-1.216223	-0.026354
15	1	0	-2.064945	-1.192632	-0.039408
16	8	0	-3.653389	-1.330526	-0.102333
17	1	0	-3.946355	-0.397793	-0.005150
18	1	0	-3.986920	-1.788095	0.676415
19	8	0	-3.938111	1.360193	0.137892
20	1	0	-4.250614	1.798133	-0.659983
21	1	0	-2.965288	1.341136	0.054585

Rotational constants (GHZ): 2.5496120 0.4636900 0.3931802

Benzoate
Electronic Energy -572.804045
Free Energy -572.695194
Stoichiometry C7H9O4(1-)
Framework group C1[X(C7H9O4)]
Deg. of freedom 54
Full point group C1 NOp 1
Largest Abelian subgroup C1 NOp 1
Largest concise Abelian subgroup C1 NOp 1
Standard orientation:

Center Number	Atomic Number	Atomic Type	Coordinates (Angstroms) X	Y	Z
1	6	0	-3.720624	0.022668	0.007030
2	6	0	-3.005310	1.212486	-0.064270
3	6	0	-1.616519	1.190681	-0.069484
4	6	0	-0.927316	-0.016101	-0.004110
5	6	0	-1.649300	-1.203296	0.066191
6	6	0	-3.037897	-1.186655	0.072259
7	1	0	-4.805497	0.037672	0.011129
8	1	0	-3.533051	2.159168	-0.116394

9	1	0	-1.032571	2.100991	-0.124914
10	1	0	-1.089515	-2.129199	0.114788
11	1	0	-3.591161	-2.118436	0.127366
12	6	0	0.601606	-0.042828	-0.009109
13	8	0	1.137093	-1.165428	0.044420
14	8	0	1.164096	1.080327	-0.064987
15	8	0	3.948397	-1.470471	-0.129504
16	1	0	2.972676	-1.474610	-0.067280
17	1	0	4.141583	-0.521615	-0.159821
18	8	0	3.795460	1.450279	0.059800
19	1	0	2.815594	1.299870	-0.051435
20	1	0	3.893736	1.626762	0.997688

Rotational constants (GHZ): 2.4728657 0.4643442 0.3919859

4-Cyanobenzoic Acid

Electronic Energy -665.423268
Free Energy -665.302842
Stoichiometry C8H9NO4
Framework group C1[X(C8H9NO4)]
Deg. of freedom 60
Full point group C1 NOp 1
Largest Abelian subgroup C1 NOp 1
Largest concise Abelian subgroup C1 NOp 1
Standard orientation:

Center Number	Atomic Number	Atomic Type	Coordinates (Angstroms) X	Y	Z
1	6	0	3.000493	0.037899	0.000660
2	6	0	2.342699	-1.191710	-0.025493
3	6	0	0.959623	-1.229468	-0.025870
4	6	0	0.230936	-0.043774	-0.001014
5	6	0	0.889881	1.181565	0.024506
6	6	0	2.271556	1.227502	0.025871
7	1	0	2.918243	-2.107919	-0.045351
8	1	0	0.436518	-2.175488	-0.046170
9	1	0	0.302906	2.090231	0.043876
10	1	0	2.792226	2.175946	0.046338
11	6	0	-1.261291	-0.049941	-0.001330
12	8	0	-1.906496	0.983294	0.002598
13	8	0	-1.783347	-1.254597	-0.007073
14	1	0	-2.787218	-1.239316	-0.011243
15	8	0	-4.403093	-1.251510	-0.043120
16	1	0	-4.718237	-0.319178	-0.031168
17	1	0	-4.920201	-1.744273	0.598546
18	8	0	-4.620755	1.395754	0.046924
19	1	0	-4.945074	2.035091	-0.591284
20	1	0	-3.644247	1.436877	0.032740
21	6	0	4.433440	0.079717	0.001548
22	7	0	5.581514	0.113394	0.002261

Rotational constants (GHZ): 2.5600692 0.2780027 0.2509715

4-Cyanobenzoate

Electronic Energy -664.9679350
Free Energy -664.863002
Stoichiometry C8H8NO4(1-)
Framework group C1[X(C8H8NO4)]
Deg. of freedom 57
Full point group C1 NOp 1
Largest Abelian subgroup C1 NOp 1
Largest concise Abelian subgroup C1 NOp 1
Standard orientation:

Center Number	Atomic Number	Atomic Type	Coordinates (Angstroms) X	Y	Z
1	6	0	2.974013	-0.022523	0.003669
2	6	0	2.251819	-1.219868	-0.026369
3	6	0	0.867189	-1.176576	-0.031473
4	6	0	0.192894	0.045244	-0.007681
5	6	0	0.925788	1.232773	0.021943

6	6	0	2.310902	1.208849	0.028233
7	1	0	2.777489	-2.167742	-0.045201
8	1	0	0.299250	-2.099051	-0.054400
9	1	0	0.402461	2.181450	0.039987
10	1	0	2.881659	2.130161	0.051351
11	6	0	-1.321527	0.083228	-0.014212
12	8	0	-1.867475	1.214586	-0.003034
13	8	0	-1.919797	-1.024103	-0.030931
14	8	0	-4.611833	1.403280	-0.025872
15	1	0	-3.630838	1.371938	-0.017726
16	1	0	-4.843151	0.458930	-0.019189
17	8	0	-4.593490	-1.433601	-0.035586
18	1	0	-3.619356	-1.275711	-0.009928
19	1	0	-4.805746	-1.815701	0.820984
20	6	0	4.397921	-0.058724	0.009276
21	7	0	5.555000	-0.088570	0.013886

Rotational constants (GHZ): 2.5068609 0.2779252 0.2503766

2,6-Dimethylbenzoic Acid
Electronic Energy -651.809581
Free Energy -651.612299
Stoichiometry C9H14O4
Framework group C1[X(C9H14O4)]
Deg. of freedom 75
Full point group C1 NOp 1
Largest Abelian subgroup C1 NOp 1
Largest concise Abelian subgroup C1 NOp 1
Standard orientation:

Center Number	Atomic Number	Atomic Type	Coordinates (Angstroms) X	Y	Z
1	6	0	-3.601314	0.109154	0.047875
2	6	0	-2.952020	-1.097216	0.246430
3	6	0	-1.563559	-1.180047	0.208471
4	6	0	-0.832393	-0.002136	-0.012359
5	6	0	-1.476561	1.231992	-0.195461
6	6	0	-2.867034	1.261359	-0.176059
7	1	0	-4.683960	0.152000	0.070420
8	1	0	-3.527857	-1.996182	0.433285
9	1	0	-3.376242	2.204812	-0.334710
10	6	0	0.660407	-0.034545	-0.030237
11	8	0	1.340743	0.770892	0.582588
12	8	0	1.168255	-0.996973	-0.772151
13	1	0	2.167425	-1.007240	-0.744684
14	8	0	3.816042	-1.091702	-0.714449
15	1	0	4.136794	-0.342385	-0.163994
16	1	0	4.334446	-1.101575	-1.522481
17	8	0	4.044399	1.085682	0.801592
18	1	0	4.348478	1.203630	1.704210
19	1	0	3.065537	1.105340	0.819445
20	6	0	-0.912054	-2.521346	0.431335
21	1	0	-0.509944	-2.923478	-0.499160
22	1	0	-0.081343	-2.462128	1.136056
23	1	0	-1.641323	-3.228394	0.827483
24	6	0	-0.723139	2.516270	-0.427848
25	1	0	-0.222740	2.847318	0.482611
26	1	0	0.050628	2.405480	-1.189330
27	1	0	-1.409408	3.298698	-0.752673

Rotational constants (GHZ): 1.2645324 0.4352665 0.3465483

2,6-Dimethylbenzoate
Electronic Energy -664.9679350
Free Energy -651.192205
Stoichiometry C9H13O4(1-)
Framework group C1[X(C9H13O4)]
Deg. of freedom 72
Full point group C1 NOp 1
Largest Abelian subgroup C1 NOp 1
Largest concise Abelian subgroup C1 NOp 1
Standard orientation:

Center Number	Atomic Number	Atomic Type	X	Y	Z
1	6	0	3.589988	-0.015340	0.027595
2	6	0	2.885682	-1.188575	-0.196226
3	6	0	1.492922	-1.195847	-0.209812
4	6	0	0.802254	0.006149	-0.013974
5	6	0	1.505403	1.197542	0.200838
6	6	0	2.897650	1.168935	0.229438
7	1	0	4.674714	-0.023741	0.043694
8	1	0	3.421265	-2.118775	-0.358835
9	1	0	3.442481	2.091134	0.406337
10	6	0	-0.722943	0.019924	-0.039801
11	8	0	-1.259157	0.720968	-0.917483
12	8	0	-1.287830	-0.683117	0.836528
13	8	0	-4.083411	0.777626	-1.243635
14	1	0	-3.107616	0.822589	-1.223633
15	1	0	-4.272274	0.195198	-0.492734
16	8	0	-3.921998	-0.821060	1.203958
17	1	0	-2.941330	-0.804685	1.024660
18	1	0	-4.032583	-0.213224	1.937879
19	6	0	0.775459	2.499936	0.401334
20	1	0	0.186432	2.747570	-0.482369
21	1	0	0.069487	2.431596	1.232851
22	1	0	1.478363	3.309322	0.611644
23	6	0	0.749309	-2.485618	-0.438899
24	1	0	0.132727	-2.729957	0.426776
25	1	0	0.067641	-2.400779	-1.288807
26	1	0	1.445514	-3.304213	-0.635363

Rotational constants (GHZ): 1.2552577 0.4228183 0.3564447

4-Bromobenzoic Acid
Electronic Energy -3146.484016
Free Energy -3146.372666
Stoichiometry C7H9BrO4
Framework group C1[X(C7H9BrO4)]
Deg. of freedom 57
Full point group C1 NOp 1
Largest Abelian subgroup C1 NOp 1
Largest concise Abelian subgroup C1 NOp 1
Standard orientation:

Center Number	Atomic Number	Atomic Type	X	Y	Z
1	6	0	-2.107850	-0.009043	-0.000188
2	6	0	-1.431136	1.207466	0.006444
3	6	0	-0.042593	1.231639	-0.001077
4	6	0	0.675156	0.033048	-0.013999
5	6	0	-0.007485	-1.185433	-0.020140
6	6	0	-1.395085	-1.205347	-0.013717
7	1	0	-1.986815	2.139137	0.017212
8	1	0	0.484965	2.178120	0.003833
9	1	0	0.555947	-2.111778	-0.030274
10	1	0	-1.922477	-2.153338	-0.019232
11	6	0	2.158645	0.020213	-0.020690
12	8	0	2.803239	-1.023215	-0.021847
13	8	0	2.708629	1.215669	-0.026497
14	1	0	3.718585	1.177034	-0.036483
15	8	0	5.309091	1.291274	-0.094515
16	1	0	5.587834	0.354247	0.003188
17	1	0	5.647022	1.743550	0.685427
18	8	0	5.552970	-1.403472	0.145505
19	1	0	5.861348	-1.845737	-0.651588
20	1	0	4.580797	-1.369897	0.059243
21	35	0	-4.017616	-0.037390	0.009363

Rotational constants (GHZ): 2.5449074 0.1836292 0.1714338

4-Bromobenzoate
Electronic Energy -3146.027374

Free Energy -3145.930710
Stoichiometry C7H8BrO4(1-)
Framework group C1[X(C7H8BrO4)]
Deg. of freedom 54
Full point group C1 NOp 1
Largest Abelian subgroup C1 NOp 1
Largest concise Abelian subgroup C1 NOp 1
Standard orientation:

Center Number	Atomic Number	Atomic Type	Coordinates (Angstroms) X	Y	Z
1	6	0	2.066907	-0.001100	0.000151
2	6	0	1.364740	-1.199175	-0.031915
3	6	0	-0.023760	-1.160738	-0.039748
4	6	0	-0.708946	0.054236	-0.016294
5	6	0	0.023783	1.240956	0.015647
6	6	0	1.412648	1.224028	0.024302
7	1	0	1.887166	-2.148468	-0.050278
8	1	0	-0.581929	-2.089730	-0.064624
9	1	0	-0.497860	2.191138	0.033983
10	1	0	1.972385	2.151615	0.049196
11	6	0	-2.218657	0.085458	-0.024947
12	8	0	-2.774307	1.213916	-0.007329
13	8	0	-2.816634	-1.023822	-0.049977
14	8	0	-5.510987	1.391830	-0.016355
15	1	0	-4.528994	1.360261	-0.013669
16	1	0	-5.742074	0.447580	-0.005177
17	8	0	-5.480765	-1.445858	-0.017703
18	1	0	-4.506685	-1.279969	-0.005846
19	1	0	-5.678919	-1.824416	0.843755
20	35	0	3.966805	-0.038815	0.010868

Rotational constants (GHZ): 2.5105257 0.1854850 0.1728207

2-Bromobenzoic Acid
Electronic Energy -3146.474323
Free Energy -3146.363860
Stoichiometry C7H9BrO4
Framework group C1[X(C7H9BrO4)]
Deg. of freedom 57
Full point group C1 NOp 1
Largest Abelian subgroup C1 NOp 1
Largest concise Abelian subgroup C1 NOp 1
Standard orientation:

Center Number	Atomic Number	Atomic Type	Coordinates (Angstroms) X	Y	Z
1	6	0	-3.437839	1.221238	0.019920
2	6	0	-2.619412	2.347309	0.015308
3	6	0	-1.238123	2.203856	0.002846
4	6	0	-0.670133	0.927417	-0.003747
5	6	0	-1.494680	-0.199894	0.001390
6	6	0	-2.874524	-0.052334	0.012720
7	1	0	-3.058695	3.339227	0.021149
8	1	0	-0.600317	3.079844	-0.000995
9	1	0	-3.512350	-0.929846	0.015967
10	6	0	0.800952	0.735786	-0.015433
11	8	0	1.315072	-0.377725	-0.011149
12	8	0	1.490983	1.856150	-0.031895
13	1	0	2.488867	1.695974	-0.045062
14	8	0	4.081284	1.617234	-0.109387
15	1	0	4.245472	0.654129	-0.005770
16	1	0	4.474606	2.030816	0.666054
17	8	0	3.999618	-1.085547	0.149552
18	1	0	4.249020	-1.567227	-0.645330
19	1	0	3.038218	-0.935609	0.066382
20	1	0	-4.501789	1.334490	0.029057
21	35	0	-0.706175	-1.939513	-0.007899

Rotational constants (GHZ): 0.8328721 0.4501754 0.2927165

2-Bromobenzoate
Electronic Energy -3146.02019
Free Energy -3145.924772
Stoichiometry C7H8BrO4(1-)
Framework group C1[X(C7H8BrO4)]
Deg. of freedom 54
Full point group C1 NOp 1
Largest Abelian subgroup C1 NOp 1
Largest concise Abelian subgroup C1 NOp 1
Standard orientation:

Center Number	Atomic Number	Atomic Type	Coordinates (Angstroms) X	Y	Z
1	6	0	3.431209	1.203004	0.030857
2	6	0	2.849403	-0.061686	0.016450
3	6	0	1.465389	-0.191353	0.000618
4	6	0	0.646936	0.940075	-0.001171
5	6	0	1.239174	2.204663	0.013123
6	6	0	2.622887	2.336520	0.029220
7	1	0	3.475682	-0.948181	0.017251
8	1	0	0.607549	3.086071	0.011721
9	1	0	3.072099	3.324533	0.040409
10	6	0	-0.856689	0.799775	-0.017723
11	8	0	-1.539781	1.857194	-0.022725
12	8	0	-1.328658	-0.369924	-0.025741
13	8	0	-4.276663	1.724907	-0.040781
14	1	0	-3.297087	1.803888	-0.036286
15	1	0	-4.399936	0.760932	-0.015559
16	8	0	-3.926324	-1.090754	0.001669
17	1	0	-2.977206	-0.814777	0.014324
18	1	0	-4.084541	-1.476386	0.868242
19	1	0	4.496410	1.303479	0.043097
20	35	0	0.665388	-1.925627	-0.019166

Rotational constants (GHZ): 0.8368558 0.4513107 0.2934844

2-Chlorobenzoic Acid
Electronic Energy -1032.715735
Free Energy -1032.603103
Stoichiometry C7H9ClO4
Framework group C1[X(C7H9ClO4)]
Deg. of freedom 57
Full point group C1 NOp 1
Largest Abelian subgroup C1 NOp 1
Largest concise Abelian subgroup C1 NOp 1
Standard orientation:

Center Number	Atomic Number	Atomic Type	Coordinates (Angstroms) X	Y	Z
1	6	0	-3.505240	-0.910756	0.141919
2	6	0	-3.020906	0.371280	-0.069580
3	6	0	-1.652989	0.593258	-0.100741
4	6	0	-0.752171	-0.458175	0.065903
5	6	0	-1.260936	-1.743885	0.249549
6	6	0	-2.625242	-1.972354	0.299750
7	1	0	-4.575039	-1.076257	0.173057
8	1	0	-3.702141	1.198911	-0.217039
9	1	0	-0.564479	-2.564256	0.367760
10	1	0	-2.999344	-2.975770	0.458166
11	6	0	0.733839	-0.319815	0.059097
12	8	0	1.438634	-1.123261	-0.534299
13	8	0	1.194721	0.680882	0.767809
14	1	0	2.205070	0.753644	0.742222
15	8	0	3.775044	1.014321	0.760119
16	1	0	4.132704	0.245012	0.263041
17	1	0	4.142743	0.952420	1.649999
18	8	0	4.220231	-1.238361	-0.692042
19	1	0	4.520846	-1.123774	-1.600938
20	1	0	3.244784	-1.271717	-0.736220
21	17	0	-1.113666	2.225637	-0.434596

Rotational constants (GHZ): 1.2223449 0.4288751 0.3372817

2-Chlorobenzoate
Electronic Energy -1032.261545
Free Energy -1032.163949
Stoichiometry C7H8ClO4(1-)
Framework group C1[X(C7H8ClO4)]
Deg. of freedom 54
Full point group C1 NOp 1
Largest Abelian subgroup C1 NOp 1
Largest concise Abelian subgroup C1 NOp 1
Standard orientation:

Center Number	Atomic Number	Atomic Type	Coordinates (Angstroms) X	Y	Z
1	6	0	3.552936	-0.699386	0.086638
2	6	0	2.763100	-1.844749	0.089452
3	6	0	1.379097	-1.734460	0.040154
4	6	0	0.762368	-0.483620	-0.011999
5	6	0	1.574116	0.646401	-0.014725
6	6	0	2.959059	0.555888	0.033993
7	1	0	4.634256	-0.776769	0.125349
8	1	0	3.224670	-2.825632	0.130418
9	1	0	0.760298	-2.626516	0.042819
10	1	0	3.562694	1.456463	0.030954
11	6	0	-0.746570	-0.388321	-0.060357
12	8	0	-1.276383	-0.400996	-1.196468
13	8	0	-1.338423	-0.323582	1.045878
14	8	0	-4.033980	-0.335284	-1.407594
15	1	0	-3.054769	-0.361689	-1.375421
16	1	0	-4.263687	-0.244776	-0.467040
17	8	0	-4.023942	-0.127203	1.421335
18	1	0	-3.049375	-0.170976	1.280621
19	1	0	-4.188251	0.764802	1.741165
20	17	0	0.840668	2.233595	-0.081941

Rotational constants (GHZ): 1.2423611 0.4124790 0.3587944

3-Chlorobenzoic Acid
Electronic Energy -1032.723914
Free Energy -1032.610875
Stoichiometry C7H9ClO4
Framework group C1[X(C7H9ClO4)]
Deg. of freedom 57
Full point group C1 NOp 1
Largest Abelian subgroup C1 NOp 1
Largest concise Abelian subgroup C1 NOp 1
Standard orientation:

Center Number	Atomic Number	Atomic Type	Coordinates (Angstroms) X	Y	Z
1	6	0	2.924424	1.163945	0.011212
2	6	0	2.522901	-0.162384	0.001551
3	6	0	1.187636	-0.519560	-0.006937
4	6	0	0.225296	0.487844	-0.005389
5	6	0	0.610212	1.825463	0.004260
6	6	0	1.955336	2.158003	0.012485
7	1	0	3.976439	1.417957	0.017702
8	1	0	0.896484	-1.560277	-0.014676
9	1	0	-0.145286	2.599725	0.005323
10	1	0	2.257867	3.197408	0.020058
11	6	0	-1.224833	0.155328	-0.013479
12	8	0	-2.090053	1.020264	-0.012029
13	8	0	-1.483656	-1.134564	-0.022958
14	1	0	-2.473351	-1.341893	-0.028541
15	8	0	-3.998053	-1.825490	-0.079580
16	1	0	-4.495013	-0.980167	-0.009858
17	1	0	-4.246391	-2.345569	0.694353
18	8	0	-4.853942	0.749406	0.107000
19	1	0	-5.272794	1.133628	-0.671683
20	1	0	-3.901059	0.952592	0.025418

21	17	0	3.741347	-1.423657	0.000013

Rotational constants (GHZ): 1.5691885 0.3154463 0.2629838

3-Chlorobenzoate
Electronic Energy -1032.267777
Free Energy -1032.168879
Stoichiometry C7H8ClO4(1-)
Framework group C1[X(C7H8ClO4)]
Deg. of freedom 54
Full point group C1 NOp 1
Largest Abelian subgroup C1 NOp 1
Largest concise Abelian subgroup C1 NOp 1
Standard orientation:

Center Number	Atomic Number	Atomic Type	Coordinates (Angstroms) X	Y	Z
1	6	0	-2.896250	1.164854	-0.013166
2	6	0	-1.914540	2.148940	-0.053133
3	6	0	-0.568892	1.803199	-0.062541
4	6	0	-0.185645	0.462565	-0.033287
5	6	0	-1.163775	-0.531346	0.005765
6	6	0	-2.500363	-0.165871	0.015643
7	1	0	-3.947994	1.427295	-0.004817
8	1	0	-2.209070	3.192824	-0.076493
9	1	0	0.192497	2.573786	-0.093108
10	1	0	-0.873950	-1.575029	0.028373
11	6	0	1.276483	0.074993	-0.041524
12	8	0	1.549314	-1.152011	-0.023746
13	8	0	2.117756	1.012365	-0.064990
14	8	0	4.173188	-1.957448	-0.126935
15	1	0	3.224848	-1.704023	-0.098469
16	1	0	4.613525	-1.095589	-0.032590
17	8	0	4.804401	0.793105	0.112221
18	1	0	3.820122	0.861775	0.067577
19	1	0	5.030998	1.061287	1.007523
20	17	0	-3.722962	-1.414965	0.066063

Rotational constants (GHZ): 1.5550826 0.3164413 0.2634411

4-Chlorobenzoic Acid
Electronic Energy -1032.724826
Free Energy -1032.611766
Stoichiometry C7H9ClO4
Framework group C1[X(C7H9ClO4)]
Deg. of freedom 57
Full point group C1 NOp 1
Largest Abelian subgroup C1 NOp 1
Largest concise Abelian subgroup C1 NOp 1
Standard orientation:

Center Number	Atomic Number	Atomic Type	Coordinates (Angstroms) X	Y	Z
1	6	0	2.841072	0.026948	0.004223
2	6	0	2.190205	-1.196515	0.014387
3	6	0	0.805076	-1.217076	0.005009
4	6	0	0.084063	-0.025153	-0.012962
5	6	0	0.761203	1.191933	-0.022515
6	6	0	2.145111	1.225851	-0.014601
7	1	0	2.754071	-2.119631	0.029051
8	1	0	0.287524	-2.166396	0.012481
9	1	0	0.201268	2.117585	-0.036494
10	1	0	2.673671	2.169725	-0.022735
11	6	0	-1.401080	-0.018826	-0.021376
12	8	0	-2.049705	1.019218	-0.022807
13	8	0	-1.947534	-1.216532	-0.027688
14	1	0	-2.956532	-1.186832	-0.038097
15	8	0	-4.558878	-1.302047	-0.101331
16	1	0	-4.846649	-0.367491	-0.000717
17	1	0	-4.920584	-1.777055	0.656469
18	8	0	-4.799305	1.394374	0.149628

19	1	0	-5.119386	1.876074	-0.621864
20	1	0	-3.826062	1.365894	0.060301
21	17	0	4.590715	0.059120	0.015658

Rotational constants (GHZ): 2.5425254 0.2624028 0.2381627

4-Chlorobenzoate
Electronic Energy -1032.268053
Free Energy -1032.169250
Stoichiometry C7H8ClO4(1-)
Framework group C1[X(C7H8ClO4)]
Deg. of freedom 54
Full point group C1 NOp 1
Largest Abelian subgroup C1 NOp 1
Largest concise Abelian subgroup C1 NOp 1
Standard orientation:

Center Number	Atomic Number	Atomic Type	Coordinates (Angstroms) X	Y	Z
1	6	0	2.810550	-0.020753	-0.001734
2	6	0	2.106204	-1.213631	-0.001527
3	6	0	0.720092	-1.170186	0.003746
4	6	0	0.042751	0.046488	0.008707
5	6	0	0.778393	1.228832	0.008541
6	6	0	2.164897	1.204884	0.003322
7	1	0	2.628731	-2.161286	-0.005603
8	1	0	0.158968	-2.095005	0.003580
9	1	0	0.262185	2.179492	0.012176
10	1	0	2.733140	2.125827	0.002986
11	6	0	-1.468317	0.084420	0.012463
12	8	0	-2.022280	1.213448	0.013300
13	8	0	-2.075010	-1.020038	0.013964
14	8	0	-4.758419	1.405075	-0.007489
15	1	0	-3.775605	1.371047	0.003192
16	1	0	-5.000805	0.462617	-0.024689
17	8	0	-4.740769	-1.423288	-0.106855
18	1	0	-3.765653	-1.271772	-0.038340
19	1	0	-4.985175	-1.905189	0.690827
20	17	0	4.564036	-0.062802	-0.008741

Rotational constants (GHZ): 2.5195961 0.2631042 0.2383770

2-Metoxybenzoic Acid
Electronic Energy -687.688272
Free Energy -687.534202
Stoichiometry C8H12O5
Framework group C1[X(C8H12O5)]
Deg. of freedom 69
Full point group C1 NOp 1
Largest Abelian subgroup C1 NOp 1
Largest concise Abelian subgroup C1 NOp 1
Standard orientation:

Center Number	Atomic Number	Atomic Type	Coordinates (Angstroms) X	Y	Z
1	6	0	3.298824	-1.276166	-0.135322
2	6	0	2.956147	0.060917	-0.004012
3	6	0	1.616210	0.444581	0.043790
4	6	0	0.613859	-0.541869	-0.037875
5	6	0	0.990364	-1.880467	-0.149719
6	6	0	2.319978	-2.257360	-0.208751
7	1	0	4.346977	-1.548001	-0.175820
8	1	0	3.737430	0.803531	0.065289
9	1	0	0.210419	-2.628881	-0.203913
10	1	0	2.588519	-3.300931	-0.310079
11	6	0	-0.845499	-0.267229	-0.002556
12	8	0	-1.640428	-1.103977	0.412086
13	8	0	-1.223877	0.900456	-0.475755
14	1	0	-2.221523	1.031913	-0.423380

15	8	0	-3.793570	1.429225	-0.381437
16	1	0	-4.218995	0.574247	-0.149635
17	1	0	-4.128731	1.666527	-1.254422
18	8	0	-4.411105	-1.121842	0.331869
19	1	0	-4.778835	-1.256111	1.212910
20	1	0	-3.440639	-1.193931	0.432418
21	8	0	1.235096	1.734189	0.200468
22	6	0	2.241185	2.738716	0.330157
23	1	0	1.702950	3.675581	0.452731
24	1	0	2.864479	2.556482	1.207824
25	1	0	2.862622	2.788429	-0.566031

Rotational constants (GHZ): 1.1793548 0.4201408 0.3171942

2-Metoxybenzoate

Electronic Energy -687.230924

Free Energy -687.090877

Stoichiometry C8H11O5(1-)

Framework group C1[X(C8H11O5)]

Deg. of freedom 66

Full point group C1 NOp 1

Largest Abelian subgroup C1 NOp 1

Largest concise Abelian subgroup C1 NOp 1

Standard orientation:

Center Number	Atomic Number	Atomic Type	Coordinates (Angstroms) X	Y	Z
1	6	0	3.322062	-1.166098	-0.136845
2	6	0	2.385440	-2.175424	-0.294385
3	6	0	1.034743	-1.854430	-0.258276
4	6	0	0.603682	-0.543193	-0.092748
5	6	0	1.562846	0.467969	0.073051
6	6	0	2.918406	0.149960	0.054482
7	1	0	4.381097	-1.395050	-0.154647
8	1	0	2.698726	-3.201789	-0.439303
9	1	0	0.290049	-2.633241	-0.370648
10	1	0	3.664539	0.919365	0.191537
11	6	0	-0.880177	-0.265146	-0.111587
12	8	0	-1.302792	0.666244	-0.841800
13	8	0	-1.605206	-1.023445	0.588038
14	8	0	-3.991186	1.224476	-0.919986
15	1	0	-3.023053	1.052245	-0.912227
16	1	0	-4.332348	0.536095	-0.322423
17	8	0	-4.293322	-0.981123	0.832006
18	1	0	-3.307360	-0.979853	0.754126
19	1	0	-4.479386	-0.770184	1.753556
20	8	0	1.103160	1.734124	0.282174
21	6	0	2.052407	2.776939	0.490021
22	1	0	2.699786	2.898739	-0.381162
23	1	0	1.467958	3.682458	0.636943
24	1	0	2.658314	2.585549	1.378520

Rotational constants (GHZ): 1.1675696 0.4147293 0.3280496

3-Metoxybenzoic Acid

Electronic Energy -687.693464

Free Energy -687.539708

Stoichiometry C8H12O5

Framework group C1[X(C8H12O5)]

Deg. of freedom 69

Full point group C1 NOp 1

Largest Abelian subgroup C1 NOp 1

Largest concise Abelian subgroup C1 NOp 1

Standard orientation:

Center Number	Atomic Number	Atomic Type	Coordinates (Angstroms) X	Y	Z
1	6	0	2.866096	1.041831	0.012478
2	6	0	2.452737	-0.285989	0.000460
3	6	0	1.095173	-0.594316	-0.010355

4	6	0	0.154104	0.425815	-0.009077
5	6	0	0.560668	1.760914	0.002843
6	6	0	1.911473	2.055007	0.013583
7	1	0	3.915829	1.299018	0.021118
8	1	0	0.792756	-1.632626	-0.019629
9	1	0	-0.178241	2.550579	0.003808
10	1	0	2.239578	3.087157	0.023156
11	6	0	-1.300643	0.119998	-0.019564
12	8	0	-2.152486	0.999534	-0.021852
13	8	0	-1.588182	-1.165219	-0.026965
14	1	0	-2.580684	-1.347771	-0.032691
15	8	0	-4.124803	-1.804859	-0.079357
16	1	0	-4.602747	-0.949403	-0.004263
17	1	0	-4.379113	-2.321232	0.695044
18	8	0	-4.913454	0.791679	0.118788
19	1	0	-5.327732	1.192456	-0.653922
20	1	0	-3.954961	0.965612	0.028964
21	8	0	3.304131	-1.352701	-0.001717
22	6	0	4.707043	-1.095743	0.008620
23	1	0	5.187282	-2.071493	0.004451
24	1	0	5.008633	-0.535938	-0.879552
25	1	0	4.997853	-0.548916	0.908410

Rotational constants (GHZ): 1.7374381 0.3044498 0.2598210

3-Metoxybenzoate
Electronic Energy -687.236132
Free Energy -687.096984
Stoichiometry C8H11O5(1-)
Framework group C1[X(C8H11O5)]
Deg. of freedom 66
Full point group C1 NOp 1
Largest Abelian subgroup C1 NOp 1
Largest concise Abelian subgroup C1 NOp 1
Standard orientation:

Center Number	Atomic Number	Atomic Type	Coordinates (Angstroms) X	Y	Z
1	6	0	-2.833574	1.042319	-0.015976
2	6	0	-1.866749	2.043160	-0.063083
3	6	0	-0.517837	1.733534	-0.073039
4	6	0	-0.115280	0.397311	-0.036606
5	6	0	-1.072407	-0.605631	0.009269
6	6	0	-2.427993	-0.286904	0.019828
7	1	0	-3.880972	1.309078	-0.008466
8	1	0	-2.183790	3.078930	-0.091827
9	1	0	0.225776	2.517751	-0.109425
10	1	0	-0.775314	-1.645832	0.037338
11	6	0	1.354057	0.037150	-0.045506
12	8	0	1.658866	-1.182965	-0.025666
13	8	0	2.183132	0.986541	-0.071596
14	8	0	4.296716	-1.926003	-0.123474
15	1	0	3.340590	-1.696350	-0.097356
16	1	0	4.722473	-1.056039	-0.024445
17	8	0	4.867528	0.830131	0.127625
18	1	0	3.880626	0.881570	0.078127
19	1	0	5.098226	1.134246	1.012298
20	8	0	-3.289981	-1.348480	0.067082
21	6	0	-4.689331	-1.078319	0.070850
22	1	0	-4.989495	-0.551438	-0.837983
23	1	0	-5.179934	-2.048426	0.107132
24	1	0	-4.973581	-0.492994	0.948411

Rotational constants (GHZ): 1.7267938 0.3052022 0.2603155

4-Metoxybenzoic Acid
Electronic Energy -687.696458
Free Energy -687.542941
Stoichiometry C8H12O5
Framework group C1[X(C8H12O5)]
Deg. of freedom 69
Full point group C1 NOp 1

Largest Abelian subgroup C1 NOp 1
Largest concise Abelian subgroup C1 NOp 1
Standard orientation:

Center Number	Atomic Number	Atomic Type	Coordinates (Angstroms) X	Y	Z
1	6	0	-2.844676	0.234220	0.006450
2	6	0	-2.097906	1.415343	0.013691
3	6	0	-0.721183	1.360674	0.002968
4	6	0	-0.060793	0.127361	-0.014491
5	6	0	-0.815162	-1.042177	-0.021632
6	6	0	-2.200041	-1.001432	-0.011770
7	1	0	-2.618558	2.364721	0.027815
8	1	0	-0.151042	2.279805	0.008816
9	1	0	-0.312538	-2.000660	-0.035205
10	1	0	-2.761676	-1.924432	-0.018011
11	6	0	1.412320	0.035860	-0.023839
12	8	0	2.010830	-1.035062	-0.030481
13	8	0	2.031214	1.203008	-0.024914
14	1	0	3.033351	1.113010	-0.035161
15	8	0	4.656759	1.143694	-0.095385
16	1	0	4.887988	0.194376	0.009614
17	1	0	5.041434	1.599051	0.663007
18	8	0	4.721489	-1.564115	0.147815
19	1	0	5.015812	-2.061602	-0.623843
20	1	0	3.752139	-1.469822	0.050606
21	8	0	-4.192212	0.384373	0.018078
22	6	0	-5.008934	-0.787403	0.013937
23	1	0	-6.036111	-0.431107	0.025979
24	1	0	-4.836208	-1.378239	-0.887713
25	1	0	-4.820988	-1.394957	0.901310

Rotational constants (GHZ): 2.3158494 0.2713487 0.2435860

4-Metoxybenzoate
Electronic Energy -687.237711
Free Energy -687.098629
Stoichiometry C8H11O5(1-)
Framework group C1[X(C8H11O5)]
Deg. of freedom 66
Full point group C1 NOp 1
Largest Abelian subgroup C1 NOp 1
Largest concise Abelian subgroup C1 NOp 1
Standard orientation:

Center Number	Atomic Number	Atomic Type	Coordinates (Angstroms) X	Y	Z
1	6	0	-2.806789	0.279718	-0.000731
2	6	0	-2.011464	1.426517	-0.014764
3	6	0	-0.635644	1.311678	-0.016856
4	6	0	-0.018477	0.058012	-0.005136
5	6	0	-0.825838	-1.073895	0.008728
6	6	0	-2.211746	-0.978230	0.011056
7	1	0	-2.490831	2.397966	-0.023974
8	1	0	-0.026371	2.205981	-0.027909
9	1	0	-0.366125	-2.053646	0.017748
10	1	0	-2.808660	-1.879232	0.021809
11	6	0	1.481249	-0.065393	-0.007916
12	8	0	1.977103	-1.224023	0.003304
13	8	0	2.156599	1.001311	-0.021809
14	8	0	4.685677	-1.574652	-0.012338
15	1	0	3.705945	-1.476155	-0.006809
16	1	0	4.989388	-0.650386	-0.008683
17	8	0	4.838896	1.254309	-0.056321
18	1	0	3.854565	1.154893	-0.018953
19	1	0	5.084575	1.712583	0.754734
20	8	0	-4.153894	0.485991	0.000383
21	6	0	-5.011718	-0.653701	0.014407
22	1	0	-6.026267	-0.261928	0.013133
23	1	0	-4.856809	-1.270066	-0.873751
24	1	0	-4.851908	-1.251733	0.914157

Rotational constants (GHZ): 2.3408247 0.2714260 0.2437506

- **PBEPBE 6-311G+(d,p) SMD**

H_2O
Electronic Energy -229.168244
Free Energy -229.131269
Stoichiometry H6O3
Framework group C1[X(H6O3)]
Deg. of freedom 21
Full point group C1 NOp 1
Largest Abelian subgroup C1 NOp 1
Largest concise Abelian subgroup C1 NOp 1
Standard orientation:

Center Number	Atomic Number	Atomic Type	Coordinates (Angstroms) X	Y	Z
1	8	0	0.000013	0.895722	0.009934
2	1	0	0.000292	1.426273	0.827572
3	1	0	-0.000257	1.559047	-0.704711
4	8	0	-2.470964	-0.458966	-0.027023
5	1	0	-2.259331	-1.395136	0.126713
6	1	0	-1.591336	-0.008585	-0.011517
7	8	0	2.470980	-0.458960	-0.026798
8	1	0	2.259031	-1.395438	0.124607
9	1	0	1.591372	-0.008527	-0.011568

Rotational constants (GHZ): 17.2835749 2.3843670 2.1171235

OH^-
Electronic energy -228.692929
Free Energy -228.670108
Stoichiometry H5O3(1-)
Framework group C1[X(H5O3)]
Deg. of freedom 18
Full point group C1 NOp 1
Largest Abelian subgroup C1 NOp 1
Largest concise Abelian subgroup C1 NOp 1
Standard orientation:

Center Number	Atomic Number	Atomic Type	Coordinates (Angstroms) X	Y	Z
1	8	0	-0.000225	0.657416	0.000044
2	1	0	-0.000769	1.616660	-0.000144
3	8	0	-2.351148	-0.340430	0.086619
4	1	0	-2.355030	-0.801681	-0.754038
5	1	0	-1.410034	0.086466	0.103715
6	8	0	2.351414	-0.340291	-0.086624
7	1	0	1.410559	0.086532	-0.103892
8	1	0	2.354945	-0.801536	0.754044

Rotational constants (GHZ): 31.7538037 2.6124257 2.4454439

Benzoic Acid
Electronic Energy -573.247518
Free Energy -573.128679
Stoichiometry C7H10O4
Framework group C1[X(C7H10O4)]
Deg. of freedom 57
Full point group C1 NOp 1
Largest Abelian subgroup C1 NOp 1
Largest concise Abelian subgroup C1 NOp 1
Standard orientation:

Center Number	Atomic Number	Atomic Type	Coordinates (Angstroms) X	Y	Z
1	6	0	3.735768	0.106891	0.002680
2	6	0	3.097058	-1.126215	-0.025610

3	6	0	1.712108	-1.190953	-0.027032
4	6	0	0.963430	-0.016885	-0.000726
5	6	0	1.605855	1.217996	0.027132
6	6	0	2.989871	1.279025	0.029233
7	1	0	4.818361	0.154828	0.004047
8	1	0	3.679744	-2.039066	-0.046699
9	1	0	1.202615	-2.144733	-0.049253
10	1	0	1.004391	2.117522	0.047711
11	1	0	3.488943	2.240104	0.051620
12	6	0	-0.523535	-0.045995	-0.002089
13	8	0	-1.192883	0.974044	0.004883
14	8	0	-1.033631	-1.259598	-0.012255
15	1	0	-2.033438	-1.251274	-0.015869
16	8	0	-3.675401	-1.288071	-0.039580
17	1	0	-3.988039	-0.355181	-0.031632
18	1	0	-4.174075	-1.766065	0.627238
19	8	0	-3.893652	1.363338	0.043162
20	1	0	-4.202595	1.992395	-0.612469
21	1	0	-2.914707	1.390592	0.034105

Rotational constants (GHZ): 2.5684597 0.4655694 0.3946595

Benzoate

Electronic Energy -572.792802
Free Energy -572.688515
Stoichiometry C7H9O4(1-)
Framework group C1[X(C7H9O4)]
Deg. of freedom 54
Full point group C1 NOp 1
Largest Abelian subgroup C1 NOp 1
Largest concise Abelian subgroup C1 NOp 1
Standard orientation:

Center Number	Atomic Number	Atomic Type	Coordinates (Angstroms) X	Y	Z
1	6	0	-3.745801	0.071569	0.020554
2	6	0	-3.002058	1.259267	-0.015497
3	6	0	-1.603556	1.211305	-0.030543
4	6	0	-0.928747	-0.023306	-0.010084
5	6	0	-1.684567	-1.209685	0.025905
6	6	0	-3.083063	-1.163542	0.041340
7	1	0	-4.837986	0.108403	0.032383
8	1	0	-3.513623	2.224887	-0.032173
9	1	0	-1.023342	2.135857	-0.058552
10	1	0	-1.166750	-2.170910	0.042018
11	1	0	-3.658006	-2.092530	0.069494
12	6	0	0.588841	-0.075896	-0.025787
13	8	0	1.139612	-1.225402	-0.012468
14	8	0	1.212294	1.039515	-0.051589
15	8	0	3.884001	-1.432395	-0.024345
16	1	0	2.887463	-1.387129	-0.021573
17	1	0	4.115813	-0.473111	-0.011310
18	8	0	3.885273	1.398269	-0.024584
19	1	0	2.891347	1.244563	-0.009881
20	1	0	4.089354	1.751795	0.858159

Rotational constants (GHZ): 2.4971659 0.4591806 0.3883714

4-Cyanobenzoic Acid

Electronic Energy -665.416461
Free Energy -665.302267
Stoichiometry C8H9NO4
Framework group C1[X(C8H9NO4)]
Deg. of freedom 60
Full point group C1 NOp 1
Largest Abelian subgroup C1 NOp 1
Largest concise Abelian subgroup C1 NOp 1
Standard orientation:

Center Atomic Atomic Coordinates (Angstroms)
Number Number Type X Y Z

1	6	0	3.000493	0.037899	0.000660
2	6	0	2.342699	-1.191710	-0.025493
3	6	0	0.959623	-1.229468	-0.025870
4	6	0	0.230936	-0.043774	-0.001014
5	6	0	0.889881	1.181565	0.024506
6	6	0	2.271556	1.227502	0.025871
7	1	0	2.918243	-2.107919	-0.045351
8	1	0	0.436518	-2.175488	-0.046170
9	1	0	0.302906	2.090231	0.043876
10	1	0	2.792226	2.175946	0.046338
11	6	0	-1.261291	-0.049941	-0.001330
12	8	0	-1.906496	0.983294	0.002598
13	8	0	-1.783347	-1.254597	-0.007073
14	1	0	-2.787218	-1.239316	-0.011243
15	8	0	-4.403093	-1.251510	-0.043120
16	1	0	-4.718237	-0.319178	-0.031168
17	1	0	-4.920201	-1.744273	0.598546
18	8	0	-4.620755	1.395754	0.046924
19	1	0	-4.945074	2.035091	-0.591284
20	1	0	-3.644247	1.436877	0.032740
21	6	0	4.433440	0.079717	0.001548
22	7	0	5.581514	0.113394	0.002261

Rotational constants (GHZ): 2.5600692 0.2780027 0.2509715

4-Cyanobenzoate
Electronic Energy -664.9639160
Free Energy -664.864580
Stoichiometry C8H8NO4(1-)
Framework group C1[X(C8H8NO4)]
Deg. of freedom 57
Full point group C1 NOp 1
Largest Abelian subgroup C1 NOp 1
Largest concise Abelian subgroup C1 NOp 1
Standard orientation:

Center Number	Atomic Number	Atomic Type	Coordinates (Angstroms) X	Y	Z
1	6	0	3.000511	-0.003611	-0.000659
2	6	0	2.283426	-1.209869	-0.066660
3	6	0	0.894422	-1.187699	-0.066143
4	6	0	0.196052	0.022380	-0.001106
5	6	0	0.916764	1.219330	0.062927
6	6	0	2.305726	1.215868	0.064207
7	1	0	2.822907	-2.148863	-0.117649
8	1	0	0.315836	-2.101994	-0.116518
9	1	0	0.354204	2.143883	0.110850
10	1	0	2.862337	2.144821	0.114345
11	6	0	-1.342486	0.042071	-0.000024
12	8	0	-1.875392	1.170279	0.050653
13	8	0	-1.893079	-1.090661	-0.049038
14	8	0	-4.739548	1.481420	-0.127760
15	1	0	-3.764675	1.490368	-0.064241
16	1	0	-4.926961	0.530093	-0.159539
17	8	0	-4.568925	-1.478385	0.042441
18	1	0	-3.590819	-1.325879	-0.052199
19	1	0	-4.677427	-1.678920	0.976111
20	6	0	4.429985	-0.017268	0.000842
21	7	0	5.587680	-0.028850	0.002596

Rotational constants (GHZ): 2.4272317 0.2736266 0.2462903

2,6-Dimethylbenzoic Acid
Electronic Energy -651.780364
Free Energy -651.612299
Stoichiometry C9H14O4
Framework group C1[X(C9H14O4)]
Deg. of freedom 75
Full point group C1 NOp 1
Largest Abelian subgroup C1 NOp 1
Largest concise Abelian subgroup C1 NOp 1

Standard orientation:

Center Number	Atomic Number	Atomic Type	Coordinates (Angstroms) X	Y	Z
1	6	0	-3.601314	0.109154	0.047875
2	6	0	-2.952020	-1.097216	0.246430
3	6	0	-1.563559	-1.180047	0.208471
4	6	0	-0.832393	-0.002136	-0.012359
5	6	0	-1.476561	1.231992	-0.195461
6	6	0	-2.867034	1.261359	-0.176059
7	1	0	-4.683960	0.152000	0.070420
8	1	0	-3.527857	-1.996182	0.433285
9	1	0	-3.376242	2.204812	-0.334710
10	6	0	0.660407	-0.034545	-0.030237
11	8	0	1.340743	0.770892	0.582588
12	8	0	1.168255	-0.996973	-0.772151
13	1	0	2.167425	-1.007240	-0.744684
14	8	0	3.816042	-1.091702	-0.714449
15	1	0	4.136794	-0.342385	-0.163994
16	1	0	4.334446	-1.101575	-1.522481
17	8	0	4.044399	1.085682	0.801592
18	1	0	4.348478	1.203630	1.704210
19	1	0	3.065537	1.105340	0.819445
20	6	0	-0.912054	-2.521346	0.431335
21	1	0	-0.509944	-2.923478	-0.499160
22	1	0	-0.081343	-2.462128	1.136056
23	1	0	-1.641323	-3.228394	0.827483
24	6	0	-0.723139	2.516270	-0.427848
25	1	0	-0.222740	2.847318	0.482611
26	1	0	0.050628	2.405480	-1.189330
27	1	0	-1.409408	3.298698	-0.752673

Rotational constants (GHZ): 1.2645324 0.4352665 0.3465483

2,6-Dimethylbenzoate
Electronic Energy -651.327510
Free Energy -651.173977
Stoichiometry C9H13O4(1-)
Framework group C1[X(C9H13O4)]
Deg. of freedom 72
Full point group C1 NOp 1
Largest Abelian subgroup C1 NOp 1
Largest concise Abelian subgroup C1 NOp 1
Standard orientation:

Center Number	Atomic Number	Atomic Type	Coordinates (Angstroms) X	Y	Z
1	6	0	3.589988	-0.015340	0.027595
2	6	0	2.885682	-1.188575	-0.196226
3	6	0	1.492922	-1.195847	-0.209812
4	6	0	0.802254	0.006149	-0.013974
5	6	0	1.505403	1.197542	0.200838
6	6	0	2.897650	1.168935	0.229438
7	1	0	4.674714	-0.023741	0.043694
8	1	0	3.421265	-2.118775	-0.358835
9	1	0	3.442481	2.091134	0.406337
10	6	0	-0.722943	0.019924	-0.039801
11	8	0	-1.259157	0.720968	-0.917483
12	8	0	-1.287830	-0.683117	0.836528
13	8	0	-4.083411	0.777626	-1.243635
14	1	0	-3.107616	0.822589	-1.223633
15	1	0	-4.272274	0.195198	-0.492734
16	8	0	-3.921998	-0.821060	1.203958
17	1	0	-2.941330	-0.804685	1.024660
18	1	0	-4.032583	-0.213224	1.937879
19	6	0	0.775459	2.499936	0.401334
20	1	0	0.186432	2.747570	-0.482369
21	1	0	0.069487	2.431596	1.232851
22	1	0	1.478363	3.309322	0.611644
23	6	0	0.749309	-2.485618	-0.438899
24	1	0	0.132727	-2.729957	0.426776

25	1	0	0.067641	-2.400779	-1.288807
26	1	0	1.445514	-3.304213	-0.635363

Rotational constants (GHZ): 1.2552577 0.4228183 0.3564447

4-Bromobenzoic Acid
Electronic Energy -3146.389382
Free Energy -3146.283974
Stoichiometry C7H9BrO4
Framework group C1[X(C7H9BrO4)]
Deg. of freedom 57
Full point group C1 NOp 1
Largest Abelian subgroup C1 NOp 1
Largest concise Abelian subgroup C1 NOp 1
Standard orientation:

Center Number	Atomic Number	Atomic Type	Coordinates (Angstroms) X	Y	Z
1	6	0	-2.123583	-0.008463	-0.000380
2	6	0	-1.437005	1.213585	0.008335
3	6	0	-0.039215	1.233435	-0.000548
4	6	0	0.682321	0.024316	-0.016731
5	6	0	-0.012313	-1.199917	-0.024851
6	6	0	-1.409154	-1.214880	-0.017290
7	1	0	-1.992887	2.153777	0.021785
8	1	0	0.494504	2.184905	0.005980
9	1	0	0.551516	-2.134708	-0.037410
10	1	0	-1.943002	-2.167801	-0.024443
11	6	0	2.173421	0.005133	-0.024202
12	8	0	2.819160	-1.057321	-0.026977
13	8	0	2.734663	1.213506	-0.028234
14	1	0	3.768809	1.172350	-0.038972
15	8	0	5.329221	1.297732	-0.099798
16	1	0	5.600790	0.340359	0.007971
17	1	0	5.647463	1.744195	0.704982
18	8	0	5.564773	-1.379293	0.158208
19	1	0	5.876317	-1.801960	-0.661320
20	1	0	4.577005	-1.350163	0.066860
21	35	0	-4.033425	-0.030205	0.010942

Rotational constants (GHZ): 2.5286988 0.1821438 0.1700810

4-Bromobenzoate
Electronic Energy -3145.935481
Free Energy -3145.844089
Stoichiometry C7H8BrO4(1-)
Framework group C1[X(C7H8BrO4)]
Deg. of freedom 54
Full point group C1 NOp 1
Largest Abelian subgroup C1 NOp 1
Largest concise Abelian subgroup C1 NOp 1
Standard orientation:

Center Number	Atomic Number	Atomic Type	Coordinates (Angstroms) X	Y	Z
1	6	0	2.095862	-0.000814	-0.000142
2	6	0	1.369634	-1.199372	-0.033759
3	6	0	-0.029475	-1.172107	-0.043025
4	6	0	-0.722390	0.052372	-0.019138
5	6	0	0.015933	1.249790	0.014366
6	6	0	1.415010	1.224344	0.024022
7	1	0	1.895359	-2.157307	-0.053049
8	1	0	-0.596054	-2.105132	-0.069134
9	1	0	-0.515981	2.203239	0.033117
10	1	0	1.976254	2.161726	0.050300
11	6	0	-2.240642	0.082503	-0.028569
12	8	0	-2.808304	1.223725	-0.012377
13	8	0	-2.847621	-1.042001	-0.052394
14	8	0	-5.555481	1.390083	-0.012852
15	1	0	-4.558379	1.359570	-0.014205
16	1	0	-5.773016	0.427467	0.000630

17	8	0	-5.514865	-1.440289	-0.014579
18	1	0	-4.523270	-1.271897	-0.003885
19	1	0	-5.710051	-1.797209	0.868809
20	35	0	4.005477	-0.036940	0.012643

Rotational constants (GHZ): 2.4938611 0.1822606 0.1699478

2-Bromobenzoic Acid
Electronic Energy -3146.380455
Free Energy -3146.275805
Stoichiometry C7H9BrO4
Framework group C1[X(C7H9BrO4)]
Deg. of freedom 57
Full point group C1 NOp 1
Largest Abelian subgroup C1 NOp 1
Largest concise Abelian subgroup C1 NOp 1
Standard orientation:

Center Number	Atomic Number	Atomic Type	Coordinates (Angstroms) X	Y	Z
1	6	0	-3.444282	1.257609	0.023444
2	6	0	-2.606743	2.381605	0.018357
3	6	0	-1.217996	2.222056	0.003692
4	6	0	-0.657428	0.930336	-0.004453
5	6	0	-1.503234	-0.194791	0.001281
6	6	0	-2.890420	-0.030528	0.014577
7	1	0	-3.037496	3.385372	0.025536
8	1	0	-0.566735	3.097291	-0.000523
9	1	0	-3.541999	-0.907190	0.018181
10	6	0	0.818865	0.720129	-0.017728
11	8	0	1.323121	-0.416314	-0.013487
12	8	0	1.530285	1.846521	-0.034935
13	1	0	2.550563	1.673065	-0.049446
14	8	0	4.113846	1.596890	-0.117660
15	1	0	4.261055	0.613552	-0.002330
16	1	0	4.490799	2.005667	0.681769
17	8	0	4.005762	-1.085968	0.163592
18	1	0	4.256368	-1.551992	-0.653324
19	1	0	3.029412	-0.931246	0.075945
20	1	0	-4.506888	1.382717	0.034187
21	35	0	-0.734622	-1.943279	-0.009860

Rotational constants (GHZ): 0.8273102 0.4453857 0.2900520

2-Bromobenzoate
Electronic Energy -3145.929629
Free Energy -3145.838310
Stoichiometry C7H8BrO4(1-)
Framework group C1[X(C7H8BrO4)]
Deg. of freedom 54
Full point group C1 NOp 1
Largest Abelian subgroup C1 NOp 1
Largest concise Abelian subgroup C1 NOp 1
Standard orientation:

Center Number	Atomic Number	Atomic Type	Coordinates (Angstroms) X	Y	Z
1	6	0	3.449968	1.215491	0.032870
2	6	0	2.866897	-0.059213	0.017401
3	6	0	1.474063	-0.193615	0.000546
4	6	0	0.644558	0.943032	-0.001381
5	6	0	1.239774	2.217996	0.014058
6	6	0	2.632365	2.354207	0.031269
7	1	0	3.499594	-0.950332	0.018222
8	1	0	0.601451	3.103936	0.012747
9	1	0	3.081692	3.350366	0.043320
10	6	0	-0.866952	0.797735	-0.019056
11	8	0	-1.562396	1.866001	-0.026526
12	8	0	-1.340172	-0.389498	-0.025479
13	8	0	-4.310375	1.714462	-0.043959
14	1	0	-3.316412	1.799101	-0.039700

15	1	0	-4.415667	0.733569	-0.014054
16	8	0	-3.943982	-1.091944	0.006057
17	1	0	-2.978487	-0.810181	0.018711
18	1	0	-4.103053	-1.453471	0.894797
19	1	0	4.514884	1.318864	0.045823
20	35	0	0.677925	-1.929652	-0.020424

Rotational constants (GHZ): 0.8316482 0.4455310 0.2904179

2-Chlorobenzoic Acid

Electronic Energy -1032.670064
Free Energy -1032.563165
Stoichiometry C7H9ClO4
Framework group C1[X(C7H9ClO4)]
Deg. of freedom 57
Full point group C1 NOp 1
Largest Abelian subgroup C1 NOp 1
Largest concise Abelian subgroup C1 NOp 1
Standard orientation:

Center Number	Atomic Number	Atomic Type	Coordinates (Angstroms) X	Y	Z
1	6	0	-3.505240	-0.910756	0.141919
2	6	0	-3.020906	0.371280	-0.069580
3	6	0	-1.652989	0.593258	-0.100741
4	6	0	-0.752171	-0.458175	0.065903
5	6	0	-1.260936	-1.743885	0.249549
6	6	0	-2.625242	-1.972354	0.299750
7	1	0	-4.575039	-1.076257	0.173057
8	1	0	-3.702141	1.198911	-0.217039
9	1	0	-0.564479	-2.564256	0.367760
10	1	0	-2.999344	-2.975770	0.458166
11	6	0	0.733839	-0.319815	0.059097
12	8	0	1.438634	-1.123261	-0.534299
13	8	0	1.194721	0.680882	0.767809
14	1	0	2.205070	0.753644	0.742222
15	8	0	3.775044	1.014321	0.760119
16	1	0	4.132704	0.245012	0.263041
17	1	0	4.142743	0.952420	1.649999
18	8	0	4.220231	-1.238361	-0.692042
19	1	0	4.520846	-1.123774	-1.600938
20	1	0	3.244784	-1.271717	-0.736220
21	17	0	-1.113666	2.225637	-0.434596

Rotational constants (GHZ): 1.2223449 0.4288751 0.3372817

2-Chlorobenzoate

Electronic Energy -1032.219163
Free Energy -1032.127591
Stoichiometry C7H8ClO4(1-)
Framework group C1[X(C7H8ClO4)]
Deg. of freedom 54
Full point group C1 NOp 1
Largest Abelian subgroup C1 NOp 1
Largest concise Abelian subgroup C1 NOp 1
Standard orientation:

Center Number	Atomic Number	Atomic Type	Coordinates (Angstroms) X	Y	Z
1	6	0	3.552376	-0.689565	0.104386
2	6	0	2.767658	-1.835607	0.127458
3	6	0	1.385667	-1.731511	0.072792
4	6	0	0.767135	-0.485737	-0.003550
5	6	0	1.572521	0.644335	-0.027138
6	6	0	2.954876	0.560020	0.025611
7	1	0	4.632035	-0.762946	0.146923
8	1	0	3.231957	-2.812207	0.188239
9	1	0	0.771228	-2.624155	0.090550
10	1	0	3.554689	1.460709	0.005661
11	6	0	-0.742377	-0.398769	-0.054085
12	8	0	-1.278005	-0.466358	-1.185598

13	8	0	-1.337483	-0.283709	1.046379
14	8	0	-4.033525	-0.419624	-1.386739
15	1	0	-3.052729	-0.443496	-1.359800
16	1	0	-4.265439	-0.264842	-0.453863
17	8	0	-4.017922	-0.040897	1.415964
18	1	0	-3.042020	-0.100493	1.281564
19	1	0	-4.180939	0.854345	1.732903
20	17	0	0.831739	2.235222	-0.130887

Rotational constants (GHZ): 1.2422535 0.4132900 0.3588427

3-Chlorobenzoic Acid
Electronic Energy -1032.677698
Free Energy -1032.570548
Stoichiometry C7H9ClO4
Framework group C1[X(C7H9ClO4)]
Deg. of freedom 57
Full point group C1 NOp 1
Largest Abelian subgroup C1 NOp 1
Largest concise Abelian subgroup C1 NOp 1
Standard orientation:

Center Number	Atomic Number	Atomic Type	Coordinates (Angstroms) X	Y	Z
1	6	0	2.924424	1.163945	0.011212
2	6	0	2.522901	-0.162384	0.001551
3	6	0	1.187636	-0.519560	-0.006937
4	6	0	0.225296	0.487844	-0.005389
5	6	0	0.610212	1.825463	0.004260
6	6	0	1.955336	2.158003	0.012485
7	1	0	3.976439	1.417957	0.017702
8	1	0	0.896484	-1.560277	-0.014676
9	1	0	-0.145286	2.599725	0.005323
10	1	0	2.257867	3.197408	0.020058
11	6	0	-1.224833	0.155328	-0.013479
12	8	0	-2.090053	1.020264	-0.012029
13	8	0	-1.483656	-1.134564	-0.022958
14	1	0	-2.473351	-1.341893	-0.028541
15	8	0	-3.998053	-1.825490	-0.079580
16	1	0	-4.495013	-0.980167	-0.009858
17	1	0	-4.246391	-2.345569	0.694353
18	8	0	-4.853942	0.749406	0.107000
19	1	0	-5.272794	1.133628	-0.671683
20	1	0	-3.901059	0.952592	0.025418
21	17	0	3.741347	-1.423657	0.000013

Rotational constants (GHZ): 1.5691885 0.3154463 0.2629838

3-Chlorobenzoate
Electronic Energy -1032.224343
Free Energy -1032.130730
Stoichiometry C7H8ClO4(1-)
Framework group C1[X(C7H8ClO4)]
Deg. of freedom 54
Full point group C1 NOp 1
Largest Abelian subgroup C1 NOp 1
Largest concise Abelian subgroup C1 NOp 1
Standard orientation:

Center Number	Atomic Number	Atomic Type	Coordinates (Angstroms) X	Y	Z
1	6	0	-2.892033	1.167049	-0.015413
2	6	0	-1.911120	2.148498	-0.059670
3	6	0	-0.567967	1.800882	-0.069480
4	6	0	-0.187570	0.462311	-0.036661
5	6	0	-1.165316	-0.528211	0.006634
6	6	0	-2.498320	-0.160822	0.017127
7	1	0	-3.941688	1.430616	-0.006676
8	1	0	-2.202693	3.191227	-0.086085
9	1	0	0.192148	2.569597	-0.103306
10	1	0	-0.879044	-1.570526	0.032101

11	6	0	1.275240	0.073216	-0.045713
12	8	0	1.550483	-1.152968	-0.029787
13	8	0	2.120530	1.006583	-0.068082
14	8	0	4.172904	-1.954675	-0.139709
15	1	0	3.222506	-1.704363	-0.109672
16	1	0	4.618833	-1.096190	-0.031055
17	8	0	4.804972	0.788159	0.133952
18	1	0	3.820204	0.861739	0.083116
19	1	0	5.040870	1.087136	1.019001
20	17	0	-3.728219	-1.414328	0.073568

Rotational constants (GHZ): 1.5568445 0.3161640 0.2633674

4-Chlorobenzoic Acid
Electronic Energy -1032.678617
Free Energy -1032.571447
Stoichiometry C7H9ClO4
Framework group C1[X(C7H9ClO4)]
Deg. of freedom 57
Full point group C1 NOp 1
Largest Abelian subgroup C1 NOp 1
Largest concise Abelian subgroup C1 NOp 1
Standard orientation:

Center Number	Atomic Number	Atomic Type	Coordinates (Angstroms) X	Y	Z
1	6	0	2.841072	0.026948	0.004223
2	6	0	2.190205	-1.196515	0.014387
3	6	0	0.805076	-1.217076	0.005009
4	6	0	0.084063	-0.025153	-0.012962
5	6	0	0.761203	1.191933	-0.022515
6	6	0	2.145111	1.225851	-0.014601
7	1	0	2.754071	-2.119631	0.029051
8	1	0	0.287524	-2.166396	0.012481
9	1	0	0.201268	2.117585	-0.036494
10	1	0	2.673671	2.169725	-0.022735
11	6	0	-1.401080	-0.018826	-0.021376
12	8	0	-2.049705	1.019218	-0.022807
13	8	0	-1.947534	-1.216532	-0.027688
14	1	0	-2.956532	-1.186832	-0.038097
15	8	0	-4.558878	-1.302047	-0.101331
16	1	0	-4.846649	-0.367491	-0.000717
17	1	0	-4.920584	-1.777055	0.656469
18	8	0	-4.799305	1.394374	0.149628
19	1	0	-5.119386	1.876074	-0.621864
20	1	0	-3.826062	1.365894	0.060301
21	17	0	4.590715	0.059120	0.015658

Rotational constants (GHZ): 2.5425254 0.2624028 0.2381627

4-Chlorobenzoate
Electronic Energy -1032.224592
Free Energy -1032.131014
Stoichiometry C7H8ClO4(1-)
Framework group C1[X(C7H8ClO4)]
Deg. of freedom 54
Full point group C1 NOp 1
Largest Abelian subgroup C1 NOp 1
Largest concise Abelian subgroup C1 NOp 1
Standard orientation:

Center Number	Atomic Number	Atomic Type	Coordinates (Angstroms) X	Y	Z
1	6	0	2.810550	-0.020753	-0.001734
2	6	0	2.106204	-1.213631	-0.001527
3	6	0	0.720092	-1.170186	0.003746
4	6	0	0.042751	0.046488	0.008707
5	6	0	0.778393	1.228832	0.008541
6	6	0	2.164897	1.204884	0.003322
7	1	0	2.628731	-2.161286	-0.005603

8	1	0	0.158968	-2.095005	0.003580
9	1	0	0.262185	2.179492	0.012176
10	1	0	2.733140	2.125827	0.002986
11	6	0	-1.468317	0.084420	0.012463
12	8	0	-2.022280	1.213448	0.013300
13	8	0	-2.075010	-1.020038	0.013964
14	8	0	-4.758419	1.405075	-0.007489
15	1	0	-3.775605	1.371047	0.003192
16	1	0	-5.000805	0.462617	-0.024689
17	8	0	-4.740769	-1.423288	-0.106855
18	1	0	-3.765653	-1.271772	-0.038340
19	1	0	-4.985175	-1.905189	0.690827
20	17	0	4.564036	-0.062802	-0.008741

Rotational constants (GHZ): 2.5195961 0.2631042 0.2383770

2-Metoxybenzoic Acid
Electronic Energy -687.672853
Free Energy -687.525976
Stoichiometry C8H12O5
Framework group C1[X(C8H12O5)]
Deg. of freedom 69
Full point group C1 NOp 1
Largest Abelian subgroup C1 NOp 1
Largest concise Abelian subgroup C1 NOp 1
Standard orientation:

Center Number	Atomic Number	Atomic Type	Coordinates (Angstroms) X	Y	Z
1	6	0	3.298824	-1.276166	-0.135322
2	6	0	2.956147	0.060917	-0.004012
3	6	0	1.616210	0.444581	0.043790
4	6	0	0.613859	-0.541869	-0.037875
5	6	0	0.990364	-1.880467	-0.149719
6	6	0	2.319978	-2.257360	-0.208751
7	1	0	4.346977	-1.548001	-0.175820
8	1	0	3.737430	0.803531	0.065289
9	1	0	0.210419	-2.628881	-0.203913
10	1	0	2.588519	-3.300931	-0.310079
11	6	0	-0.845499	-0.267229	-0.002556
12	8	0	-1.640428	-1.103977	0.412086
13	8	0	-1.223877	0.900456	-0.475755
14	1	0	-2.221523	1.031913	-0.423380
15	8	0	-3.793570	1.429225	-0.381437
16	1	0	-4.218995	0.574247	-0.149635
17	1	0	-4.128731	1.666527	-1.254422
18	8	0	-4.411105	-1.121842	0.331869
19	1	0	-4.778835	-1.256111	1.212910
20	1	0	-3.440639	-1.193931	0.432418
21	8	0	1.235096	1.734189	0.200468
22	6	0	2.241185	2.738716	0.330157
23	1	0	1.702950	3.675581	0.452731
24	1	0	2.864479	2.556482	1.207824
25	1	0	2.862622	2.788429	-0.566031

Rotational constants (GHZ): 1.1793548 0.4201408 0.3171942

2-Metoxybenzoate
Electronic Energy -687.218472
Free Energy -687.085604
Stoichiometry C8H11O5(1-)
Framework group C1[X(C8H11O5)]
Deg. of freedom 66
Full point group C1 NOp 1
Largest Abelian subgroup C1 NOp 1
Largest concise Abelian subgroup C1 NOp 1
Standard orientation:

Center Number	Atomic Number	Atomic Type	Coordinates (Angstroms) X	Y	Z

1	6	0	3.322062	-1.166098	-0.136845
2	6	0	2.385440	-2.175424	-0.294385
3	6	0	1.034743	-1.854430	-0.258276
4	6	0	0.603682	-0.543193	-0.092748
5	6	0	1.562846	0.467969	0.073051
6	6	0	2.918406	0.149960	0.054482
7	1	0	4.381097	-1.395050	-0.154647
8	1	0	2.698726	-3.201789	-0.439303
9	1	0	0.290049	-2.633241	-0.370648
10	1	0	3.664539	0.919365	0.191537
11	6	0	-0.880177	-0.265146	-0.111587
12	8	0	-1.302792	0.666244	-0.841800
13	8	0	-1.605206	-1.023445	0.588038
14	8	0	-3.991186	1.224476	-0.919986
15	1	0	-3.023053	1.052245	-0.912227
16	1	0	-4.332348	0.536095	-0.322423
17	8	0	-4.293322	-0.981123	0.832006
18	1	0	-3.307360	-0.979853	0.754126
19	1	0	-4.479386	-0.770184	1.753556
20	8	0	1.103160	1.734124	0.282174
21	6	0	2.052407	2.776939	0.490021
22	1	0	2.699786	2.898739	-0.381162
23	1	0	1.467958	3.682458	0.636943
24	1	0	2.658314	2.585549	1.378520

Rotational constants (GHZ): 1.1675696 0.4147293 0.3280496

3-Metoxybenzoic Acid
Electronic Energy -687.677898
Free Energy -687.531421
Stoichiometry C8H12O5
Framework group C1[X(C8H12O5)]
Deg. of freedom 69
Full point group C1 NOp 1
Largest Abelian subgroup C1 NOp 1
Largest concise Abelian subgroup C1 NOp 1
Standard orientation:

Center Number	Atomic Number	Atomic Type	Coordinates (Angstroms) X	Y	Z
1	6	0	2.866096	1.041831	0.012478
2	6	0	2.452737	-0.285989	0.000460
3	6	0	1.095173	-0.594316	-0.010355
4	6	0	0.154104	0.425815	-0.009077
5	6	0	0.560668	1.760914	0.002843
6	6	0	1.911473	2.055007	0.013583
7	1	0	3.915829	1.299018	0.021118
8	1	0	0.792756	-1.632626	-0.019629
9	1	0	-0.178241	2.550579	0.003808
10	1	0	2.239578	3.087157	0.023156
11	6	0	-1.300643	0.119998	-0.019564
12	8	0	-2.152486	0.999534	-0.021852
13	8	0	-1.588182	-1.165219	-0.026965
14	1	0	-2.580684	-1.347771	-0.032691
15	8	0	-4.124803	-1.804859	-0.079357
16	1	0	-4.602747	-0.949403	-0.004263
17	1	0	-4.379113	-2.321232	0.695044
18	8	0	-4.913454	0.791679	0.118788
19	1	0	-5.327732	1.192456	-0.653922
20	1	0	-3.954961	0.965612	0.028964
21	8	0	3.304131	-1.352701	-0.001717
22	6	0	4.707043	-1.095743	0.008620
23	1	0	5.187282	-2.071493	0.004451
24	1	0	5.008633	-0.535938	-0.879552
25	1	0	4.997853	-0.548916	0.908410

Rotational constants (GHZ): 1.7374381 0.3044498 0.2598210

3-Metoxybenzoate
Electronic Energy -687.223297
Free Energy -687.090956

Stoichiometry   C8H11O5(1-)
Framework group C1[X(C8H11O5)]
Deg. of freedom   66
Full point group   C1   NOp   1
Largest Abelian subgroup   C1   NOp   1
Largest concise Abelian subgroup C1   NOp   1

Standard orientation:

Center Number	Atomic Number	Atomic Type	Coordinates (Angstroms) X	Y	Z
1	6	0	-2.849415	1.058542	-0.001259
2	6	0	-1.871216	2.047461	-0.008787
3	6	0	-0.529341	1.725125	-0.010821
4	6	0	-0.143711	0.385137	-0.005382
5	6	0	-1.111812	-0.602900	0.001966
6	6	0	-2.463340	-0.271383	0.003957
7	1	0	-3.884290	1.339755	0.000214
8	1	0	-2.174436	3.079230	-0.013025
9	1	0	0.211493	2.500614	-0.016718
10	1	0	-0.835068	-1.639534	0.006088
11	6	0	1.326292	0.006228	-0.007396
12	8	0	1.611093	-1.200870	-0.004525
13	8	0	2.154954	0.930398	-0.011600
14	8	0	4.360188	-2.036373	-0.004634
15	1	0	3.439416	-1.786786	-0.005070
16	1	0	4.827885	-1.213059	-0.003204
17	8	0	5.008899	0.965968	-0.062176
18	1	0	4.055344	0.943408	-0.023794
19	1	0	5.286858	1.441438	0.705277
20	8	0	-3.327147	-1.315641	0.011280
21	6	0	-4.718437	-1.069596	0.010050
22	1	0	-5.016153	-0.526732	-0.879829
23	1	0	-5.192822	-2.039863	0.014841
24	1	0	-5.016244	-0.518004	0.894499

Rotational constants (GHZ):   1.6790049   0.2976200   0.2533521

4-Metoxybenzoic Acid
Electronic Energy -687.680874
Free Energy -687.534558
Stoichiometry   C8H12O5
Framework group C1[X(C8H12O5)]
Deg. of freedom   69
Full point group   C1   NOp   1
Largest Abelian subgroup   C1   NOp   1
Largest concise Abelian subgroup C1   NOp   1

Standard orientation:

Center Number	Atomic Number	Atomic Type	Coordinates (Angstroms) X	Y	Z
1	6	0	-2.844676	0.234220	0.006450
2	6	0	-2.097906	1.415343	0.013691
3	6	0	-0.721183	1.360674	0.002968
4	6	0	-0.060793	0.127361	-0.014491
5	6	0	-0.815162	-1.042177	-0.021632
6	6	0	-2.200041	-1.001432	-0.011770
7	1	0	-2.618558	2.364721	0.027815
8	1	0	-0.151042	2.279805	0.008816
9	1	0	-0.312538	-2.000660	-0.035205
10	1	0	-2.761676	-1.924432	-0.018011
11	6	0	1.412320	0.035860	-0.023839
12	8	0	2.010830	-1.035062	-0.030481
13	8	0	2.031214	1.203008	-0.024914
14	1	0	3.033351	1.113010	-0.035161
15	8	0	4.656759	1.143694	-0.095385
16	1	0	4.887988	0.194376	0.009614
17	1	0	5.041434	1.599051	0.663007
18	8	0	4.721489	-1.564115	0.147815
19	1	0	5.015812	-2.061602	-0.623843
20	1	0	3.752139	-1.469822	0.050606
21	8	0	-4.192212	0.384373	0.018078

22	6	0	-5.008934	-0.787403	0.013937
23	1	0	-6.036111	-0.431107	0.025979
24	1	0	-4.836208	-1.378239	-0.887713
25	1	0	-4.820988	-1.394957	0.901310

Rotational constants (GHZ): 2.3158494 0.2713487 0.2435860

4-Metoxybenzoate
Electronic Energy -687.224797
Free Energy -687.092331
Stoichiometry C8H11O5(1-)
Framework group C1[X(C8H11O5)]
Deg. of freedom 66
Full point group C1 NOp 1
Largest Abelian subgroup C1 NOp 1
Largest concise Abelian subgroup C1 NOp 1
Standard orientation:

Center Number	Atomic Number	Atomic Type	Coordinates (Angstroms) X	Y	Z
1	6	0	-2.806789	0.279718	-0.000731
2	6	0	-2.011464	1.426517	-0.014764
3	6	0	-0.635644	1.311678	-0.016856
4	6	0	-0.018477	0.058012	-0.005136
5	6	0	-0.825838	-1.073895	0.008728
6	6	0	-2.211746	-0.978230	0.011056
7	1	0	-2.490831	2.397966	-0.023974
8	1	0	-0.026371	2.205981	-0.027909
9	1	0	-0.366125	-2.053646	0.017748
10	1	0	-2.808660	-1.879232	0.021809
11	6	0	1.481249	-0.065393	-0.007916
12	8	0	1.977103	-1.224023	0.003304
13	8	0	2.156599	1.001311	-0.021809
14	8	0	4.685677	-1.574652	-0.012338
15	1	0	3.705945	-1.476155	-0.006809
16	1	0	4.989388	-0.650386	-0.008683
17	8	0	4.838896	1.254309	-0.056321
18	1	0	3.854565	1.154893	-0.018953
19	1	0	5.084575	1.712583	0.754734
20	8	0	-4.153894	0.485991	0.000383
21	6	0	-5.011718	-0.653701	0.014407
22	1	0	-6.026267	-0.261928	0.013133
23	1	0	-4.856809	-1.270066	-0.873751
24	1	0	-4.851908	-1.251733	0.914157

Rotational constants (GHZ): 2.3408247 0.2714260 0.2437506

- **TPSSTPSS 6-311G+(d,p) SMD**

H_2O
Electronic Energy -229.426039
Free Energy -229.389029
Stoichiometry H6O3
Framework group C1[X(H6O3)]
Deg. of freedom 21
Full point group C1 NOp 1
Largest Abelian subgroup C1 NOp 1
Largest concise Abelian subgroup C1 NOp 1
Standard orientation:

Center Number	Atomic Number	Atomic Type	Coordinates (Angstroms) X	Y	Z
1	8	0	-0.000050	0.954311	0.010969
2	1	0	0.001861	1.358852	0.897066
3	1	0	-0.002358	1.710889	-0.612216
4	8	0	-2.422234	-0.491681	-0.047245
5	1	0	-2.202055	-1.404385	0.218886
6	1	0	-1.561031	-0.015559	-0.026959
7	8	0	2.421963	-0.491885	-0.049112

8	1	0	2.205188	-1.400281	0.234119
9	1	0	1.560954	-0.015480	-0.027796

Rotational constants (GHZ): 15.5792143 2.4807013 2.1647856

OH^-
Electronic Energy -228.946981
Free Energy -228.923196
Stoichiometry H5O3(1-)
Framework group C1[X(H5O3)]
Deg. of freedom 18
Full point group C1 NOp 1
Largest Abelian subgroup C1 NOp 1
Largest concise Abelian subgroup C1 NOp 1
Standard orientation:

Center Number	Atomic Number	Atomic Type	Coordinates (Angstroms) X	Y	Z
1	8	0	-0.000225	0.657416	0.000044
2	1	0	-0.000769	1.616660	-0.000144
3	8	0	-2.351148	-0.340430	0.086619
4	1	0	-2.355030	-0.801681	-0.754038
5	1	0	-1.410034	0.086466	0.103715
6	8	0	2.351414	-0.340291	-0.086624
7	1	0	1.410559	0.086532	-0.103892
8	1	0	2.354945	-0.801536	0.754044

Rotational constants (GHZ): 31.7538037 2.6124257 2.4454439

Benzoic Acid
Electronic Energy -573.980647
Free Energy -573.859964
Stoichiometry C7H10O4
Framework group C1[X(C7H10O4)]
Deg. of freedom 57
Full point group C1 NOp 1
Largest Abelian subgroup C1 NOp 1
Largest concise Abelian subgroup C1 NOp 1
Standard orientation:

Center Number	Atomic Number	Atomic Type	Coordinates (Angstroms) X	Y	Z
1	6	0	3.764356	0.066335	0.016739
2	6	0	3.093692	-1.163621	0.023223
3	6	0	1.697353	-1.201004	0.010071
4	6	0	0.962982	-0.000763	-0.008305
5	6	0	1.640807	1.231866	-0.014177
6	6	0	3.036250	1.263598	-0.002258
7	1	0	4.851395	0.092024	0.026522
8	1	0	3.658672	-2.092163	0.038191
9	1	0	1.177428	-2.153954	0.014773
10	1	0	1.068161	2.154935	-0.028442
11	1	0	3.556297	2.218107	-0.007633
12	6	0	-0.525271	0.000971	-0.020948
13	8	0	-1.187648	1.051589	-0.028139
14	8	0	-1.072301	-1.217202	-0.024227
15	1	0	-2.098623	-1.187988	-0.040648
16	8	0	-3.684497	-1.332535	-0.111566
17	1	0	-3.962753	-0.380818	-0.004380
18	1	0	-4.003942	-1.782530	0.688831
19	8	0	-3.938653	1.353689	0.150417
20	1	0	-4.250224	1.774616	-0.668196
21	1	0	-2.952634	1.329155	0.063028

Rotational constants (GHZ): 2.5259304 0.4592554 0.3895055

Benzoate
Electronic Energy -573.522762
Free Energy -573.150599

Stoichiometry C7H9O4(1-)
Framework group C1[X(C7H9O4)]
Deg. of freedom 54
Full point group C1 NOp 1
Largest Abelian subgroup C1 NOp 1
Largest concise Abelian subgroup C1 NOp 1
Standard orientation:

Center Number	Atomic Number	Atomic Type	Coordinates (Angstroms) X	Y	Z
1	6	0	-3.725998	0.076982	0.045715
2	6	0	-2.985856	1.253985	-0.014017
3	6	0	-1.597108	1.202602	-0.048732
4	6	0	-0.932323	-0.024546	-0.023445
5	6	0	-1.682540	-1.200123	0.036640
6	6	0	-3.071273	-1.150828	0.070801
7	1	0	-4.809426	0.116535	0.072598
8	1	0	-3.490987	2.213296	-0.033919
9	1	0	-1.023231	2.120279	-0.095486
10	1	0	-1.173932	-2.156501	0.056748
11	1	0	-3.643118	-2.070965	0.116971
12	6	0	0.581910	-0.081353	-0.058878
13	8	0	1.123596	-1.216195	-0.021312
14	8	0	1.202298	1.013478	-0.124157
15	8	0	3.871442	-1.436551	-0.034137
16	1	0	2.892649	-1.388205	-0.035301
17	1	0	4.123058	-0.500223	0.001103
18	8	0	3.883517	1.415642	0.045848
19	1	0	2.912683	1.257798	0.002071
20	1	0	4.044610	1.736679	0.936770

Rotational constants (GHZ): 2.5094836 0.4621435 0.3911077

4-Cyanobenzoic Acid

Electronic Energy -666.264661
Free Energy -666.148707
Stoichiometry C8H9NO4
Framework group C1[X(C8H9NO4)]
Deg. of freedom 60
Full point group C1 NOp 1
Largest Abelian subgroup C1 NOp 1
Largest concise Abelian subgroup C1 NOp 1
Standard orientation:

Center Number	Atomic Number	Atomic Type	Coordinates (Angstroms) X	Y	Z
1	6	0	3.000493	0.037899	0.000660
2	6	0	2.342699	-1.191710	-0.025493
3	6	0	0.959623	-1.229468	-0.025870
4	6	0	0.230936	-0.043774	-0.001014
5	6	0	0.889881	1.181565	0.024506
6	6	0	2.271556	1.227502	0.025871
7	1	0	2.918243	-2.107919	-0.045351
8	1	0	0.436518	-2.175488	-0.046170
9	1	0	0.302906	2.090231	0.043876
10	1	0	2.792226	2.175946	0.046338
11	6	0	-1.261291	-0.049941	-0.001330
12	8	0	-1.906496	0.983294	0.002598
13	8	0	-1.783347	-1.254597	-0.007073
14	1	0	-2.787218	-1.239316	-0.011243
15	8	0	-4.403093	-1.251510	-0.043120
16	1	0	-4.718237	-0.319178	-0.031168
17	1	0	-4.920201	-1.744273	0.598546
18	8	0	-4.620755	1.395754	0.046924
19	1	0	-4.945074	2.035091	-0.591284
20	1	0	-3.644247	1.436877	0.032740
21	6	0	4.433440	0.079717	0.001548
22	7	0	5.581514	0.113394	0.002261

Rotational constants (GHZ): 2.5600692 0.2780027 0.2509715

4-Cyanobenzoate
Electronic Energy -665.8090240
Free Energy -665.707885
Stoichiometry C8H8NO4(1-)
Framework group C1[X(C8H8NO4)]
Deg. of freedom 57
Full point group C1 NOp 1
Largest Abelian subgroup C1 NOp 1
Largest concise Abelian subgroup C1 NOp 1
Standard orientation:

Center Number	Atomic Number	Atomic Type	Coordinates (Angstroms) X	Y	Z
1	6	0	3.000511	-0.003611	-0.000659
2	6	0	2.283426	-1.209869	-0.066660
3	6	0	0.894422	-1.187699	-0.066143
4	6	0	0.196052	0.022380	-0.001106
5	6	0	0.916764	1.219330	0.062927
6	6	0	2.305726	1.215868	0.064207
7	1	0	2.822907	-2.148863	-0.117649
8	1	0	0.315836	-2.101994	-0.116518
9	1	0	0.354204	2.143883	0.110850
10	1	0	2.862337	2.144821	0.114345
11	6	0	-1.342486	0.042071	-0.000024
12	8	0	-1.875392	1.170279	0.050653
13	8	0	-1.893079	-1.090661	-0.049038
14	8	0	-4.739548	1.481420	-0.127760
15	1	0	-3.764675	1.490368	-0.064241
16	1	0	-4.926961	0.530093	-0.159539
17	8	0	-4.568925	-1.478385	0.042441
18	1	0	-3.590819	-1.325879	-0.052199
19	1	0	-4.677427	-1.678920	0.976111
20	6	0	4.429985	-0.017268	0.000842
21	7	0	5.587680	-0.028850	0.002596

Rotational constants (GHZ): 2.4272317 0.2736266 0.2462903

2,6-Dimethylbenzoic Acid
Electronic Energy -652.638050
Free Energy -652.467820
Stoichiometry C9H14O4
Framework group C1[X(C9H14O4)]
Deg. of freedom 75
Full point group C1 NOp 1
Largest Abelian subgroup C1 NOp 1
Largest concise Abelian subgroup C1 NOp 1
Standard orientation:

Center Number	Atomic Number	Atomic Type	Coordinates (Angstroms) X	Y	Z
1	6	0	-3.601314	0.109154	0.047875
2	6	0	-2.952020	-1.097216	0.246430
3	6	0	-1.563559	-1.180047	0.208471
4	6	0	-0.832393	-0.002136	-0.012359
5	6	0	-1.476561	1.231992	-0.195461
6	6	0	-2.867034	1.261359	-0.176059
7	1	0	-4.683960	0.152000	0.070420
8	1	0	-3.527857	-1.996182	0.433285
9	1	0	-3.376242	2.204812	-0.334710
10	6	0	0.660407	-0.034545	-0.030237
11	8	0	1.340743	0.770892	0.582588
12	8	0	1.168255	-0.996973	-0.772151
13	1	0	2.167425	-1.007240	-0.744684
14	8	0	3.816042	-1.091702	-0.714449
15	1	0	4.136794	-0.342385	-0.163994
16	1	0	4.334446	-1.101575	-1.522481
17	8	0	4.044399	1.085682	0.801592
18	1	0	4.348478	1.203630	1.704210
19	1	0	3.065537	1.105340	0.819445

20	6	0	-0.912054	-2.521346	0.431335
21	1	0	-0.509944	-2.923478	-0.499160
22	1	0	-0.081343	-2.462128	1.136056
23	1	0	-1.641323	-3.228394	0.827483
24	6	0	-0.723139	2.516270	-0.427848
25	1	0	-0.222740	2.847318	0.482611
26	1	0	0.050628	2.405480	-1.189330
27	1	0	-1.409408	3.298698	-0.752673

Rotational constants (GHZ): 1.2645324 0.4352665 0.3465483

2,6-Dimethylbenzoate
Electronic Energy -652.181741
Free Energy -652.025717
Stoichiometry C9H13O4(1-)
Framework group C1[X(C9H13O4)]
Deg. of freedom 72
Full point group C1 NOp 1
Largest Abelian subgroup C1 NOp 1
Largest concise Abelian subgroup C1 NOp 1
Standard orientation:

Center Number	Atomic Number	Atomic Type	Coordinates (Angstroms) X	Y	Z
1	6	0	3.589988	-0.015340	0.027595
2	6	0	2.885682	-1.188575	-0.196226
3	6	0	1.492922	-1.195847	-0.209812
4	6	0	0.802254	0.006149	-0.013974
5	6	0	1.505403	1.197542	0.200838
6	6	0	2.897650	1.168935	0.229438
7	1	0	4.674714	-0.023741	0.043694
8	1	0	3.421265	-2.118775	-0.358835
9	1	0	3.442481	2.091134	0.406337
10	6	0	-0.722943	0.019924	-0.039801
11	8	0	-1.259157	0.720968	-0.917483
12	8	0	-1.287830	-0.683117	0.836528
13	8	0	-4.083411	0.777626	-1.243635
14	1	0	-3.107616	0.822589	-1.223633
15	1	0	-4.272274	0.195198	-0.492734
16	8	0	-3.921998	-0.821060	1.203958
17	1	0	-2.941330	-0.804685	1.024660
18	1	0	-4.032583	-0.213224	1.937879
19	6	0	0.775459	2.499936	0.401334
20	1	0	0.186432	2.747570	-0.482369
21	1	0	0.069487	2.431596	1.232851
22	1	0	1.478363	3.309322	0.611644
23	6	0	0.749309	-2.485618	-0.438899
24	1	0	0.132727	-2.729957	0.426776
25	1	0	0.067641	-2.400779	-1.288807
26	1	0	1.445514	-3.304213	-0.635363

Rotational constants (GHZ): 1.2552577 0.4228183 0.3564447

4-Bromobenzoic Acid
Electronic Energy -3147.405937
Free Energy -3147.298918
Stoichiometry C7H9BrO4
Framework group C1[X(C7H9BrO4)]
Deg. of freedom 57
Full point group C1 NOp 1
Largest Abelian subgroup C1 NOp 1
Largest concise Abelian subgroup C1 NOp 1
Standard orientation:

Center Number	Atomic Number	Atomic Type	Coordinates (Angstroms) X	Y	Z
1	6	0	-2.122847	-0.008428	-0.000279
2	6	0	-1.437409	1.213339	0.008911
3	6	0	-0.040684	1.233865	0.000488
4	6	0	0.679202	0.024851	-0.015837

5	6	0	-0.013431	-1.199503	-0.024441
6	6	0	-1.409187	-1.214388	-0.017247
7	1	0	-1.991185	2.148630	0.022303
8	1	0	0.490689	2.180466	0.007288
9	1	0	0.548075	-2.129414	-0.037103
10	1	0	-1.940698	-2.162547	-0.024720
11	6	0	2.167360	0.005152	-0.023454
12	8	0	2.817026	-1.053384	-0.028785
13	8	0	2.729064	1.216633	-0.024449
14	1	0	3.755008	1.175035	-0.037414
15	8	0	5.342743	1.300447	-0.102923
16	1	0	5.609125	0.345406	0.004861
17	1	0	5.664888	1.746298	0.698710
18	8	0	5.563564	-1.388733	0.158953
19	1	0	5.872797	-1.813131	-0.658753
20	1	0	4.578214	-1.352268	0.068243
21	35	0	-4.032689	-0.030500	0.010439

Rotational constants (GHZ): 2.5227047 0.1820987 0.1700143

4-Bromobenzoate
Electronic Energy -3146,948970
Free Energy -3146.856901
Stoichiometry C7H8BrO4(1-)
Framework group C1[X(C7H8BrO4)]
Deg. of freedom 54
Full point group C1 NOp 1
Largest Abelian subgroup C1 NOp 1
Largest concise Abelian subgroup C1 NOp 1
Standard orientation:

Center Number	Atomic Number	Atomic Type	Coordinates (Angstroms) X	Y	Z
1	6	0	2.094046	-0.001027	0.000020
2	6	0	1.368073	-1.198872	-0.028884
3	6	0	-0.030046	-1.171391	-0.036857
4	6	0	-0.720871	0.053244	-0.016287
5	6	0	0.016489	1.250269	0.012551
6	6	0	1.414553	1.223975	0.020829
7	1	0	1.891076	-2.152387	-0.045419
8	1	0	-0.594148	-2.099778	-0.059396
9	1	0	-0.512405	2.199347	0.028584
10	1	0	1.973763	2.156580	0.043297
11	6	0	-2.236120	0.084094	-0.024755
12	8	0	-2.804637	1.225367	-0.012003
13	8	0	-2.845809	-1.039167	-0.044619
14	8	0	-5.550535	1.391370	-0.008424
15	1	0	-4.555136	1.361170	-0.010381
16	1	0	-5.773496	0.432062	-0.001269
17	8	0	-5.517252	-1.444133	-0.027492
18	1	0	-4.528608	-1.274972	-0.010034
19	1	0	-5.719914	-1.801499	0.852429
20	35	0	4.003657	-0.037994	0.010937

Rotational constants (GHZ): 2.4929112 0.1824236 0.1700787

2-Bromobenzoic Acid
Electronic Energy -3147.396523
Free Energy -3147.290392
Stoichiometry C7H9BrO4
Framework group C1[X(C7H9BrO4)]
Deg. of freedom 57
Full point group C1 NOp 1
Largest Abelian subgroup C1 NOp 1
Largest concise Abelian subgroup C1 NOp 1
Standard orientation:

Center Number	Atomic Number	Atomic Type	Coordinates (Angstroms) X	Y	Z
1	6	0	-3.443877	1.255668	0.022746

2	6	0	-2.608004	2.379910	0.018214
3	6	0	-1.220151	2.221791	0.004235
4	6	0	-0.660629	0.930635	-0.003886
5	6	0	-1.503965	-0.195249	0.001284
6	6	0	-2.890143	-0.031671	0.013980
7	1	0	-3.037746	3.378353	0.025247
8	1	0	-0.572229	3.092791	0.000402
9	1	0	-3.538405	-0.904202	0.017154
10	6	0	0.812775	0.720952	-0.017045
11	8	0	1.321923	-0.411903	-0.015268
12	8	0	1.524565	1.850710	-0.031193
13	1	0	2.536722	1.678300	-0.047725
14	8	0	4.127127	1.599322	-0.120473
15	1	0	4.269911	0.619030	-0.005052
16	1	0	4.507549	2.007178	0.675836
17	8	0	4.004056	-1.093710	0.164945
18	1	0	4.252496	-1.561073	-0.650060
19	1	0	3.030990	-0.932459	0.077697
20	1	0	-4.506566	1.380115	0.032976
21	35	0	-0.733718	-1.943017	-0.009935

Rotational constants (GHZ): 0.8268519 0.4448941 0.2897877

2-Bromobenzoate
Electronic Energy -3146.942216
Free Energy -3146.849658
Stoichiometry C7H8BrO4(1-)
Framework group C1[X(C7H8BrO4)]
Deg. of freedom 54
Full point group C1 NOp 1
Largest Abelian subgroup C1 NOp 1
Largest concise Abelian subgroup C1 NOp 1
Standard orientation:

Center Number	Atomic Number	Atomic Type	Coordinates (Angstroms) X	Y	Z
1	6	0	3.449246	1.211174	0.028555
2	6	0	2.865114	-0.062136	0.015276
3	6	0	1.473090	-0.194852	0.000738
4	6	0	0.646723	0.942912	-0.000951
5	6	0	1.242262	2.216709	0.012325
6	6	0	2.634094	2.350603	0.027149
7	1	0	3.493752	-0.949723	0.016007
8	1	0	0.608282	3.099207	0.011136
9	1	0	3.082911	3.341274	0.037426
10	6	0	-0.862043	0.800139	-0.016550
11	8	0	-1.557330	1.868969	-0.024211
12	8	0	-1.339234	-0.385649	-0.021452
13	8	0	-4.304179	1.719887	-0.035287
14	1	0	-3.311857	1.803696	-0.032402
15	1	0	-4.416087	0.741592	-0.014051
16	8	0	-3.946950	-1.093169	-0.009690
17	1	0	-2.984241	-0.811809	0.009447
18	1	0	-4.112854	-1.458043	0.874835
19	1	0	4.514328	1.313040	0.039689
20	35	0	0.674754	-1.929909	-0.017606

Rotational constants (GHZ): 0.8315399 0.4460127 0.2905864

2-Chlorobenzoic Acid
Electronic Energy -1033.602536
Free Energy -1033.494053
Stoichiometry C7H9ClO4
Framework group C1[X(C7H9ClO4)]
Deg. of freedom 57
Full point group C1 NOp 1
Largest Abelian subgroup C1 NOp 1
Largest concise Abelian subgroup C1 NOp 1
Standard orientation:

Center Atomic Atomic Coordinates (Angstroms)
Number Number Type X Y Z

1	6	0	-3.505240	-0.910756	0.141919
2	6	0	-3.020906	0.371280	-0.069580
3	6	0	-1.652989	0.593258	-0.100741
4	6	0	-0.752171	-0.458175	0.065903
5	6	0	-1.260936	-1.743885	0.249549
6	6	0	-2.625242	-1.972354	0.299750
7	1	0	-4.575039	-1.076257	0.173057
8	1	0	-3.702141	1.198911	-0.217039
9	1	0	-0.564479	-2.564256	0.367760
10	1	0	-2.999344	-2.975770	0.458166
11	6	0	0.733839	-0.319815	0.059097
12	8	0	1.438634	-1.123261	-0.534299
13	8	0	1.194721	0.680882	0.767809
14	1	0	2.205070	0.753644	0.742222
15	8	0	3.775044	1.014321	0.760119
16	1	0	4.132704	0.245012	0.263041
17	1	0	4.142743	0.952420	1.649999
18	8	0	4.220231	-1.238361	-0.692042
19	1	0	4.520846	-1.123774	-1.600938
20	1	0	3.244784	-1.271717	-0.736220
21	17	0	-1.113666	2.225637	-0.434596

Rotational constants (GHZ): 1.2223449 0.4288751 0.3372817

2-Chlorobenzoate
Electronic Energy -1033.148173
Free Energy -1033.055392
Stoichiometry C7H8ClO4(1-)
Framework group C1[X(C7H8ClO4)]
Deg. of freedom 54
Full point group C1 NOp 1
Largest Abelian subgroup C1 NOp 1
Largest concise Abelian subgroup C1 NOp 1
Standard orientation:

Center Number	Atomic Number	Atomic Type	Coordinates (Angstroms) X	Y	Z
1	6	0	3.552376	-0.689565	0.104386
2	6	0	2.767658	-1.835607	0.127458
3	6	0	1.385667	-1.731511	0.072792
4	6	0	0.767135	-0.485737	-0.003550
5	6	0	1.572521	0.644335	-0.027138
6	6	0	2.954876	0.560020	0.025611
7	1	0	4.632035	-0.762946	0.146923
8	1	0	3.231957	-2.812207	0.188239
9	1	0	0.771228	-2.624155	0.090550
10	1	0	3.554689	1.460709	0.005661
11	6	0	-0.742377	-0.398769	-0.054085
12	8	0	-1.278005	-0.466358	-1.185598
13	8	0	-1.337483	-0.283709	1.046379
14	8	0	-4.033525	-0.419624	-1.386739
15	1	0	-3.052729	-0.443496	-1.359800
16	1	0	-4.265439	-0.264842	-0.453863
17	8	0	-4.017922	-0.040897	1.415964
18	1	0	-3.042020	-0.100493	1.281564
19	1	0	-4.180939	0.854345	1.732903
20	17	0	0.831739	2.235222	-0.130887

Rotational constants (GHZ): 1.2422535 0.4132900 0.3588427

3-Chlorobenzoic Acid
Electronic Energy -1033.610581
Free Energy -1033.501840
Stoichiometry C7H9ClO4
Framework group C1[X(C7H9ClO4)]
Deg. of freedom 57
Full point group C1 NOp 1
Largest Abelian subgroup C1 NOp 1
Largest concise Abelian subgroup C1 NOp 1
Standard orientation:

Center Number	Atomic Number	Atomic Type	Coordinates (Angstroms) X	Y	Z
1	6	0	2.924424	1.163945	0.011212
2	6	0	2.522901	-0.162384	0.001551
3	6	0	1.187636	-0.519560	-0.006937
4	6	0	0.225296	0.487844	-0.005389
5	6	0	0.610212	1.825463	0.004260
6	6	0	1.955336	2.158003	0.012485
7	1	0	3.976439	1.417957	0.017702
8	1	0	0.896484	-1.560277	-0.014676
9	1	0	-0.145286	2.599725	0.005323
10	1	0	2.257867	3.197408	0.020058
11	6	0	-1.224833	0.155328	-0.013479
12	8	0	-2.090053	1.020264	-0.012029
13	8	0	-1.483656	-1.134564	-0.022958
14	1	0	-2.473351	-1.341893	-0.028541
15	8	0	-3.998053	-1.825490	-0.079580
16	1	0	-4.495013	-0.980167	-0.009858
17	1	0	-4.246391	-2.345569	0.694353
18	8	0	-4.853942	0.749406	0.107000
19	1	0	-5.272794	1.133628	-0.671683
20	1	0	-3.901059	0.952592	0.025418
21	17	0	3.741347	-1.423657	0.000013

Rotational constants (GHZ): 1.5691885 0.3154463 0.2629838

3-Chlorobenzoate
Electronic Energy -1033.154153
Free Energy -1033.059266
Stoichiometry C7H8ClO4(1-)
Framework group C1[X(C7H8ClO4)]
Deg. of freedom 54
Full point group C1 NOp 1
Largest Abelian subgroup C1 NOp 1
Largest concise Abelian subgroup C1 NOp 1
Standard orientation:

Center Number	Atomic Number	Atomic Type	Coordinates (Angstroms) X	Y	Z
1	6	0	-2.892033	1.167049	-0.015413
2	6	0	-1.911120	2.148498	-0.059670
3	6	0	-0.567967	1.800882	-0.069480
4	6	0	-0.187570	0.462311	-0.036661
5	6	0	-1.165316	-0.528211	0.006634
6	6	0	-2.498320	-0.160822	0.017127
7	1	0	-3.941688	1.430616	-0.006676
8	1	0	-2.202693	3.191227	-0.086085
9	1	0	0.192148	2.569597	-0.103306
10	1	0	-0.879044	-1.570526	0.032101
11	6	0	1.275240	0.073216	-0.045713
12	8	0	1.550483	-1.152968	-0.029787
13	8	0	2.120530	1.006583	-0.068082
14	8	0	4.172904	-1.954675	-0.139709
15	1	0	3.222506	-1.704363	-0.109672
16	1	0	4.618833	-1.096190	-0.031055
17	8	0	4.804972	0.788159	0.133952
18	1	0	3.820204	0.861739	0.083116
19	1	0	5.040870	1.087136	1.019001
20	17	0	-3.728219	-1.414328	0.073568

Rotational constants (GHZ): 1.5568445 0.3161640 0.2633674

4-Chlorobenzoic Acid
Electronic Energy -1033.611486
Free Energy -1033.502691
Stoichiometry C7H9ClO4
Framework group C1[X(C7H9ClO4)]
Deg. of freedom 57
Full point group C1 NOp 1
Largest Abelian subgroup C1 NOp 1
Largest concise Abelian subgroup C1 NOp 1

Standard orientation:

Center Number	Atomic Number	Atomic Type	Coordinates (Angstroms) X	Y	Z
1	6	0	2.841072	0.026948	0.004223
2	6	0	2.190205	-1.196515	0.014387
3	6	0	0.805076	-1.217076	0.005009
4	6	0	0.084063	-0.025153	-0.012962
5	6	0	0.761203	1.191933	-0.022515
6	6	0	2.145111	1.225851	-0.014601
7	1	0	2.754071	-2.119631	0.029051
8	1	0	0.287524	-2.166396	0.012481
9	1	0	0.201268	2.117585	-0.036494
10	1	0	2.673671	2.169725	-0.022735
11	6	0	-1.401080	-0.018826	-0.021376
12	8	0	-2.049705	1.019218	-0.022807
13	8	0	-1.947534	-1.216532	-0.027688
14	1	0	-2.956532	-1.186832	-0.038097
15	8	0	-4.558878	-1.302047	-0.101331
16	1	0	-4.846649	-0.367491	-0.000717
17	1	0	-4.920584	-1.777055	0.656469
18	8	0	-4.799305	1.394374	0.149628
19	1	0	-5.119386	1.876074	-0.621864
20	1	0	-3.826062	1.365894	0.060301
21	17	0	4.590715	0.059120	0.015658

Rotational constants (GHZ): 2.5425254 0.2624028 0.2381627

4-Chlorobenzoate
Electronic Energy -1033.154395
Free Energy -1033.059533

2-Metoxybenzoic Acid
Electronic Energy -688,550467
Free Energy -688,401909
Stoichiometry C8H12O5
Framework group C1[X(C8H12O5)]
Deg. of freedom 69
Full point group C1 NOp 1
Largest Abelian subgroup C1 NOp 1
Largest concise Abelian subgroup C1 NOp 1
Standard orientation:

Center Number	Atomic Number	Atomic Type	Coordinates (Angstroms) X	Y	Z
1	6	0	3.298824	-1.276166	-0.135322
2	6	0	2.956147	0.060917	-0.004012
3	6	0	1.616210	0.444581	0.043790
4	6	0	0.613859	-0.541869	-0.037875
5	6	0	0.990364	-1.880467	-0.149719
6	6	0	2.319978	-2.257360	-0.208751
7	1	0	4.346977	-1.548001	-0.175820
8	1	0	3.737430	0.803531	0.065289
9	1	0	0.210419	-2.628881	-0.203913
10	1	0	2.588519	-3.300931	-0.310079
11	6	0	-0.845499	-0.267229	-0.002556
12	8	0	-1.640428	-1.103977	0.412086
13	8	0	-1.223877	0.900456	-0.475755
14	1	0	-2.221523	1.031913	-0.423380
15	8	0	-3.793570	1.429225	-0.381437
16	1	0	-4.218995	0.574247	-0.149635
17	1	0	-4.128731	1.666527	-1.254422
18	8	0	-4.411105	-1.121842	0.331869
19	1	0	-4.778835	-1.256111	1.212910
20	1	0	-3.440639	-1.193931	0.432418
21	8	0	1.235096	1.734189	0.200468
22	6	0	2.241185	2.738716	0.330157
23	1	0	1.702950	3.675581	0.452731

24	1	0	2.864479	2.556482	1.207824
25	1	0	2.862622	2.788429	-0.566031

Rotational constants (GHZ): 1.1793548 0.4201408 0.3171942

2-Metoxybenzoate
Electronic Energy -688.092693
Free Energy -687.957389
Stoichiometry C8H11O5(1-)
Framework group C1[X(C8H11O5)]
Deg. of freedom 66
Full point group C1 NOp 1
Largest Abelian subgroup C1 NOp 1
Largest concise Abelian subgroup C1 NOp 1
Standard orientation:

Center Number	Atomic Number	Atomic Type	Coordinates (Angstroms) X	Y	Z
1	6	0	3.322062	-1.166098	-0.136845
2	6	0	2.385440	-2.175424	-0.294385
3	6	0	1.034743	-1.854430	-0.258276
4	6	0	0.603682	-0.543193	-0.092748
5	6	0	1.562846	0.467969	0.073051
6	6	0	2.918406	0.149960	0.054482
7	1	0	4.381097	-1.395050	-0.154647
8	1	0	2.698726	-3.201789	-0.439303
9	1	0	0.290049	-2.633241	-0.370648
10	1	0	3.664539	0.919365	0.191537
11	6	0	-0.880177	-0.265146	-0.111587
12	8	0	-1.302792	0.666244	-0.841800
13	8	0	-1.605206	-1.023445	0.588038
14	8	0	-3.991186	1.224476	-0.919986
15	1	0	-3.023053	1.052245	-0.912227
16	1	0	-4.332348	0.536095	-0.322423
17	8	0	-4.293322	-0.981123	0.832006
18	1	0	-3.307360	-0.979853	0.754126
19	1	0	-4.479386	-0.770184	1.753556
20	8	0	1.103160	1.734124	0.282174
21	6	0	2.052407	2.776939	0.490021
22	1	0	2.699786	2.898739	-0.381162
23	1	0	1.467958	3.682458	0.636943
24	1	0	2.658314	2.585549	1.378520

Rotational constants (GHZ): 1.1675696 0.4147293 0.3280496

3-Metoxybenzoic Acid
Electronic Energy -688.555447
Free Energy -688.406740
Stoichiometry C8H12O5
Framework group C1[X(C8H12O5)]
Deg. of freedom 69
Full point group C1 NOp 1
Largest Abelian subgroup C1 NOp 1
Largest concise Abelian subgroup C1 NOp 1
Standard orientation:

Center Number	Atomic Number	Atomic Type	Coordinates (Angstroms) X	Y	Z
1	6	0	2.866096	1.041831	0.012478
2	6	0	2.452737	-0.285989	0.000460
3	6	0	1.095173	-0.594316	-0.010355
4	6	0	0.154104	0.425815	-0.009077
5	6	0	0.560668	1.760914	0.002843
6	6	0	1.911473	2.055007	0.013583
7	1	0	3.915829	1.299018	0.021118
8	1	0	0.792756	-1.632626	-0.019629
9	1	0	-0.178241	2.550579	0.003808
10	1	0	2.239578	3.087157	0.023156
11	6	0	-1.300643	0.119998	-0.019564
12	8	0	-2.152486	0.999534	-0.021852

13	8	0	-1.588182	-1.165219	-0.026965
14	1	0	-2.580684	-1.347771	-0.032691
15	8	0	-4.124803	-1.804859	-0.079357
16	1	0	-4.602747	-0.949403	-0.004263
17	1	0	-4.379113	-2.321232	0.695044
18	8	0	-4.913454	0.791679	0.118788
19	1	0	-5.327732	1.192456	-0.653922
20	1	0	-3.954961	0.965612	0.028964
21	8	0	3.304131	-1.352701	-0.001717
22	6	0	4.707043	-1.095743	0.008620
23	1	0	5.187282	-2.071493	0.004451
24	1	0	5.008633	-0.535938	-0.879552
25	1	0	4.997853	-0.548916	0.908410

Rotational constants (GHZ): 1.7374381 0.3044498 0.2598210

3-Metoxybenzoate
Electronic Energy -688.097698
Free Energy -687.963431
Stoichiometry C8H11O5(1-)
Framework group C1[X(C8H11O5)]
Deg. of freedom 66
Full point group C1 NOp 1
Largest Abelian subgroup C1 NOp 1
Largest concise Abelian subgroup C1 NOp 1
Standard orientation:

Center Number	Atomic Number	Atomic Type	Coordinates (Angstroms) X	Y	Z
1	6	0	-2.833574	1.042319	-0.015976
2	6	0	-1.866749	2.043160	-0.063083
3	6	0	-0.517837	1.733534	-0.073039
4	6	0	-0.115280	0.397311	-0.036606
5	6	0	-1.072407	-0.605631	0.009269
6	6	0	-2.427993	-0.286904	0.019828
7	1	0	-3.880972	1.309078	-0.008466
8	1	0	-2.183790	3.078930	-0.091827
9	1	0	0.225776	2.517751	-0.109425
10	1	0	-0.775314	-1.645832	0.037338
11	6	0	1.354057	0.037150	-0.045506
12	8	0	1.658866	-1.182965	-0.025666
13	8	0	2.183132	0.986541	-0.071596
14	8	0	4.296716	-1.926003	-0.123474
15	1	0	3.340590	-1.696350	-0.097356
16	1	0	4.722473	-1.056039	-0.024445
17	8	0	4.867528	0.830131	0.127625
18	1	0	3.880626	0.881570	0.078127
19	1	0	5.098226	1.134246	1.012298
20	8	0	-3.289981	-1.348480	0.067082
21	6	0	-4.689331	-1.078319	0.070850
22	1	0	-4.989495	-0.551438	-0.837983
23	1	0	-5.179934	-2.048426	0.107132
24	1	0	-4.973581	-0.492994	0.948411

Rotational constants (GHZ): 1.7267938 0.3052022 0.2603155

4-Metoxybenzoic Acid
Electronic Energy -688.558547
Free Energy -688.410153
Stoichiometry C8H12O5
Framework group C1[X(C8H12O5)]
Deg. of freedom 69
Full point group C1 NOp 1
Largest Abelian subgroup C1 NOp 1
Largest concise Abelian subgroup C1 NOp 1
Standard orientation:

Center Number	Atomic Number	Atomic Type	Coordinates (Angstroms) X	Y	Z
1	6	0	-2.844676	0.234220	0.006450

2	6	0	-2.097906	1.415343	0.013691
3	6	0	-0.721183	1.360674	0.002968
4	6	0	-0.060793	0.127361	-0.014491
5	6	0	-0.815162	-1.042177	-0.021632
6	6	0	-2.200041	-1.001432	-0.011770
7	1	0	-2.618558	2.364721	0.027815
8	1	0	-0.151042	2.279805	0.008816
9	1	0	-0.312538	-2.000660	-0.035205
10	1	0	-2.761676	-1.924432	-0.018011
11	6	0	1.412320	0.035860	-0.023839
12	8	0	2.010830	-1.035062	-0.030481
13	8	0	2.031214	1.203008	-0.024914
14	1	0	3.033351	1.113010	-0.035161
15	8	0	4.656759	1.143694	-0.095385
16	1	0	4.887988	0.194376	0.009614
17	1	0	5.041434	1.599051	0.663007
18	8	0	4.721489	-1.564115	0.147815
19	1	0	5.015812	-2.061602	-0.623843
20	1	0	3.752139	-1.469822	0.050606
21	8	0	-4.192212	0.384373	0.018078
22	6	0	-5.008934	-0.787403	0.013937
23	1	0	-6.036111	-0.431107	0.025979
24	1	0	-4.836208	-1.378239	-0.887713
25	1	0	-4.820988	-1.394957	0.901310

Rotational constants (GHZ): 2.3158494 0.2713487 0.2435860

4-Metoxybenzoate

Electronic Energy -688.099331
Free Energy -687.964837
Stoichiometry C8H11O5(1-)
Framework group C1[X(C8H11O5)]
Deg. of freedom 66
Full point group C1 NOp 1
Largest Abelian subgroup C1 NOp 1
Largest concise Abelian subgroup C1 NOp 1
Standard orientation:

Center Number	Atomic Number	Atomic Type	Coordinates (Angstroms) X	Y	Z
1	6	0	-2.806789	0.279718	-0.000731
2	6	0	-2.011464	1.426517	-0.014764
3	6	0	-0.635644	1.311678	-0.016856
4	6	0	-0.018477	0.058012	-0.005136
5	6	0	-0.825838	-1.073895	0.008728
6	6	0	-2.211746	-0.978230	0.011056
7	1	0	-2.490831	2.397966	-0.023974
8	1	0	-0.026371	2.205981	-0.027909
9	1	0	-0.366125	-2.053646	0.017748
10	1	0	-2.808660	-1.879232	0.021809
11	6	0	1.481249	-0.065393	-0.007916
12	8	0	1.977103	-1.224023	0.003304
13	8	0	2.156599	1.001311	-0.021809
14	8	0	4.685677	-1.574652	-0.012338
15	1	0	3.705945	-1.476155	-0.006809
16	1	0	4.989388	-0.650386	-0.008683
17	8	0	4.838896	1.254309	-0.056321
18	1	0	3.854565	1.154893	-0.018953
19	1	0	5.084575	1.712583	0.754734
20	8	0	-4.153894	0.485991	0.000383
21	6	0	-5.011718	-0.653701	0.014407
22	1	0	-6.026267	-0.261928	0.013133
23	1	0	-4.856809	-1.270066	-0.873751
24	1	0	-4.851908	-1.251733	0.914157

Rotational constants (GHZ): 2.3408247 0.2714260 0.2437506

- **WB97XD 6-311G+(d,p) SMD**

H_2O

Electronic Energy -229.348561
Free Energy -229.310122
Stoichiometry H6O3
Framework group C1[X(H6O3)]
Deg. of freedom 21
Full point group C1 NOp 1
Largest Abelian subgroup C1 NOp 1
Largest concise Abelian subgroup C1 NOp 1
Standard orientation:

Center Number	Atomic Number	Atomic Type	Coordinates (Angstroms) X	Y	Z
1	8	0	-0.000006	0.958979	0.001077
2	1	0	-0.000010	1.540137	0.768360
3	1	0	0.000056	1.549462	-0.759107
4	8	0	-2.419522	-0.492235	-0.002508
5	1	0	-2.183728	-1.423016	0.011992
6	1	0	-1.568850	-0.019822	-0.000879
7	8	0	2.419518	-0.492230	-0.002600
8	1	0	2.183789	-1.423009	0.013019
9	1	0	1.568818	-0.019868	-0.001142

Rotational constants (GHZ): 15.5364509 2.4893015 2.1671808

OH^-
Electronic Energy -228.864253
Free Energy -228.837962
Stoichiometry H5O3(1-)
Framework group C1[X(H5O3)]
Deg. of freedom 18
Full point group C1 NOp 1
Largest Abelian subgroup C1 NOp 1
Largest concise Abelian subgroup C1 NOp 1
Standard orientation:

Center Number	Atomic Number	Atomic Type	Coordinates (Angstroms) X	Y	Z
1	8	0	-0.000225	0.657416	0.000044
2	1	0	-0.000769	1.616660	-0.000144
3	8	0	-2.351148	-0.340430	0.086619
4	1	0	-2.355030	-0.801681	-0.754038
5	1	0	-1.410034	0.086466	0.103715
6	8	0	2.351414	-0.340291	-0.086624
7	1	0	1.410559	0.086532	-0.103892
8	1	0	2.354945	-0.801536	0.754044

Rotational constants (GHZ): 31.7538037 2.6124257 2.4454439

Benzoic Acid
Electronic Energy -573.720175
Free Energy -573.859964
Stoichiometry C7H10O4
Framework group C1[X(C7H10O4)]
Deg. of freedom 57
Full point group C1 NOp 1
Largest Abelian subgroup C1 NOp 1
Largest concise Abelian subgroup C1 NOp 1
Standard orientation:

Center Number	Atomic Number	Atomic Type	Coordinates (Angstroms) X	Y	Z
1	6	0	3.764356	0.066335	0.016739
2	6	0	3.093692	-1.163621	0.023223
3	6	0	1.697353	-1.201004	0.010071
4	6	0	0.962982	-0.000763	-0.008305
5	6	0	1.640807	1.231866	-0.014177
6	6	0	3.036250	1.263598	-0.002258
7	1	0	4.851395	0.092024	0.026522
8	1	0	3.658672	-2.092163	0.038191
9	1	0	1.177428	-2.153954	0.014773

10	1	0	1.068161	2.154935	-0.028442
11	1	0	3.556297	2.218107	-0.007633
12	6	0	-0.525271	0.000971	-0.020948
13	8	0	-1.187648	1.051589	-0.028139
14	8	0	-1.072301	-1.217202	-0.024227
15	1	0	-2.098623	-1.187988	-0.040648
16	8	0	-3.684497	-1.332535	-0.111566
17	1	0	-3.962753	-0.380818	-0.004380
18	1	0	-4.003942	-1.782530	0.688831
19	8	0	-3.938653	1.353689	0.150417
20	1	0	-4.250224	1.774616	-0.668196
21	1	0	-2.952634	1.329155	0.063028

Rotational constants (GHZ): 2.5259304 0.4592554 0.3895055

Benzoate
Electronic Energy -573.261859
Free Energy -573.416992
Stoichiometry C7H9O4(1-)
Framework group C1[X(C7H9O4)]
Deg. of freedom 54
Full point group C1 NOp 1
Largest Abelian subgroup C1 NOp 1
Largest concise Abelian subgroup C1 NOp 1
Standard orientation:

Center Number	Atomic Number	Atomic Type	Coordinates (Angstroms) X	Y	Z
1	6	0	-3.720624	0.022668	0.007030
2	6	0	-3.005310	1.212486	-0.064270
3	6	0	-1.616519	1.190681	-0.069484
4	6	0	-0.927316	-0.016101	-0.004110
5	6	0	-1.649300	-1.203296	0.066191
6	6	0	-3.037897	-1.186655	0.072259
7	1	0	-4.805497	0.037672	0.011129
8	1	0	-3.533051	2.159168	-0.116394
9	1	0	-1.032571	2.100991	-0.124914
10	1	0	-1.089515	-2.129199	0.114788
11	1	0	-3.591161	-2.118436	0.127366
12	6	0	0.601606	-0.042828	-0.009109
13	8	0	1.137093	-1.165428	0.044420
14	8	0	1.164096	1.080327	-0.064987
15	8	0	3.948397	-1.470471	-0.129504
16	1	0	2.972676	-1.474610	-0.067280
17	1	0	4.141583	-0.521615	-0.159821
18	8	0	3.795460	1.450279	0.059800
19	1	0	2.815594	1.299870	-0.051435
20	1	0	3.893736	1.626762	0.997688

Rotational constants (GHZ): 2.4728657 0.4643442 0.3919859

4-Cyanobenzoic Acid
Electronic Energy -665.951361
Free Energy -665.830391
Stoichiometry C8H9NO4
Framework group C1[X(C8H9NO4)]
Deg. of freedom 60
Full point group C1 NOp 1
Largest Abelian subgroup C1 NOp 1
Largest concise Abelian subgroup C1 NOp 1
Standard orientation:

Center Number	Atomic Number	Atomic Type	Coordinates (Angstroms) X	Y	Z
1	6	0	3.000493	0.037899	0.000660
2	6	0	2.342699	-1.191710	-0.025493
3	6	0	0.959623	-1.229468	-0.025870
4	6	0	0.230936	-0.043774	-0.001014
5	6	0	0.889881	1.181565	0.024506
6	6	0	2.271556	1.227502	0.025871
7	1	0	2.918243	-2.107919	-0.045351

8	1	0	0.436518	-2.175488	-0.046170
9	1	0	0.302906	2.090231	0.043876
10	1	0	2.792226	2.175946	0.046338
11	6	0	-1.261291	-0.049941	-0.001330
12	8	0	-1.906496	0.983294	0.002598
13	8	0	-1.783347	-1.254597	-0.007073
14	1	0	-2.787218	-1.239316	-0.011243
15	8	0	-4.403093	-1.251510	-0.043120
16	1	0	-4.718237	-0.319178	-0.031168
17	1	0	-4.920201	-1.744273	0.598546
18	8	0	-4.620755	1.395754	0.046924
19	1	0	-4.945074	2.035091	-0.591284
20	1	0	-3.644247	1.436877	0.032740
21	6	0	4.433440	0.079717	0.001548
22	7	0	5.581514	0.113394	0.002261

Rotational constants (GHZ): 2.5600692 0.2780027 0.2509715

4-Cyanobenzoate
Electronic Energy -665.4952570
Free Energy -665.388246
Stoichiometry C8H8NO4(1-)
Framework group C1[X(C8H8NO4)]
Deg. of freedom 57
Full point group C1 NOp 1
Largest Abelian subgroup C1 NOp 1
Largest concise Abelian subgroup C1 NOp 1
Standard orientation:

Center Number	Atomic Number	Atomic Type	Coordinates (Angstroms) X	Y	Z
1	6	0	3.000511	-0.003611	-0.000659
2	6	0	2.283426	-1.209869	-0.066660
3	6	0	0.894422	-1.187699	-0.066143
4	6	0	0.196052	0.022380	-0.001106
5	6	0	0.916764	1.219330	0.062927
6	6	0	2.305726	1.215868	0.064207
7	1	0	2.822907	-2.148863	-0.117649
8	1	0	0.315836	-2.101994	-0.116518
9	1	0	0.354204	2.143883	0.110850
10	1	0	2.862337	2.144821	0.114345
11	6	0	-1.342486	0.042071	-0.000024
12	8	0	-1.875392	1.170279	0.050653
13	8	0	-1.893079	-1.090661	-0.049038
14	8	0	-4.739548	1.481420	-0.127760
15	1	0	-3.764675	1.490368	-0.064241
16	1	0	-4.926961	0.530093	-0.159539
17	8	0	-4.568925	-1.478385	0.042441
18	1	0	-3.590819	-1.325879	-0.052199
19	1	0	-4.677427	-1.678920	0.976111
20	6	0	4.429985	-0.017268	0.000842
21	7	0	5.587680	-0.028850	0.002596

Rotational constants (GHZ): 2.4272317 0.2736266 0.2462903

2,6-Dimethylbenzoic Acid
Electronic Energy -652.345619
Free Energy -652.168947
Stoichiometry C9H14O4
Framework group C1[X(C9H14O4)]
Deg. of freedom 75
Full point group C1 NOp 1
Largest Abelian subgroup C1 NOp 1
Largest concise Abelian subgroup C1 NOp 1
Standard orientation:

Center Number	Atomic Number	Atomic Type	Coordinates (Angstroms) X	Y	Z
1	6	0	-3.601314	0.109154	0.047875
2	6	0	-2.952020	-1.097216	0.246430
3	6	0	-1.563559	-1.180047	0.208471

4	6	0	-0.832393	-0.002136	-0.012359
5	6	0	-1.476561	1.231992	-0.195461
6	6	0	-2.867034	1.261359	-0.176059
7	1	0	-4.683960	0.152000	0.070420
8	1	0	-3.527857	-1.996182	0.433285
9	1	0	-3.376242	2.204812	-0.334710
10	6	0	0.660407	-0.034545	-0.030237
11	8	0	1.340743	0.770892	0.582588
12	8	0	1.168255	-0.996973	-0.772151
13	1	0	2.167425	-1.007240	-0.744684
14	8	0	3.816042	-1.091702	-0.714449
15	1	0	4.136794	-0.342385	-0.163994
16	1	0	4.334446	-1.101575	-1.522481
17	8	0	4.044399	1.085682	0.801592
18	1	0	4.348478	1.203630	1.704210
19	1	0	3.065537	1.105340	0.819445
20	6	0	-0.912054	-2.521346	0.431335
21	1	0	-0.509944	-2.923478	-0.499160
22	1	0	-0.081343	-2.462128	1.136056
23	1	0	-1.641323	-3.228394	0.827483
24	6	0	-0.723139	2.516270	-0.427848
25	1	0	-0.222740	2.847318	0.482611
26	1	0	0.050628	2.405480	-1.189330
27	1	0	-1.409408	3.298698	-0.752673

Rotational constants (GHZ): 1.2645324 0.4352665 0.3465483

2,6-Dimethylbenzoate
Electronic Energy -651.888733
Free Energy -651.726532
Stoichiometry C9H13O4(1-)
Framework group C1[X(C9H13O4)]
Deg. of freedom 72
Full point group C1 NOp 1
Largest Abelian subgroup C1 NOp 1
Largest concise Abelian subgroup C1 NOp 1
Standard orientation:

Center Number	Atomic Number	Atomic Type	Coordinates (Angstroms) X	Y	Z
1	6	0	3.589988	-0.015340	0.027595
2	6	0	2.885682	-1.188575	-0.196226
3	6	0	1.492922	-1.195847	-0.209812
4	6	0	0.802254	0.006149	-0.013974
5	6	0	1.505403	1.197542	0.200838
6	6	0	2.897650	1.168935	0.229438
7	1	0	4.674714	-0.023741	0.043694
8	1	0	3.421265	-2.118775	-0.358835
9	1	0	3.442481	2.091134	0.406337
10	6	0	-0.722943	0.019924	-0.039801
11	8	0	-1.259157	0.720968	-0.917483
12	8	0	-1.287830	-0.683117	0.836528
13	8	0	-4.083411	0.777626	-1.243635
14	1	0	-3.107616	0.822589	-1.223633
15	1	0	-4.272274	0.195198	-0.492734
16	8	0	-3.921998	-0.821060	1.203958
17	1	0	-2.941330	-0.804685	1.024660
18	1	0	-4.032583	-0.213224	1.937879
19	6	0	0.775459	2.499936	0.401334
20	1	0	0.186432	2.747570	-0.482369
21	1	0	0.069487	2.431596	1.232851
22	1	0	1.478363	3.309322	0.611644
23	6	0	0.749309	-2.485618	-0.438899
24	1	0	0.132727	-2.729957	0.426776
25	1	0	0.067641	-2.400779	-1.288807
26	1	0	1.445514	-3.304213	-0.635363

Rotational constants (GHZ): 1.2552577 0.4228183 0.3564447

4-Bromobenzoic Acid
Electronic Energy -3147.292901
Free Energy -3147.181126

Stoichiometry C7H9BrO4
Framework group C1[X(C7H9BrO4)]
Deg. of freedom 57
Full point group C1 NOp 1
Largest Abelian subgroup C1 NOp 1
Largest concise Abelian subgroup C1 NOp 1
Standard orientation:

Center Number	Atomic Number	Atomic Type	Coordinates (Angstroms) X	Y	Z
1	6	0	-2.113240	-0.009019	-0.000045
2	6	0	-1.437785	1.207654	0.009334
3	6	0	-0.049326	1.232997	0.002131
4	6	0	0.668548	0.035394	-0.013163
5	6	0	-0.012125	-1.183336	-0.021886
6	6	0	-1.399807	-1.204247	-0.015893
7	1	0	-1.993286	2.138150	0.021947
8	1	0	0.474655	2.180346	0.009130
9	1	0	0.548606	-2.110210	-0.033829
10	1	0	-1.925445	-2.151972	-0.023597
11	6	0	2.155872	0.024824	-0.020011
12	8	0	2.805421	-1.012698	-0.018853
13	8	0	2.704465	1.222831	-0.027925
14	1	0	3.704440	1.181221	-0.038415
15	8	0	5.346425	1.298819	-0.097346
16	1	0	5.620585	0.364891	0.000724
17	1	0	5.685148	1.749932	0.681534
18	8	0	5.563878	-1.429716	0.146495
19	1	0	5.860889	-1.868018	-0.655772
20	1	0	4.594455	-1.376474	0.065173
21	35	0	-4.022982	-0.039067	0.008895

Rotational constants (GHZ): 2.5251072 0.1827810 0.1706062

4-Bromobenzoate
Electronic Energy -3146.835568
Free Energy -3146.737202
Stoichiometry C7H8BrO4(1-)
Framework group C1[X(C7H8BrO4)]
Deg. of freedom 54
Full point group C1 NOp 1
Largest Abelian subgroup C1 NOp 1
Largest concise Abelian subgroup C1 NOp 1
Standard orientation:

Center Number	Atomic Number	Atomic Type	Coordinates (Angstroms) X	Y	Z
1	6	0	2.082039	-0.000758	-0.000952
2	6	0	1.360320	-1.189405	-0.055312
3	6	0	-0.029337	-1.160250	-0.077014
4	6	0	-0.713428	0.056054	-0.043937
5	6	0	0.018390	1.243436	0.010680
6	6	0	1.407993	1.216369	0.031831
7	1	0	1.880545	-2.140472	-0.081167
8	1	0	-0.588852	-2.086954	-0.119608
9	1	0	-0.505262	2.191514	0.036770
10	1	0	1.965432	2.145501	0.073864
11	6	0	-2.228642	0.088621	-0.065180
12	8	0	-2.788062	1.214591	-0.021092
13	8	0	-2.831995	-1.015938	-0.126096
14	8	0	-5.539085	1.390885	-0.008017
15	1	0	-4.559697	1.358237	-0.018368
16	1	0	-5.775330	0.450604	0.028350
17	8	0	-5.504723	-1.461225	0.068371
18	1	0	-4.536993	-1.287789	0.015752
19	1	0	-5.652324	-1.785827	0.960338
20	35	0	3.991411	-0.039878	0.028516

Rotational constants (GHZ): 2.5039130 0.1834997 0.1711475

2-Bromobenzoic Acid

Electronic Energy -3147.284010
Free Energy -3147.172868
Stoichiometry C7H9BrO4
Framework group C1[X(C7H9BrO4)]
Deg. of freedom 57
Full point group C1 NOp 1
Largest Abelian subgroup C1 NOp 1
Largest concise Abelian subgroup C1 NOp 1
Standard orientation:

Center Number	Atomic Number	Atomic Type	Coordinates (Angstroms) X	Y	Z
1	6	0	-3.442538	1.220781	0.020116
2	6	0	-2.625541	2.347337	0.016930
3	6	0	-1.244167	2.205304	0.004888
4	6	0	-0.675704	0.929912	-0.002645
5	6	0	-1.498126	-0.198052	0.001231
6	6	0	-2.878201	-0.051722	0.012024
7	1	0	-3.064968	3.338005	0.023506
8	1	0	-0.609964	3.082716	0.002110
9	1	0	-3.514098	-0.929315	0.014105
10	6	0	0.799494	0.740331	-0.014358
11	8	0	1.319465	-0.367792	-0.006569
12	8	0	1.488231	1.863488	-0.034254
13	1	0	2.475877	1.701729	-0.047723
14	8	0	4.119810	1.620345	-0.113118
15	1	0	4.280010	0.661022	-0.008049
16	1	0	4.513726	2.033632	0.660777
17	8	0	4.008380	-1.112483	0.153118
18	1	0	4.247013	-1.589766	-0.646390
19	1	0	3.052067	-0.943589	0.074577
20	1	0	-4.506612	1.332893	0.028831
21	35	0	-0.713470	-1.939404	-0.009265

Rotational constants (GHZ): 0.8304806 0.4465084 0.2908788

2-Bromobenzoate
Electronic Energy -3146.829503
Free Energy -3146.731376
Stoichiometry C7H8BrO4(1-)
Framework group C1[X(C7H8BrO4)]
Deg. of freedom 54
Full point group C1 NOp 1
Largest Abelian subgroup C1 NOp 1
Largest concise Abelian subgroup C1 NOp 1
Standard orientation:

Center Number	Atomic Number	Atomic Type	Coordinates (Angstroms) X	Y	Z
1	6	0	3.432006	1.206830	0.066092
2	6	0	2.851219	-0.057579	0.040183
3	6	0	1.467740	-0.188060	0.002404
4	6	0	0.649165	0.942547	-0.009179
5	6	0	1.240125	2.206862	0.017136
6	6	0	2.623321	2.339394	0.054301
7	1	0	3.476697	-0.943315	0.049087
8	1	0	0.611579	3.089245	0.008521
9	1	0	3.070648	3.326967	0.074022
10	6	0	-0.859436	0.801121	-0.047977
11	8	0	-1.544033	1.856590	-0.044431
12	8	0	-1.332123	-0.366452	-0.081714
13	8	0	-4.297136	1.717666	-0.066836
14	1	0	-3.320355	1.796863	-0.065472
15	1	0	-4.425248	0.758008	-0.004345
16	8	0	-3.939257	-1.108394	0.097025
17	1	0	-2.996923	-0.827114	0.049718
18	1	0	-4.061712	-1.421639	0.996825
19	1	0	4.496842	1.307781	0.094959
20	35	0	0.674972	-1.925393	-0.033526

Rotational constants (GHZ): 0.8352829 0.4484053 0.2923100

2-Chlorobenzoic Acid
Electronic Energy -1033.318874
Free Energy -1033.205927
Stoichiometry C7H9ClO4
Framework group C1[X(C7H9ClO4)]
Deg. of freedom 57
Full point group C1 NOp 1
Largest Abelian subgroup C1 NOp 1
Largest concise Abelian subgroup C1 NOp 1
Standard orientation:

Center Number	Atomic Number	Atomic Type	Coordinates (Angstroms) X	Y	Z
1	6	0	-3.505240	-0.910756	0.141919
2	6	0	-3.020906	0.371280	-0.069580
3	6	0	-1.652989	0.593258	-0.100741
4	6	0	-0.752171	-0.458175	0.065903
5	6	0	-1.260936	-1.743885	0.249549
6	6	0	-2.625242	-1.972354	0.299750
7	1	0	-4.575039	-1.076257	0.173057
8	1	0	-3.702141	1.198911	-0.217039
9	1	0	-0.564479	-2.564256	0.367760
10	1	0	-2.999344	-2.975770	0.458166
11	6	0	0.733839	-0.319815	0.059097
12	8	0	1.438634	-1.123261	-0.534299
13	8	0	1.194721	0.680882	0.767809
14	1	0	2.205070	0.753644	0.742222
15	8	0	3.775044	1.014321	0.760119
16	1	0	4.132704	0.245012	0.263041
17	1	0	4.142743	0.952420	1.649999
18	8	0	4.220231	-1.238361	-0.692042
19	1	0	4.520846	-1.123774	-1.600938
20	1	0	3.244784	-1.271717	-0.736220
21	17	0	-1.113666	2.225637	-0.434596

Rotational constants (GHZ): 1.2223449 0.4288751 0.3372817

2-Chlorobenzoate
Electronic Energy -1032.864371
Free Energy -1032.766248
Stoichiometry C7H8ClO4(1-)
Framework group C1[X(C7H8ClO4)]
Deg. of freedom 54
Full point group C1 NOp 1
Largest Abelian subgroup C1 NOp 1
Largest concise Abelian subgroup C1 NOp 1
Standard orientation:

Center Number	Atomic Number	Atomic Type	Coordinates (Angstroms) X	Y	Z
1	6	0	3.556395	-0.688816	0.103156
2	6	0	2.771098	-1.837026	0.125621
3	6	0	1.386958	-1.733664	0.071524
4	6	0	0.766846	-0.486211	-0.003566
5	6	0	1.573090	0.646359	-0.026786
6	6	0	2.958071	0.562835	0.025417
7	1	0	4.636826	-0.761749	0.145253
8	1	0	3.236268	-2.814077	0.185504
9	1	0	0.772235	-2.627201	0.088875
10	1	0	3.559015	1.463888	0.005818
11	6	0	-0.744689	-0.400086	-0.053186
12	8	0	-1.279194	-0.464643	-1.185262
13	8	0	-1.337807	-0.290028	1.048619
14	8	0	-4.036734	-0.419575	-1.387405
15	1	0	-3.058611	-0.443045	-1.355959
16	1	0	-4.267217	-0.264103	-0.456733
17	8	0	-4.018929	-0.039590	1.416180
18	1	0	-3.046359	-0.108690	1.282819
19	1	0	-4.166770	0.856933	1.727558

20	17	0	0.829959	2.236964	-0.130192

Rotational constants (GHZ): 1.2403387 0.4127757 0.3583249

3-Chlorobenzoic Acid
Electronic Energy -1033.326634
Free Energy -1033.213156
Stoichiometry C7H9ClO4
Framework group C1[X(C7H9ClO4)]
Deg. of freedom 57
Full point group C1 NOp 1
Largest Abelian subgroup C1 NOp 1
Largest concise Abelian subgroup C1 NOp 1
Standard orientation:

Center Number	Atomic Number	Atomic Type	Coordinates (Angstroms) X	Y	Z
1	6	0	2.924424	1.163945	0.011212
2	6	0	2.522901	-0.162384	0.001551
3	6	0	1.187636	-0.519560	-0.006937
4	6	0	0.225296	0.487844	-0.005389
5	6	0	0.610212	1.825463	0.004260
6	6	0	1.955336	2.158003	0.012485
7	1	0	3.976439	1.417957	0.017702
8	1	0	0.896484	-1.560277	-0.014676
9	1	0	-0.145286	2.599725	0.005323
10	1	0	2.257867	3.197408	0.020058
11	6	0	-1.224833	0.155328	-0.013479
12	8	0	-2.090053	1.020264	-0.012029
13	8	0	-1.483656	-1.134564	-0.022958
14	1	0	-2.473351	-1.341893	-0.028541
15	8	0	-3.998053	-1.825490	-0.079580
16	1	0	-4.495013	-0.980167	-0.009858
17	1	0	-4.246391	-2.345569	0.694353
18	8	0	-4.853942	0.749406	0.107000
19	1	0	-5.272794	1.133628	-0.671683
20	1	0	-3.901059	0.952592	0.025418
21	17	0	3.741347	-1.423657	0.000013

Rotational constants (GHZ): 1.5691885 0.3154463 0.2629838

3-Chlorobenzoate
Electronic Energy -1032.869786
Free Energy -1032.769547
Stoichiometry C7H8ClO4(1-)
Framework group C1[X(C7H8ClO4)]
Deg. of freedom 54
Full point group C1 NOp 1
Largest Abelian subgroup C1 NOp 1
Largest concise Abelian subgroup C1 NOp 1
Standard orientation:

Center Number	Atomic Number	Atomic Type	Coordinates (Angstroms) X	Y	Z
1	6	0	-2.892033	1.167049	-0.015413
2	6	0	-1.911120	2.148498	-0.059670
3	6	0	-0.567967	1.800882	-0.069480
4	6	0	-0.187570	0.462311	-0.036661
5	6	0	-1.165316	-0.528211	0.006634
6	6	0	-2.498320	-0.160822	0.017127
7	1	0	-3.941688	1.430616	-0.006676
8	1	0	-2.202693	3.191227	-0.086085
9	1	0	0.192148	2.569597	-0.103306
10	1	0	-0.879044	-1.570526	0.032101
11	6	0	1.275240	0.073216	-0.045713
12	8	0	1.550483	-1.152968	-0.029787
13	8	0	2.120530	1.006583	-0.068082
14	8	0	4.172904	-1.954675	-0.139709
15	1	0	3.222506	-1.704363	-0.109672
16	1	0	4.618833	-1.096190	-0.031055
17	8	0	4.804972	0.788159	0.133952

18	1	0	3.820204	0.861739	0.083116
19	1	0	5.040870	1.087136	1.019001
20	17	0	-3.728219	-1.414328	0.073568

Rotational constants (GHZ): 1.5568445 0.3161640 0.2633674

4-Chlorobenzoic Acid
Electronic Energy -1033.327399
Free Energy -1033.213953
Stoichiometry C7H9ClO4
Framework group C1[X(C7H9ClO4)]
Deg. of freedom 57
Full point group C1 NOp 1
Largest Abelian subgroup C1 NOp 1
Largest concise Abelian subgroup C1 NOp 1
Standard orientation:

Center Number	Atomic Number	Atomic Type	Coordinates (Angstroms) X	Y	Z
1	6	0	2.841072	0.026948	0.004223
2	6	0	2.190205	-1.196515	0.014387
3	6	0	0.805076	-1.217076	0.005009
4	6	0	0.084063	-0.025153	-0.012962
5	6	0	0.761203	1.191933	-0.022515
6	6	0	2.145111	1.225851	-0.014601
7	1	0	2.754071	-2.119631	0.029051
8	1	0	0.287524	-2.166396	0.012481
9	1	0	0.201268	2.117585	-0.036494
10	1	0	2.673671	2.169725	-0.022735
11	6	0	-1.401080	-0.018826	-0.021376
12	8	0	-2.049705	1.019218	-0.022807
13	8	0	-1.947534	-1.216532	-0.027688
14	1	0	-2.956532	-1.186832	-0.038097
15	8	0	-4.558878	-1.302047	-0.101331
16	1	0	-4.846649	-0.367491	-0.000717
17	1	0	-4.920584	-1.777055	0.656469
18	8	0	-4.799305	1.394374	0.149628
19	1	0	-5.119386	1.876074	-0.621864
20	1	0	-3.826062	1.365894	0.060301
21	17	0	4.590715	0.059120	0.015658

Rotational constants (GHZ): 2.5425254 0.2624028 0.2381627

4-Chlorobenzoate
Electronic Energy -1032.869969
Free Energy -1032.770110
Stoichiometry C7H8ClO4(1-)
Framework group C1[X(C7H8ClO4)]
Deg. of freedom 54
Full point group C1 NOp 1
Largest Abelian subgroup C1 NOp 1
Largest concise Abelian subgroup C1 NOp 1
Standard orientation:

Center Number	Atomic Number	Atomic Type	Coordinates (Angstroms) X	Y	Z
1	6	0	2.810550	-0.020753	-0.001734
2	6	0	2.106204	-1.213631	-0.001527
3	6	0	0.720092	-1.170186	0.003746
4	6	0	0.042751	0.046488	0.008707
5	6	0	0.778393	1.228832	0.008541
6	6	0	2.164897	1.204884	0.003322
7	1	0	2.628731	-2.161286	-0.005603
8	1	0	0.158968	-2.095005	0.003580
9	1	0	0.262185	2.179492	0.012176
10	1	0	2.733140	2.125827	0.002986
11	6	0	-1.468317	0.084420	0.012463
12	8	0	-2.022280	1.213448	0.013300
13	8	0	-2.075010	-1.020038	0.013964
14	8	0	-4.758419	1.405075	-0.007489

15	1	0	-3.775605	1.371047	0.003192
16	1	0	-5.000805	0.462617	-0.024689
17	8	0	-4.740769	-1.423288	-0.106855
18	1	0	-3.765653	-1.271772	-0.038340
19	1	0	-4.985175	-1.905189	0.690827
20	17	0	4.564036	-0.062802	-0.008741

Rotational constants (GHZ): 2.5195961 0.2631042 0.2383770

2-Metoxybenzoic Acid
Electronic Energy -688.237381
Free Energy -688.083381
Stoichiometry C8H12O5
Framework group C1[X(C8H12O5)]
Deg. of freedom 69
Full point group C1 NOp 1
Largest Abelian subgroup C1 NOp 1
Largest concise Abelian subgroup C1 NOp 1
Standard orientation:

Center Number	Atomic Number	Atomic Type	Coordinates (Angstroms) X	Y	Z
1	6	0	3.298824	-1.276166	-0.135322
2	6	0	2.956147	0.060917	-0.004012
3	6	0	1.616210	0.444581	0.043790
4	6	0	0.613859	-0.541869	-0.037875
5	6	0	0.990364	-1.880467	-0.149719
6	6	0	2.319978	-2.257360	-0.208751
7	1	0	4.346977	-1.548001	-0.175820
8	1	0	3.737430	0.803531	0.065289
9	1	0	0.210419	-2.628881	-0.203913
10	1	0	2.588519	-3.300931	-0.310079
11	6	0	-0.845499	-0.267229	-0.002556
12	8	0	-1.640428	-1.103977	0.412086
13	8	0	-1.223877	0.900456	-0.475755
14	1	0	-2.221523	1.031913	-0.423380
15	8	0	-3.793570	1.429225	-0.381437
16	1	0	-4.218995	0.574247	-0.149635
17	1	0	-4.128731	1.666527	-1.254422
18	8	0	-4.411105	-1.121842	0.331869
19	1	0	-4.778835	-1.256111	1.212910
20	1	0	-3.440639	-1.193931	0.432418
21	8	0	1.235096	1.734189	0.200468
22	6	0	2.241185	2.738716	0.330157
23	1	0	1.702950	3.675581	0.452731
24	1	0	2.864479	2.556482	1.207824
25	1	0	2.862622	2.788429	-0.566031

Rotational constants (GHZ): 1.1793548 0.4201408 0.3171942

2-Metoxybenzoate
Electronic Energy -687.779584
Free Energy -687.639250
Stoichiometry C8H11O5(1-)
Framework group C1[X(C8H11O5)]
Deg. of freedom 66
Full point group C1 NOp 1
Largest Abelian subgroup C1 NOp 1
Largest concise Abelian subgroup C1 NOp 1
Standard orientation:

Center Number	Atomic Number	Atomic Type	Coordinates (Angstroms) X	Y	Z
1	6	0	3.322062	-1.166098	-0.136845
2	6	0	2.385440	-2.175424	-0.294385
3	6	0	1.034743	-1.854430	-0.258276
4	6	0	0.603682	-0.543193	-0.092748
5	6	0	1.562846	0.467969	0.073051
6	6	0	2.918406	0.149960	0.054482
7	1	0	4.381097	-1.395050	-0.154647

8	1	0	2.698726	-3.201789	-0.439303
9	1	0	0.290049	-2.633241	-0.370648
10	1	0	3.664539	0.919365	0.191537
11	6	0	-0.880177	-0.265146	-0.111587
12	8	0	-1.302792	0.666244	-0.841800
13	8	0	-1.605206	-1.023445	0.588038
14	8	0	-3.991186	1.224476	-0.919986
15	1	0	-3.023053	1.052245	-0.912227
16	1	0	-4.332348	0.536095	-0.322423
17	8	0	-4.293322	-0.981123	0.832006
18	1	0	-3.307360	-0.979853	0.754126
19	1	0	-4.479386	-0.770184	1.753556
20	8	0	1.103160	1.734124	0.282174
21	6	0	2.052407	2.776939	0.490021
22	1	0	2.699786	2.898739	-0.381162
23	1	0	1.467958	3.682458	0.636943
24	1	0	2.658314	2.585549	1.378520

Rotational constants (GHZ): 1.1675696 0.4147293 0.3280496

3-Metoxybenzoic Acid

Electronic Energy -688.242314
Free Energy -688.088018
Stoichiometry C8H12O5
Framework group C1[X(C8H12O5)]
Deg. of freedom 69
Full point group C1 NOp 1
Largest Abelian subgroup C1 NOp 1
Largest concise Abelian subgroup C1 NOp 1
Standard orientation:

Center Number	Atomic Number	Atomic Type	Coordinates (Angstroms) X	Y	Z
1	6	0	2.866096	1.041831	0.012478
2	6	0	2.452737	-0.285989	0.000460
3	6	0	1.095173	-0.594316	-0.010355
4	6	0	0.154104	0.425815	-0.009077
5	6	0	0.560668	1.760914	0.002843
6	6	0	1.911473	2.055007	0.013583
7	1	0	3.915829	1.299018	0.021118
8	1	0	0.792756	-1.632626	-0.019629
9	1	0	-0.178241	2.550579	0.003808
10	1	0	2.239578	3.087157	0.023156
11	6	0	-1.300643	0.119998	-0.019564
12	8	0	-2.152486	0.999534	-0.021852
13	8	0	-1.588182	-1.165219	-0.026965
14	1	0	-2.580684	-1.347771	-0.032691
15	8	0	-4.124803	-1.804859	-0.079357
16	1	0	-4.602747	-0.949403	-0.004263
17	1	0	-4.379113	-2.321232	0.695044
18	8	0	-4.913454	0.791679	0.118788
19	1	0	-5.327732	1.192456	-0.653922
20	1	0	-3.954961	0.965612	0.028964
21	8	0	3.304131	-1.352701	-0.001717
22	6	0	4.707043	-1.095743	0.008620
23	1	0	5.187282	-2.071493	0.004451
24	1	0	5.008633	-0.535938	-0.879552
25	1	0	4.997853	-0.548916	0.908410

Rotational constants (GHZ): 1.7374381 0.3044498 0.2598210

3-Metoxybenzoate

Electronic Energy -687.784309
Free Energy -687.643669
Stoichiometry C8H11O5(1-)
Framework group C1[X(C8H11O5)]
Deg. of freedom 66
Full point group C1 NOp 1
Largest Abelian subgroup C1 NOp 1
Largest concise Abelian subgroup C1 NOp 1
Standard orientation:

Center Number	Atomic Number	Atomic Type	Coordinates (Angstroms) X	Y	Z
1	6	0	-2.833574	1.042319	-0.015976
2	6	0	-1.866749	2.043160	-0.063083
3	6	0	-0.517837	1.733534	-0.073039
4	6	0	-0.115280	0.397311	-0.036606
5	6	0	-1.072407	-0.605631	0.009269
6	6	0	-2.427993	-0.286904	0.019828
7	1	0	-3.880972	1.309078	-0.008466
8	1	0	-2.183790	3.078930	-0.091827
9	1	0	0.225776	2.517751	-0.109425
10	1	0	-0.775314	-1.645832	0.037338
11	6	0	1.354057	0.037150	-0.045506
12	8	0	1.658866	-1.182965	-0.025666
13	8	0	2.183132	0.986541	-0.071596
14	8	0	4.296716	-1.926003	-0.123474
15	1	0	3.340590	-1.696350	-0.097356
16	1	0	4.722473	-1.056039	-0.024445
17	8	0	4.867528	0.830131	0.127625
18	1	0	3.880626	0.881570	0.078127
19	1	0	5.098226	1.134246	1.012298
20	8	0	-3.289981	-1.348480	0.067082
21	6	0	-4.689331	-1.078319	0.070850
22	1	0	-4.989495	-0.551438	-0.837983
23	1	0	-5.179934	-2.048426	0.107132
24	1	0	-4.973581	-0.492994	0.948411

Rotational constants (GHZ): 1.7267938 0.3052022 0.2603155

4-Metoxybenzoic Acid

Electronic Energy -688.245117
Free Energy -688.091183
Stoichiometry C8H12O5
Framework group C1[X(C8H12O5)]
Deg. of freedom 69
Full point group C1 NOp 1
Largest Abelian subgroup C1 NOp 1
Largest concise Abelian subgroup C1 NOp 1
Standard orientation:

Center Number	Atomic Number	Atomic Type	Coordinates (Angstroms) X	Y	Z
1	6	0	-2.844676	0.234220	0.006450
2	6	0	-2.097906	1.415343	0.013691
3	6	0	-0.721183	1.360674	0.002968
4	6	0	-0.060793	0.127361	-0.014491
5	6	0	-0.815162	-1.042177	-0.021632
6	6	0	-2.200041	-1.001432	-0.011770
7	1	0	-2.618558	2.364721	0.027815
8	1	0	-0.151042	2.279805	0.008816
9	1	0	-0.312538	-2.000660	-0.035205
10	1	0	-2.761676	-1.924432	-0.018011
11	6	0	1.412320	0.035860	-0.023839
12	8	0	2.010830	-1.035062	-0.030481
13	8	0	2.031214	1.203008	-0.024914
14	1	0	3.033351	1.113010	-0.035161
15	8	0	4.656759	1.143694	-0.095385
16	1	0	4.887988	0.194376	0.009614
17	1	0	5.041434	1.599051	0.663007
18	8	0	4.721489	-1.564115	0.147815
19	1	0	5.015812	-2.061602	-0.623843
20	1	0	3.752139	-1.469822	0.050606
21	8	0	-4.192212	0.384373	0.018078
22	6	0	-5.008934	-0.787403	0.013937
23	1	0	-6.036111	-0.431107	0.025979
24	1	0	-4.836208	-1.378239	-0.887713
25	1	0	-4.820988	-1.394957	0.901310

Rotational constants (GHZ): 2.3158494 0.2713487 0.2435860

4-Metoxybenzoate

Electronic Energy -687.785726
Free Energy -687.645349
Stoichiometry C8H11O5(1-)
Framework group C1[X(C8H11O5)]
Deg. of freedom 66
Full point group C1 NOp 1
Largest Abelian subgroup C1 NOp 1
Largest concise Abelian subgroup C1 NOp 1

Standard orientation:

Center Number	Atomic Number	Atomic Type	Coordinates (Angstroms) X	Y	Z
1	6	0	-2.806789	0.279718	-0.000731
2	6	0	-2.011464	1.426517	-0.014764
3	6	0	-0.635644	1.311678	-0.016856
4	6	0	-0.018477	0.058012	-0.005136
5	6	0	-0.825838	-1.073895	0.008728
6	6	0	-2.211746	-0.978230	0.011056
7	1	0	-2.490831	2.397966	-0.023974
8	1	0	-0.026371	2.205981	-0.027909
9	1	0	-0.366125	-2.053646	0.017748
10	1	0	-2.808660	-1.879232	0.021809
11	6	0	1.481249	-0.065393	-0.007916
12	8	0	1.977103	-1.224023	0.003304
13	8	0	2.156599	1.001311	-0.021809
14	8	0	4.685677	-1.574652	-0.012338
15	1	0	3.705945	-1.476155	-0.006809
16	1	0	4.989388	-0.650386	-0.008683
17	8	0	4.838896	1.254309	-0.056321
18	1	0	3.854565	1.154893	-0.018953
19	1	0	5.084575	1.712583	0.754734
20	8	0	-4.153894	0.485991	0.000383
21	6	0	-5.011718	-0.653701	0.014407
22	1	0	-6.026267	-0.261928	0.013133
23	1	0	-4.856809	-1.270066	-0.873751
24	1	0	-4.851908	-1.251733	0.914157

Rotational constants (GHZ): 2.3408247 0.2714260 0.2437506
